# Inequalities in multimorbidity between native-born and immigrant older adults across Europe

**DOI:** 10.1007/s10433-025-00879-5

**Published:** 2025-08-14

**Authors:** Su Yeon Jang, Silvia Loi, Frank J. van Lenthe, Anna Oksuzyan, Mikko Myrskylä

**Affiliations:** 1 Max Planck Institute for Demographic Research, Rostock, Germany; 2 Department of Public Health, Erasmus MC University Medical Center, Rotterdam, Netherlands; 3 Max Planck - University of Helsinki Center for Social Inequalities in Population Health, Rostock, Germany; 4 School of Public Health, Bielefeld University, Bielefeld, Germany; 5 Center for Social Data Science and Population Research Unit, University of Helsinki, Helsinki, Finland; 6 Oxford Institute of Population Ageing, University of Oxford, Oxford, United Kingdom

**Keywords:** Migrants, Multimorbidity, Chronic diseases, Europe

## Abstract

Immigrants with a chronic disease may have a higher burden of multimorbidity than their native-born counterparts due to the unique experiences in their origin and the receiving countries. In this study, we provide a descriptive overview of inequalities in multimorbidity between immigrant and native-born older adults with chronic diseases in Europe. Our analysis includes individuals aged 50–79 years who participated in the Survey of Health, Ageing, and Retirement in Europe (SHARE) from Waves 2 through 9. We first estimate the prevalence of multimorbidity among person-years with each of eight chronic diseases and then compare the prevalence between native-born and immigrant populations by computing the relative risk. Overall, immigrants with chronic diseases have a higher prevalence of multimorbidity compared to native-born individuals with the condition, a trend typically more pronounced among women. In particular, both immigrant men and women have a pronounced risk of multimorbidity with stomach ulcers compared to their native-born counterparts. In subgroup analyses by regions of origin and residence, we find that immigrants from Eastern Europe or Asia/Oceania and those living in Northern Europe have particular disadvantages in multimorbidity to their native-born counterparts, especially for disease combinations that include stomach ulcers. Our findings can help identify the target populations and health conditions that should be prioritized in efforts to reduce health disparities between native-born and immigrant older adults in Europe.

**Supplementary Information:**

The online version contains supplementary material available at 10.1007/s10433-025-00879-5.

## Introduction

Over recent decades, individuals of diverse backgrounds have crossed European borders, resulting in nearly 87 million immigrants across Europe today (International Organization for Migration 2024). As many of these immigrants are growing older in their receiving countries, it becomes increasingly important to understand their health needs in later life. In particular, older immigrants likely have specific health needs that are different from native-born populations due to their unique life experiences before, during, and after migration (Kristiansen et al. 2016). Importantly, these experiences are highly heterogeneous, even among immigrants themselves, based on immigration policies, healthcare systems, and social norms to which they are exposed (Kristiansen et al. 2016). The differential experiences of immigrants add extra layers of protection from or increased risk of adverse health outcomes, which cumulatively shape distinct health profiles in later life that differ not only from the native-born population but also between immigrant subgroups (Spallek et al. 2011; Jang et al. 2023).

Multimorbidity, defined as the coexistence of two or more chronic health conditions in an individual, poses a significant health challenge for aging populations. Older adults with multimorbidity often experience an accelerated deterioration of health, frailty, and mortality, contributing to the elevated burden on the healthcare systems of society (Barnett et al. 2012). Although older adults generally experience a heightened risk for several chronic diseases, the speed at which these conditions accumulate and develop into multimorbidity varies widely between populations based on individual and social contexts (Barnett et al. 2012; Dekhtyar et al. 2019; Cezard et al. 2021). This variability suggests that individuals from certain backgrounds may be more likely to have multimorbidity once a chronic disease is present, resulting in distinct prevalence across populations.

The aim of this study is to provide a descriptive overview of inequalities in multimorbidity between immigrant and native-born older adults with chronic health conditions in Europe. In particular, we focus on how these patterns manifest differently across immigrant subgroups based on the regions to which their country of origin and receiving country belong. To this end, we estimate the prevalence of multimorbidity, including specific additional conditions, among immigrants and native-born individuals with chronic diseases across Europe. Findings from this study can help identify which immigrant subgroups—particularly those burdened with specific chronic health conditions—are most affected by health inequalities relative to native-born populations, thereby offering valuable insights to inform targeted healthcare policies and interventions.

### Immigrant health and multimorbidity

In general, immigrants are often observed to be in better health than the non-migrant population as they tend to have considerable physical and mental resilience needed to overcome the challenges of the migration process (Abraído-Lanza et al. 1999; Palloni and Arias 2004). However, this phenomenon of the “healthy immigrant effect” tends to dilute over time spent in the receiving country as immigrants often adapt to the unhealthy behaviors of the native-born population, such as smoking or alcohol consumption (Gubernskaya 2015; Bousmah et al. 2019). Moreover, immigrants are more likely to go through adverse life events, including job loss or divorce, contributing to their loss of the initial health advantage (Loi et al. 2024).

This pattern of initial health advantage followed by convergence with native-born level extends to chronic diseases and multimorbidity as well. Research indicates that immigrants generally have a lower risk of multimorbidity compared to native-born populations (Diaz et al. 2015b; Lenzi et al. 2016), though this advantage tends to converge with the native-born level over time (Diaz et al. 2015a; Gimeno-Feliu et al. 2017). In fact, older immigrants who have spent considerable time in the receiving country may actually develop more chronic health conditions than native-born individuals (Jang et al. 2023). This burden of chronic diseases in older age is particularly pronounced for immigrant women, even when care and support are available (Jang et al. 2025). In addition, the burden of chronic diseases and multimorbidity risk increases more markedly for immigrants from Asian countries and those living in Western and Northern Europe over time (Gimeno-Feliu et al. 2017; Jang et al. 2023).

### Subgroup differences in multimorbidity patterns

Prior research has shown that patterns of multimorbidity vary by country, gender, age, race/ethnicity, and socioeconomic status (Prados-Torres et al. 2012; Barnett et al. 2012; Ioakeim-Skoufa et al. 2020; Lu et al. 2021). The risk of specific chronic disease combinations varies even within Europe, with a higher concurrent presence of cardiovascular and metabolic diseases in Southern Europe and a higher concurrent presence of cancer and cardiovascular diseases in Eastern Europe (Bayes-Marin et al. 2020; Álvarez-Gálvez et al. 2022). In addition, the risk of simultaneously having cardiovascular and metabolic diseases is higher among women and older individuals than among men and younger individuals (Prados-Torres et al. 2012; Abad-Díez et al. 2014). There is also evidence that African and Native Americans have a higher risk of co-occurring nicotine-dependent diseases and hypertension compared to Caucasian Americans, especially at younger ages, and that highly deprived individuals face a higher risk than less deprived individuals of having any chronic disease combination, including those involving pain, depression, or anxiety (McLean et al. 2014; Alshakhs et al. 2022). These results indicate that among individuals with chronic health conditions, the patterns of which specific diseases may co-occur more frequently vary considerably across populations. However, research that has specifically examined such patterns of multimorbidity among immigrants remains scarce.

### Theoretical framework

One widely accepted framework for understanding health disparities is the cumulative disadvantage theory, which posits that individuals with disadvantaged social and structural backgrounds may be more likely to face additional disadvantages, which compound and may lead to adverse health outcomes in later life (Dannefer 2003; Willson et al. 2007). When applied to immigrant health, this perspective suggests that immigrants from a certain country of origin or those who arrived in a specific receiving country may experience layered disadvantages that cumulatively worsen health (Spallek et al. 2011). Once a chronic disease is present, these disadvantages may limit access to resources necessary for effective disease management and prevention, increasing the risk of multimorbidity. For instance, immigrants often face unique challenges in managing existing conditions and preventing new conditions due to limited healthcare access, reduced welfare benefits, and cultural barriers (Solé-Auró et al. 2012; Lebrun 2012; Juárez et al. 2019). While we do not directly test the cumulative disadvantage hypothesis, this theoretical perspective offers solid and plausible interpretative insights, in line with the previous literature (Gubernskaya 2015; Loi et al. 2025). From this theoretical perspective, we expect to find a heightened risk of multimorbidity for immigrants in the presence of chronic disease, especially when they are from culturally distant non-European countries or living in countries implementing relatively passive immigrant integration policies.

## Methods

### Data and study population

This study uses data from the Survey of Health, Ageing, and Retirement in Europe (SHARE), a panel study on the health and socioeconomic status of individuals aged 50 and above in 28 European countries and Israel. We include Waves 2, 4, 5, 6, 8, and 9 (2006–2020) in our analysis while excluding Wave 1 (*n* = 30,416) and Wave 3 (*n* = 28,454) due to the lack of information on the diagnosis of dementia and the current medical issues, respectively. Participants younger than age 50 (*n* = 11,264), who are mainly the spouses of the core study participants, are omitted from the analysis, given that multimorbidity is more common among older adults (Barnett et al. 2012). We also consider that because immigrants with health problems can return to their origin countries, it may appear that immigrant populations are healthier than their native-born counterparts at older ages (Palloni and Arias 2004). To reduce the potential issues from such return migration bias, we exclude participants aged 80 years and older (*n* = 12,687) from the analysis. Furthermore, since the geographical focus of this paper is on Europe, individuals who participated in Israel are excluded (*n* = 3,051). After removing participants with missing information on migration-related variables (*n* = 696) and present chronic conditions (*n* = 543), our final analytical sample includes a total of 289,584 observations from 113,470 participants.

### Chronic diseases and multimorbidity

The main outcome of this study is multimorbidity, which we define as the co-occurrence of two or more chronic diseases. We focus on 14 chronic health conditions that appear with high to moderate frequency in multimorbidity research (Hafezparast et al. 2021), ensuring consistency with previous studies. This does not include chronic kidney disease despite its common inclusion in prior work, as it was included in the survey only in recent years, providing insufficient data. Among these selected chronic diseases, we combine conditions with overlapping clinical characteristics into broader categories to avoid overestimation of multimorbidity. In doing so, we arrive at eight final disease groups: cardiovascular diseases, diabetes, respiratory diseases, musculoskeletal diseases, mental disorders, stomach ulcers, dementia and Parkinson’s, and cancer (see Table S1).

Having identified these eight chronic diseases, we establish their presence in each person-year of our analytical sample based on self-reported doctor diagnoses. For mental disorders, we additionally count individuals who have been treated for or hospitalized with depression as having the condition. Furthermore, we consider individuals who are currently taking drugs for each condition as having the disease, given that patients on medications may have attenuated symptoms, underreporting their medical issues. Finally, this study considers chronic diseases to be irreversible and, therefore, defines individuals with a history of ever having had a condition as having the condition. In addition to noting the presence of each chronic disease, we also count the number of chronic diseases in each individual at each survey wave and define those individuals who have multiple conditions simultaneously as having multimorbidity.

### Immigration background

In this study, the term immigrant refers to individuals born in a foreign country. To examine regional variations in multimorbidity patterns, we group immigrants by the regions to which their country of origin and the receiving country belong—hereafter referred to as region of origin and region of residence, respectively. The region of origin is defined as the broader geographical region corresponding to the immigrants’ country of birth. The region of residence refers to the region corresponding to the country in which the immigrant is currently living and is giving the interview. Both regions are categorized into geographical subgroups based on the geo-scheme by the United Nations (2021), with some modifications (see Table S1 for the detailed list of countries). The region of origin includes five subgroups (Africa, Asia and Oceania, Latin America and the Caribbean, Eastern Europe, and other Europe and North America). The region of residence is categorized into four European subgroups (Northern, Western, Southern, and Eastern Europe). Notable exceptions include Cyprus, which is reassigned to Southern Europe, and the former Soviet and Yugoslav republics, which are assigned to Eastern Europe regardless of their current borders.

### Statistical analysis

We perform all our analyses in samples stratified by gender in the following steps. First, we present crude frequencies and percentages for the descriptive characteristics of our analytical sample by immigrant status and use Chi-square tests to evaluate differences in these characteristics between native-born and immigrant individuals. Second, we estimate the total person-years affected by each of the eight chronic diseases included in our study by computing their prevalence over the pooled study period. Third, we measure the burden of multimorbidity among those affected by each chronic health condition—the primary focus of our study—by calculating the prevalence of additional chronic diseases among the total person-years that are lived with a chronic condition as in prior research (Barnett et al. 2012). In other words, the prevalence is computed as the conditional probability of having multimorbidity, given that the individual already has a chronic condition. We estimate the prevalence separately for (i) any additional chronic disease and (ii) each specific additional disease in order to capture the overall multimorbidity and specific disease combinations, respectively. To ensure reliable estimates, we conduct these analyses only when we observe at least 30 person-years with the chronic condition under consideration. Finally, we assess inequalities in multimorbidity between native-born and immigrant populations in the presence of chronic health conditions by estimating the relative risk of multimorbidity.

For the prevalence and comparison of multimorbidity, we additionally perform subgroup analyses on samples stratified by 10-year age groups, region of origin, and region of residence. We age-standardize all prevalence estimates, except for those in age-specific subgroups, using the 2013 European Standard Population (Eurostat 2013). All 95% confidence intervals are gained from the bootstrapping based on the binomial assumption (1000 repetitions).

### Supplemental analyses

We perform supplemental analyses to help with the interpretation and test the robustness of the results. First, although our descriptive analysis captures the real-world burden of multimorbidity among immigrants, it cannot distinguish whether the estimated burden is more related to the immigration background itself or to the sociodemographic characteristics of immigrants. Therefore, we use Poisson regression models to estimate adjusted risk ratios for multimorbidity between immigrants and native-born individuals, controlling for sociodemographic factors. Our model includes immigration status, 10-year age groups, education, household income, employment status, marital status, country, and the survey wave (detailed variable definitions in Table S1). Standard errors are clustered at the individual level to account for the longitudinal structure of the data.

Second, when immigrants show a higher multimorbidity risk compared to native-born individuals, it remains unclear whether the elevated risk is attributable to high prevalence upon arrival or to a higher likelihood of developing an additional condition throughout their stay in the receiving country. Consequently, our subgroup analyses face interpretational challenges in understanding why immigrants from a particular origin or those living in specific receiving countries may have high multimorbidity risks. We address these challenges by restricting our subgroup analyses to conditions developed after migration. We compare the self-reported year of diagnosis and the migration year to determine whether the diagnosis is before or after migration.

Third, our analytical decision to exclude individuals aged 80 and above in the analysis may provide a skewed description of the risk for age-related conditions, such as dementia. Because these conditions are more likely to occur at older ages, removing samples at older ages can underestimate their overall burden and result in inaccurate estimates. Therefore, we run additional analyses where the older samples are included to address potential underestimation of age-related conditions.

Lastly, while some countries participated in the survey from the start of our inclusion period, several countries joined the study in later waves. This unbalanced panel may have resulted in an overrepresentation of trends in countries that contributed to more waves in our findings. We perform a sensitivity analysis restricting the sample to countries participating in all waves from Wave 7 onward, which includes all countries but Ireland, the Netherlands, and Portugal. We choose to restrict the analysis to later waves rather than from Wave 2, as the countries that have participated in SHARE from earlier waves are largely in Western Europe, resulting in a less representative picture.

## Results

### Descriptive characteristics

Table 1 summarizes the key demographic and health characteristics of the analytical sample in our study. Our immigrant observations in their fifties outnumber their native-born peers for both genders. The study participants mainly live in Western Europe regardless of their gender and immigrant status, with the largest shares of both male and female immigrants being born in other European regions. Immigrants exhibit a higher prevalence of chronic diseases and multimorbidity than native-born individuals in the 50–59 age group, but in the 70–79 age group, the only gap that remains is a difference in multimorbidity prevalence among women. A detailed cross-tabulation of specific chronic disease presence between native-born and immigrant samples is available in Table S2.

**Table 1:** Descriptive characteristics of the analytical sample

	Men		Women	
	Native-born	Immigrant	Native-born	Immigrant
N person-years	142,287	12,781	178,495	16,929
Age group				
50–59	37,436 (26.3%)	3764 (29.4%)***	52,334 (29.3%)	5383 (31.8%)***
60–69	59,635 (41.9%)	4960 (38.8%)***	71,497 (40.1%)	6319 (37.3%)***
70–79	45,216 (31.8%)	4057 (31.7%)	54,664 (30.6%)	5227 (30.9%)
Region of origin				
Africa	– (–)	1168 (9.1%)	– (–)	1246 (7.4%)
Asia and Oceania	– (–)	846 (6.6%)	– (–)	957 (5.7%)
Latin America and the Caribbean	– (–)	318 (2.5%)	– (–)	511 (3.0%)
Eastern Europe	– (–)	3942 (30.8%)	– (–)	6154 (36.4%)
Other Europe and North America	– (–)	6507 (50.9%)	– (–)	8061 (47.6%)
Region of residence				
Northern Europe	17,800 (12.5%)	926 (7.2%)***	20,387 (11.4%)	1283 (7.6%)***
Western Europe	48,693 (34.2%)	6178 (48.3%)***	58,638 (32.9%)	7736 (45.7%)***
Southern Europe	40,415 (28.4%)	2550 (20.0%)***	50,376 (28.2%)	3102 (18.3%)***
Eastern Europe	35,379 (24.9%)	3127 (24.5%)	49,094 (27.5%)	4808 (28.4%)*
Have any chronic disease				
Among 50–59	21,990 (15.5%)	2310 (18.1%)***	33,178 (18.6%)	3642 (21.5%)***
Among 60–69	45,917 (32.3%)	3968 (31.0%)**	58,396 (32.7%)	5329 (31.5%)**
Among 70–79	39,991 (28.1%)	3580 (28.0%)	50,401 (28.2%)	4869 (28.8%)
Have multimorbidity				
Among 50–59	9302 (6.5%)	1089 (8.5%)***	16,393 (9.2%)	2020 (11.9%)***
Among 60–69	25,176 (17.7%)	2329 (18.2%)	36,893 (20.7%)	3656 (21.6%)**
Among 70–79	26,293 (18.5%)	2421 (18.9%)	37,894 (21.2%)	3824 (22.6%)***

*p < 0.05; **p < 0.01; ***p < 0.001

### Prevalence of chronic diseases and multimorbidity

**Fig. 1:**
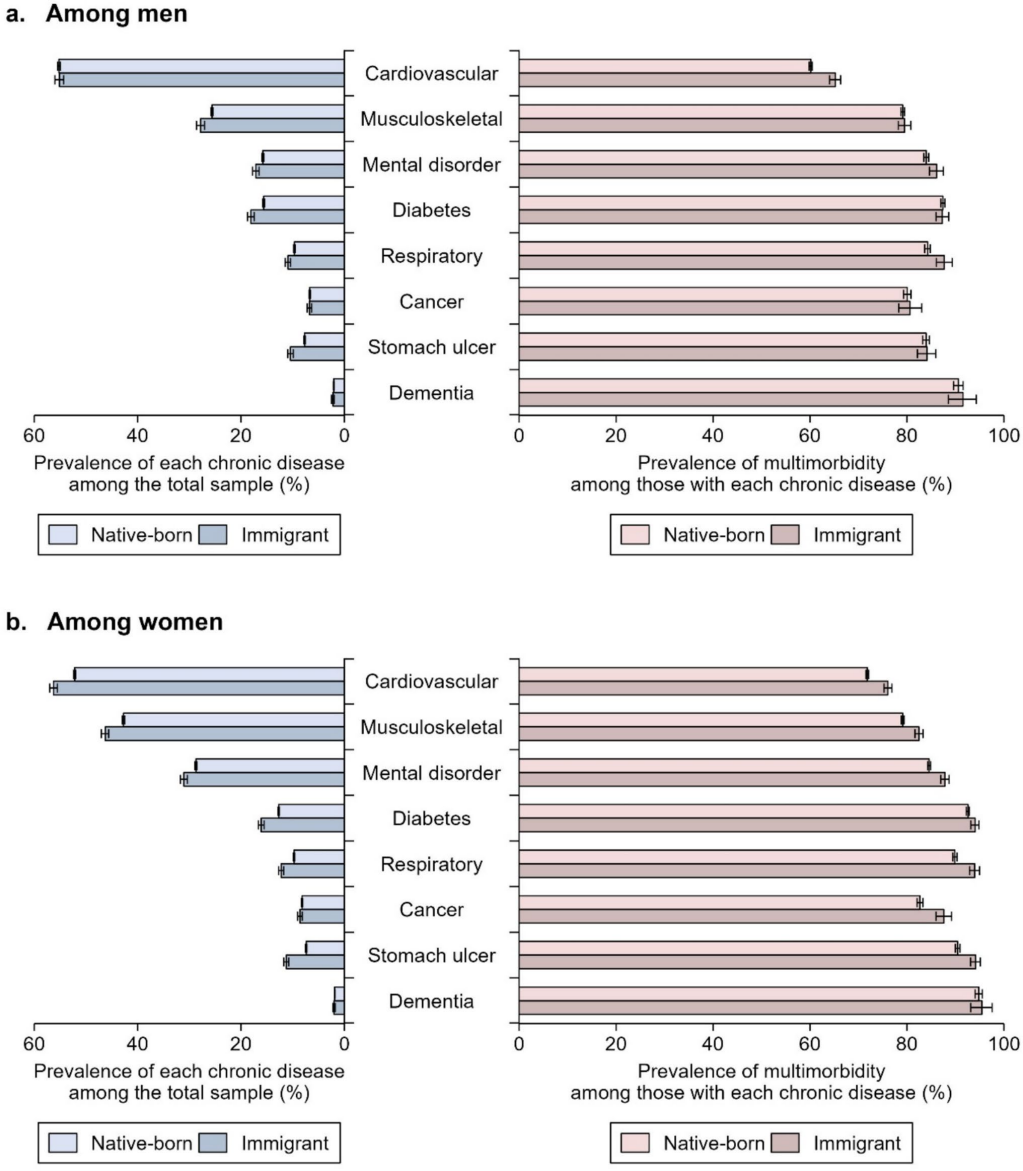
Age-standardized prevalence of eight chronic diseases and multimorbidity among native-born and immigrant individuals **a** among men; **b** among women

Figure 1 presents the age-standardized prevalence of chronic diseases and multimorbidity (see Table S3 for details). Immigrants generally have a higher prevalence of chronic diseases. Exceptions are cancer and dementia in both genders and cardiovascular diseases among men, in which the prevalence is similar between immigrants and native-born individuals. This immigrant health disadvantage is also observed in the risk of multimorbidity among individuals with each condition, but it is typically more pronounced in women (panel b) than in men (panel a). Furthermore, we find notable discrepancies between the prevalence of single chronic diseases and of multimorbidity among individuals with each of these conditions. For instance, while immigrant men have a higher prevalence of diabetes than their native-born counterparts, the risk of multimorbidity in individuals with diabetes is similar for immigrant and native-born men.

### Disparities in multimorbidity patterns between native-born and immigrant populations

**Fig. 2:**
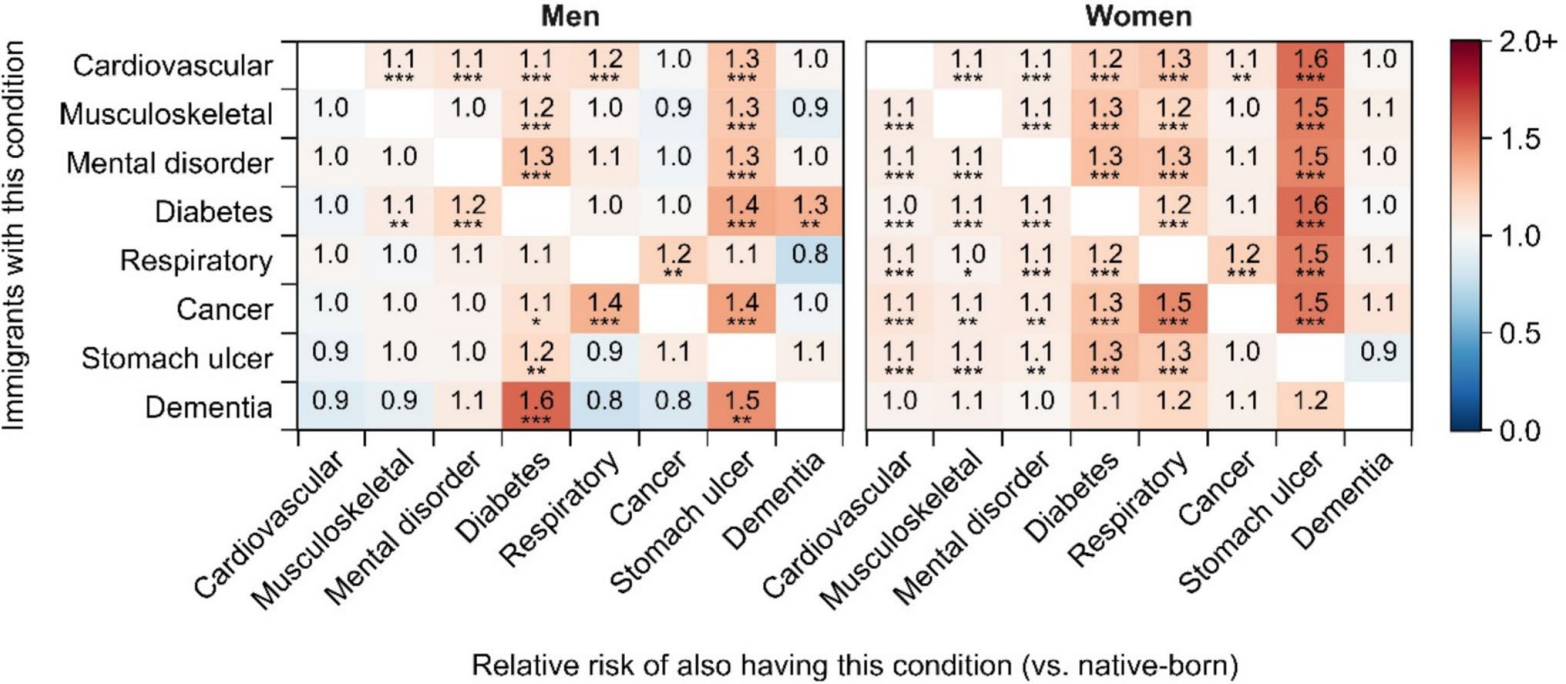
Relative risks of multimorbidity among immigrants versus native-born individuals by gender. *p < 0.05; **p < 0.01; ***p < 0.001

Figure 2 shows the relative risk of multimorbidity for each chronic disease combinations among immigrants versus native-born individuals by gender (detailed results in Table S4). We find that the relative risk of most chronic disease combinations is near 1.0 among men, suggesting a fairly equal multimorbidity burden between immigrants and native-born individuals, although the risk becomes notably higher for multimorbidity with stomach ulcers. Among women, immigrants have a higher prevalence of multimorbidity for most disease combinations compared to native-born women, except for multimorbidity with dementia. Similar to men, immigrant women experience a particularly pronounced disadvantage in multimorbidity with stomach ulcers.

**Fig. 3:**
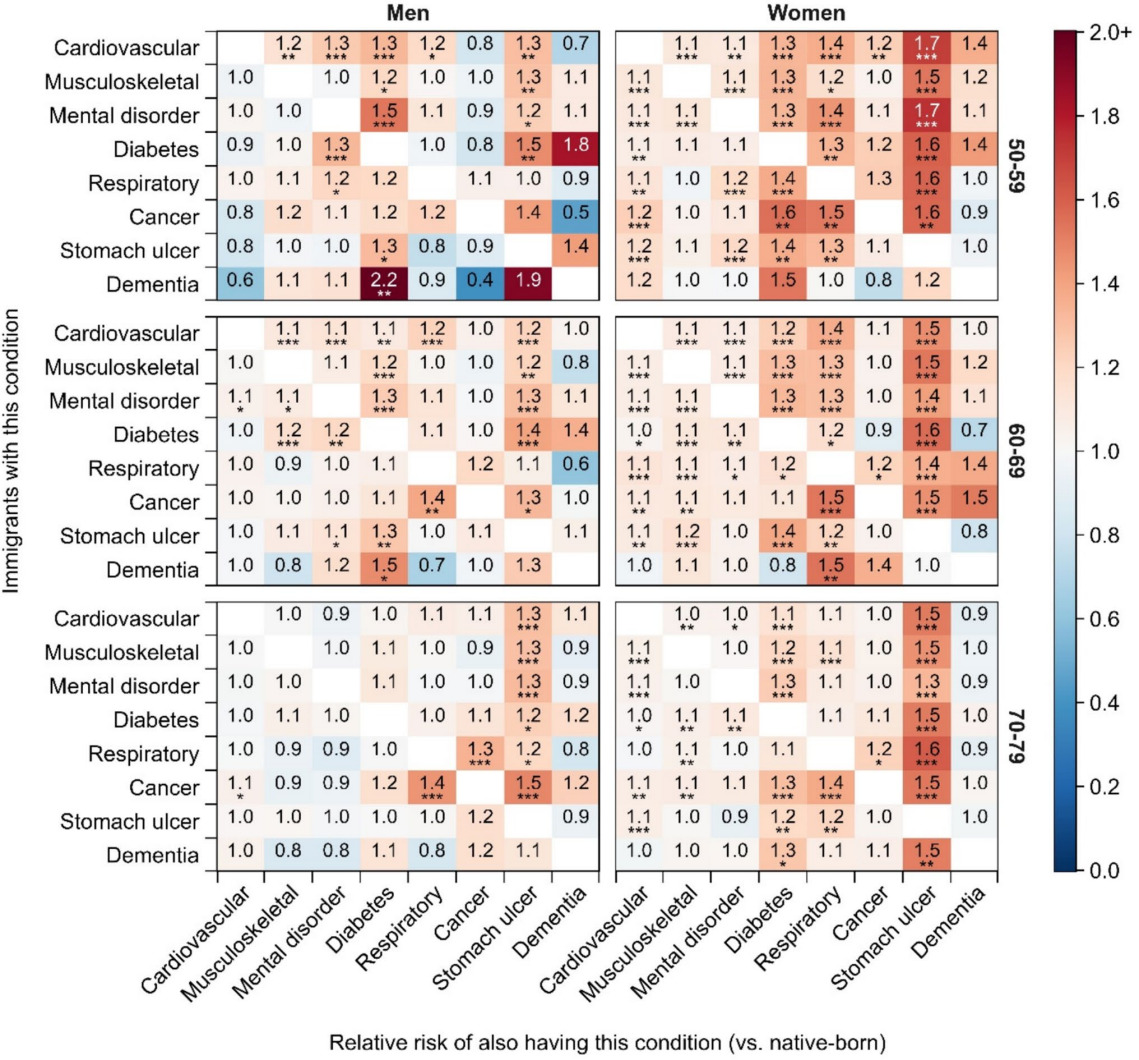
Relative risks of multimorbidity among immigrants versus native-born individuals by gender and age group. *p < 0.05; **p < 0.01; ***p < 0.001

Figure 3 illustrates the relative risk of multimorbidity among immigrants versus among native-born individuals by gender and age group (detailed results in Table S5). Similar to the gender-specific patterns, we find considerable immigrant disadvantages for most disease combinations among women across all age groups, which becomes less apparent among men. At ages 50–59, immigrant men with chronic diseases face a lower risk of cardiovascular diseases or cancer and a higher risk of diabetes or stomach ulcers compared to their native-born counterparts. These disparities between immigrant and native-born men dissipate at older ages, except for the prevalence of stomach ulcers, for which an immigrant disadvantage remains. Similarly, the immigrant disadvantage in multimorbidity risk among women tends to decline with age for several chronic disease combinations, such as the combinations including diabetes or chronic respiratory diseases.

### Regional variation in multimorbidity among immigrants

**Fig. 4:**
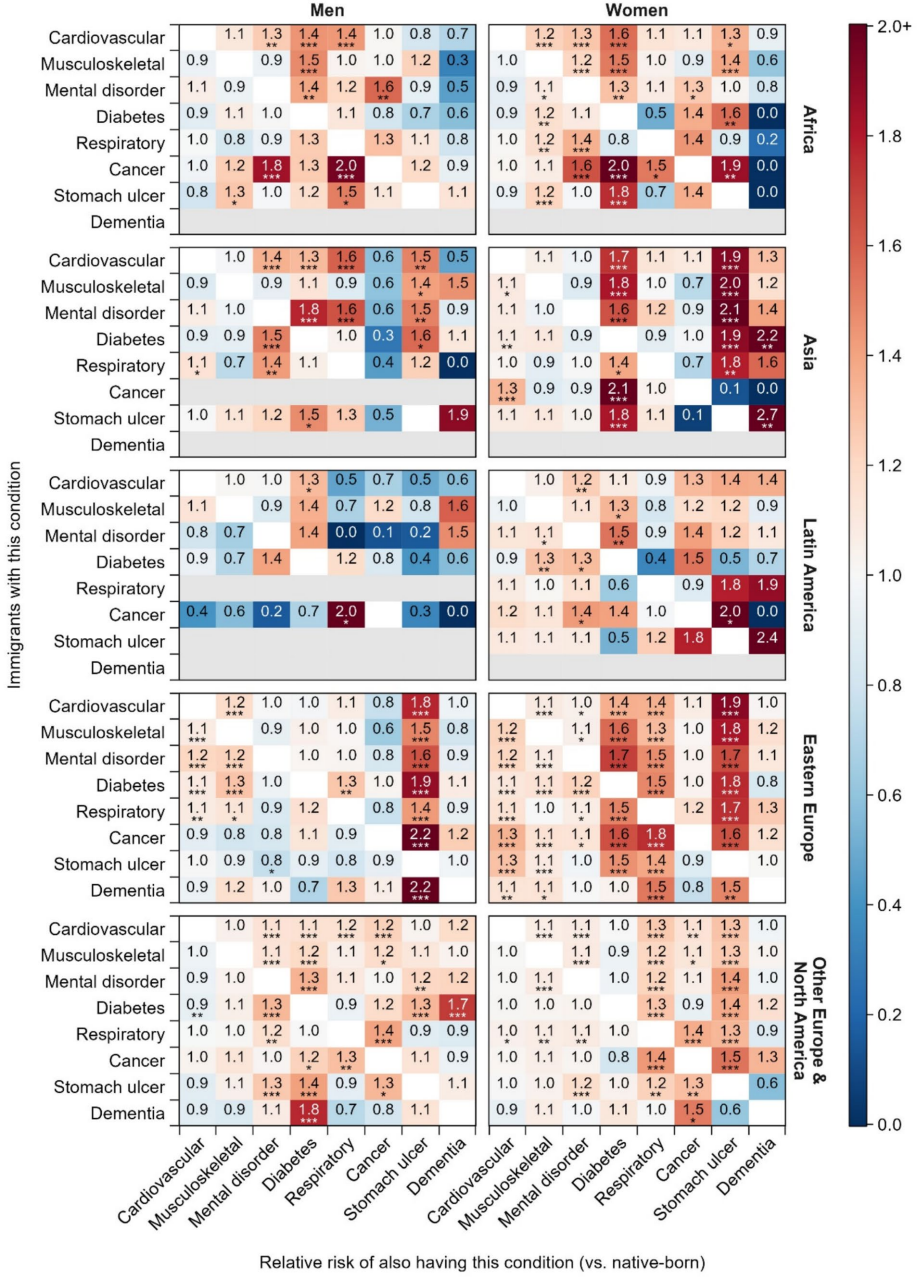
Relative risks of multimorbidity among immigrants versus native-born individuals by gender and region of origin. *p < 0.05; **p < 0.01; ***p < 0.001

Figure 4 shows findings from the subgroup analyses by region of origin (detailed estimates in Table S6). In general, immigrants have a higher prevalence of the most chronic disease combinations than native-born individuals among women from Eastern Europe, while it is difficult to find a uniform pattern in the other subgroups. Notably, the patterns of immigrant-native disparities for chronic disease combinations, including those involving stomach ulcers, vary across region of origin. Immigrant men from the Americas with any sort of chronic disease have a lower risk of developing stomach ulcers compared to their native-born counterparts, while both immigrant men and women from Asia and Oceania, Eastern Europe, and other Europe, regardless of gender, have a higher risk of developing stomach ulcers compared to their native-born counterparts, with only a few exceptions.

**Fig. 5:**
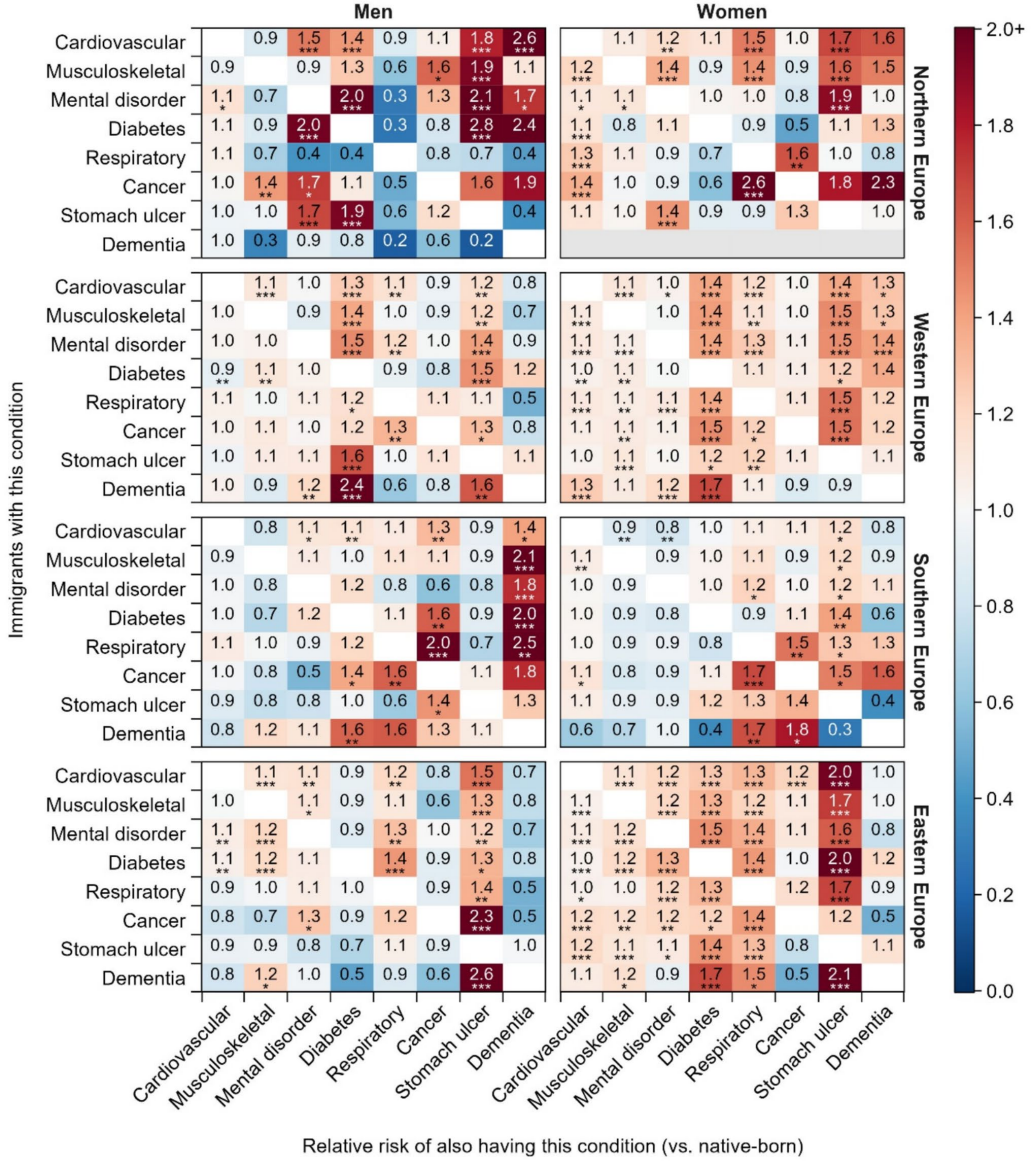
Relative risks of multimorbidity among immigrants versus native-born individuals by gender and region of residence. *p < 0.05; **p < 0.01; ***p < 0.001

We observe varying patterns of inequalities in multimorbidity between immigrants and their native-born counterparts by the region of residence (Fig. 5; details in Table S7). In particular, the probability of having stomach ulcers when any other chronic disease is present is significantly higher among immigrants than among native-born individuals across all genders and regions, except among men living in Southern Europe. This immigrant disadvantage is particularly pronounced among immigrants living in Northern and Eastern Europe.

### Findings from supplemental analyses

Additional analyses for better interpretation of our findings show that the risks of multimorbidity among immigrants become less pronounced after adjusting for socioeconomic and demographic characteristics for women (Fig. S1; Tables S8–S9). These results suggest that socioeconomic positions may partially explain the multimorbidity burden among immigrant women. Moreover, when restricting the analysis to only the conditions diagnosed after migration, the relatively high risk of stomach ulcer-related multimorbidity among immigrants from Asia and Eastern Europe and those in Northern and Eastern Europe becomes considerably lower (Table S10; Figs. S2–S3). Therefore, the higher risk of multimorbidity among these immigrant subgroups may partly be due to their high stomach ulcer diagnoses prior to migration.

We also test the robustness of our findings. When including the samples in the excluded age range of 80 years and above, the relative risks of multimorbidity among immigrants become smaller for several combinations—particularly those involving diabetes and stomach ulcers (prevalence in Table S11; relative risks in Fig. S4). These additional findings show that our immigrant sample consists of relatively healthy individuals at older ages, hinting a selection that unhealthy immigrants are more likely to drop out of the survey as they age due to death or return migration. Furthermore, when restricting the analysis to countries that participated in all waves from Wave 7 onward, the relative risks of multimorbidity among immigrants versus native-born individuals slightly increase in magnitude (Fig. S5). The slight increase likely reflects the balanced representation of Eastern European countries in this restricted sample, where immigrants experience larger health inequalities compared to Western European countries. However, the overall consistency in the pattern supports the robustness of our findings despite variations in country participation across SHARE waves.

## Discussion

### Summary of findings

Findings from this study reveal that inequalities in multimorbidity between native-born and immigrant older adults in Europe can be amplified for specific disease combinations and also in certain subgroups by gender, age, country of origin, and receiving country. For instance, we find that multimorbidity that includes stomach ulcers is more common among immigrants than among native-born individuals of both genders. We also find that the risk of multimorbidity is particularly pronounced among women than among men. Broken down by the country of origin and the receiving country, our study shows that immigrants face a higher risk of multimorbidity, especially with stomach ulcers, when they are from Eastern Europe or Asia/Oceania and when they are living in Northern Europe. Our findings on the different magnitudes of the inequalities between native-born and immigrant populations based on gender, age, country of origin, and country of destination are in line with the theory that because immigrants have heterogeneous experiences throughout their life course, the health outcomes of immigrants may vary across different subgroups (Spallek et al. 2011).

### Interpretations of findings

Notably, we observe that immigrants with chronic health conditions that are more prevalent among immigrants than among native-born individuals do not necessarily have a higher risk of multimorbidity. For example, while immigrant men have a higher prevalence of diabetes than native-born men, the risk of multimorbidity among diabetes patients is lower for immigrants, although the difference is not statistically significant. In previous studies, disparities in the risk of multimorbidity between immigrants and their native-born counterparts have been well established, with immigrants having an overall advantage (Diaz et al. 2015b; Lenzi et al. 2016; Gimeno-Feliu et al. 2017). However, given the discrepancies we observed between individual chronic diseases and multimorbidity, there is a need to investigate immigrant-native disparities by specific chronic disease combinations, particularly to identify the primary source of the inequalities.

Another finding from our study is that several multimorbidity patterns are much more common among immigrants than among native-born individuals, which underscores the importance of investigating multimorbidity by including specific chronic health conditions in the estimation. In particular, the findings of this study suggest that immigrants have a higher risk of developing stomach ulcers or diabetes. A potential explanation for these findings is that immigrants have different health-related behaviors compared to their native-born counterparts (Abraído-Lanza et al. 2005). Findings from several previous studies have reported that both stomach ulcers and diabetes are closely related to health behaviors, including smoking, alcohol consumption, and an unhealthy diet, and that these relationships exist not only for individual diseases but also for the development of comorbidities based on them (Eastwood 1988; Chou 1994; Haire-Joshu et al. 1999; Howard et al. 2004; DeFronzo et al. 2015; Mozzillo et al. 2017; Yegen 2018; Freisling et al. 2020). Our findings on the immigrant disadvantage for the risk of developing chronic disease combinations that include stomach ulcers and diabetes are well explained by the differential behaviors of native-born and immigrant individuals. However, as these results are solely based on descriptive analysis, further studies are needed to investigate the association between stomach ulcer- or diabetes-related combinations of chronic disease conditions and the health-related behaviors of immigrants.

In terms of subgroup differences, our findings show that the prevalence of chronic disease combinations is, in general, higher among immigrant women than among their native-born counterparts, whereas among men, immigrants have disadvantages for some combinations but advantages for others. The more pronounced multimorbidity burden among women can be explained by stronger health selection among voluntary immigrants compared to those who have relocated for less voluntary reasons (Moullan and Jusot 2014). Previously, gendered migration trajectories have been observed, where men often arrive first in the receiving country for work or education-related reasons, while women migrate as trailing spouses (Caputo et al. 2022). Consequently, immigrant men typically experience more positive health selection than women (Gkiouleka and Huijts 2020), although these migration patterns and, thus, the gendered patterns of multimorbidity are likely to change in the future as the voluntary migration of women increases.

We also observe that disparities between native-born and immigrant individuals decrease with age for certain chronic disease combinations. The existing literature posits that immigrants tend to retain health-related behaviors from their country of origin when they first arrive in the receiving country, but they eventually adopt the norms of the receiving country (Abraído-Lanza et al. 2005). This transition usually means moving from healthier to less healthy behavior. For example, as their length of stay in the receiving country increases, the alcohol consumption and smoking rates of immigrants tend to rise (Razum and Twardella 2002; Abraído-Lanza et al. 2005; Reiss et al. 2015). As a result, while immigrants often arrive in the receiving country in better health compared to the native-born population, their health advantage tends to decline over time spent in the receiving society (Antecol and Bedard 2006; Bousmah et al. 2019). Our results partly conform to this narrative, as we find that immigrants have a lower risk of developing these combinations than their native-born counterparts at younger ages, but that the risk becomes similar at older ages.

Furthermore, our stratified analysis illuminates multimorbidity patterns among immigrants by the origin and the receiving country in comparison with those among the native-born population. One notable finding is that there are regional inequalities in the immigrant-native disparities in stomach ulcer-related combinations. We observe that immigrants have a higher risk of stomach ulcers in the presence of other chronic diseases than their native-born counterparts, especially when they are from Asia or Eastern Europe. In addition, we identify a large immigrant disadvantage for these combinations in Northern Europe. We can interpret these results by examining the health behavior of immigrants, as mentioned above, which differs substantially across countries. In particular, behavioral risks in Asia and Eastern Europe are known to be highly burdening, as opposed to the healthier lifestyle in Northern Europe (Institute for Health Metrics and Evaluation (IHME) 2023a). For instance, one of the common characteristics of the Asian diet is the heavy use of spices and sodium, which can exacerbate stomach conditions (Liu et al. 2015; Kim et al. 2022). Immigrants may have retained some characteristics of the diet from their origin country, bringing with them health risks that reflect a higher burden of peptic ulcer diseases in Asia and Eastern Europe (Institute for Health Metrics and Evaluation (IHME) 2023b). Although our study cannot directly compare immigrants’ health with that of non-migrants in their countries of origin, this perspective offers a plausible explanation for why immigrant disadvantages in stomach ulcer-related combinations become particularly pronounced for individuals coming from these origins. Future research can benefit from comparing health outcomes between migrants and non-migrants from the same countries.

Our findings carry important implications for both clinicians and policymakers. For healthcare providers, it is important to recognize that older immigrants with chronic conditions may be particularly susceptible to comorbid stomach ulcers and diabetes, thereby increasing their risk of multimorbidity (see Fig. 1). Once multimorbidity occurs, managing multiple chronic conditions simultaneously often requires complex treatment regimens, such as the concurrent use of multiple medications (i.e., polypharmacy), which can result in a higher overall treatment burden (Lee et al. 2024). Clinicians should therefore consider proactive identification and early management of these high-risk comorbidities to help reduce clinical complications as well as broader structural health inequalities.

At the policy level, targeted preventive strategies are needed to address the health risks disproportionately experienced by certain immigrant subgroups, including women, those in older age groups, and individuals from Asia or Eastern Europe, or those residing in Northern Europe. Some viable approaches include providing accessible and culturally sensitive healthcare services (Savas et al. 2024), such as multilingual health screenings, interpreter-supported medical consultations, and public health campaigns tailored to diverse cultural backgrounds. These strategies targeted at cultural and structural barriers offer concrete opportunities for intervention and should be prioritized in efforts to reduce structural health inequalities between immigrant and native-born older adults.

### Strengths and limitations

Our approach to analyzing multimorbidity patterns using conditional probabilities aligns with the methodology used by Barnett et al. (2012) in their cross-sectional study of multimorbidity. While alternative approaches such as observed-to-expected prevalence ratios have been used in multimorbidity research (Van Den Bussche et al. 2011; Prados-Torres et al. 2014; Spijker and Rentería 2023), our method allows us to directly compare the burden of additional chronic diseases among immigrants with existing conditions to their native-born counterparts. By calculating the prevalence of multimorbidity given the presence of an existing condition, we can identify which immigrant subgroups face disproportionate risks of developing specific disease combinations.

Despite the benefits of our approach, there are several limitations that should be considered when interpreting the findings. That is, as the study focuses on patterns of multimorbidity, the differences in patterns found between immigrants and native-born individuals do not necessarily reflect a causal relationship between the immigration background and chronic health outcomes. Moreover, it should be noted that we do not consider the order of the onset of chronic health conditions in estimating the prevalence of each chronic disease combination. This is due to the nature of our data. As SHARE contains information on each participant’s medical history in the form of self-reported doctor-diagnosed conditions, it is difficult to determine the exact onset point for each condition. Although SHARE does ask participants to report the exact onset points retrospectively, the trustworthiness of these data cannot be guaranteed, as 47% of the total observations have missing information on the variable. Moreover, as many chronic diseases develop over time and exist on a spectrum rather than as a binary outcome of existence/nonexistence, it is challenging to determine which condition started developing before the other. However, as some chronic diseases are known risk factors for others in terms of their pathological mechanisms, the sequence of the development of these conditions is still a valid topic for future research, especially among immigrant populations, as their biological traits and the etiology may differ from those among the native-born group.

Our study faces several data-related limitations as well. First, our study faces limitations in capturing the full complexity of migration experiences. The lack of data on migration motivations, pre-migration resources, and reception conditions in host countries prevents us from fully understanding how the variations in multimorbidity patterns in these contexts. Additionally, while our study examines multimorbidity patterns by origin and receiving country groups separately, we could not conduct analyses that simultaneously stratify by both dimensions due to sample size limitations. Consequently, our analysis is limited in revealing the interaction between origin and destination on health, such as, for instance, different dietary adaptations of Asian immigrants in Mediterranean countries versus those in Northern Europe. We also have to rely on broad categories for the country of origin due to the sample size, such as the grouping of Southern and Eastern Asia under “Asia and Oceania.” Future research would benefit from employing larger and more comprehensive datasets, taking variations in migration experiences, origin–destination interactions, and heterogeneity across origin subgroups into account.

## Conclusion

To the best of our knowledge, this is the first study to describe multimorbidity patterns among native-born and immigrant chronic disease patients in Europe and to compare the magnitude of disparities in these patterns between subgroups by gender, country of origin, and receiving country. Our results indicate that the immigrants have a higher risk of multimorbidity for certain disease combinations, especially for those including stomach ulcers. We also find that the disadvantage in multimorbidity becomes more pronounced among women, immigrants from Asia or Eastern Europe, and those living in Northern Europe. Our descriptive findings on the overall and subgroup inequalities in multimorbidity between immigrants and native-born individuals can help identify which populations and health conditions should be prioritized in efforts to reduce health disparities between native-born and immigrant older adults in Europe and other aging societies.

## Supplementary Information

Below is the link to the electronic supplementary material.

Supplementary file1

## Data Availability

No datasets were generated or analysed during the current study.
