# Supplementary material for: Inequalities in multimorbidity between native-born and immigrant older adults across Europe
Source: Eur J Ageing. 2025 Aug 14;22(1):43. doi: 10.1007/s10433-025-00879-5 (PMC12354665; doi:10.1007/s10433-025-00879-5)
Supplement: Supplementary file 1 — Supplementary file1 [file 10433_2025_879_MOESM1_ESM.docx]

**Online supplementary material to “Inequalities in Multimorbidity between Native-born and Immigrant Older Adults across Europe”**

[Section I: Variable Description 3](#_Toc202430803)

[**Table S1.** Definitions and categorization of study variables 3](#_Toc202430804)

[Section II: Distribution of Chronic Diseases 5](#_Toc202430805)

[**Table S2.** Number and percentage of individuals with eight chronic diseases by gender, immigrant status, region of origin, and region of residence 5](#_Toc202430806)

[Section III: Detailed Findings from the Main Analysis 7](#_Toc202430807)

[**Table S3.** Prevalence of eight chronic diseases and multimorbidity by gender and immigrant status 7](#_Toc202430808)

[**Table S4.** Prevalence and relative risk of chronic disease combinations for immigrants versus native-born individuals by gender 8](#_Toc202430809)

[**Table S5.** Prevalence and relative risk of chronic disease combinations for immigrants versus native-born individuals by gender and age group 9](#_Toc202430810)

[**Table S6.** Prevalence and relative risk of chronic disease combinations for immigrants versus native-born individuals by gender and origin country group 12](#_Toc202430811)

[**Table S7.** Prevalence and relative risk of chronic disease combinations for immigrants versus native-born individuals by gender and region of residence group 17](#_Toc202430812)

[Section IV: Supplemental Analysis on Adjusted Risk Ratios 21](#_Toc202430813)

[**Table S8.** Adjusted risk ratios for multimorbidity that includes each chronic disease among men 21](#_Toc202430814)

[**Table S9.** Adjusted risk ratios for multimorbidity including each chronic disease among women 23](#_Toc202430815)

[**Fig. S1** Relative risks of multimorbidity among immigrants versus native-born individuals by gender based on Poisson regression models 25](#_Toc202430816)

[Section V: Supplemental Analysis Excluding Pre-Migration Conditions 26](#_Toc202430817)

[**Table S10.** Number and percentage of pre- and post-migration onsets among immigrants with eight chronic diseases by gender, region of origin, and region of residence 26](#_Toc202430818)

[**Fig. S2** Relative risks of multimorbidity among immigrants versus native-born individuals by gender and region of origin based on conditions developed post-migration 28](#_Toc202430819)

[**Fig. S3** Relative risks of multimorbidity among immigrants versus native-born individuals by gender and region of residence region based on conditions developed post-migration 29](#_Toc202430820)

[Section VI: Supplemental Analysis Including Individuals Aged 80 and Above 30](#_Toc202430821)

[**Table S11.** Number and percentage of individuals with eight chronic diseases among samples aged 50–79 years versus those aged 80 years and above by gender 30](#_Toc202430822)

[**Fig. S4** Relative risks of multimorbidity among immigrants versus native-born individuals by gender in samples including aged 80 and above 31](#_Toc202430823)

[Section VII: Supplemental Analysis on Balanced Panel 32](#_Toc202430824)

[**Fig. S5** Relative risks of multimorbidity among immigrants versus native-born individuals by gender in Waves 7 onward 32](#_Toc202430825)

# Section I: Variable Description

## **Table S1.** Definitions and categorization of study variables

| **Variable** | **Description** |
| --- | --- |
| **Region of origin** |  |
| Africa | Africa, Algeria, Angola, Benin, Burkina Faso, Burundi, Cameroon, Cape Verde, Central African Republic, Chad, Comoros, Congo (both), Democratic Republic of Congo (Zaire), Republic of Congo, Cote d'Ivoire, Egypt, Equatorial Guinea, Eritrea, Ethiopia, Ethiopia (before Eritrea broke away), Former Protectorate of Northern Rhodesia, Gabon, Gambia, Ghana, Guinea, Guinea-Bissau, Kenya, Liberia, Libyan Arab Jamahiriya, Madagascar, Mali, Mauritania, Mauritius, Morocco, Mozambique, Nigeria, Reunion, Rwanda, Sao Tome and Principe, Senegal, Sierra Leone, Somalia, South Africa, Sudan, United Republic of Tanzania, Togo, Tunisia, Zambia, Zimbabwe |
| Asia & Oceania |  |
| Asia | Afghanistan, Afghan-Turkish, Armenia, Azerbaijan, Bangladesh, Bhutan, Borneo Island, Cambodia, China, Former Netherlands East-Indies, Georgia, Hong Kong, India, Indonesia, Islamic Republic of Iran, Iraq, Israel, Japan, Jordan, Kazakhstan, Korea (North or South), Democratic People's Republic of Korea, Republic of Korea, Kyrgyzstan, Lao People's Democratic Republic, Lebanon, Macau, Malaysia, Minor Asia, Pakistan, Palestinian Territory, Philippines, Singapore, Sri Lanka, Syrian Arab Republic, Taiwan, Tajikistan, Thailand, Turkey, Turkish-Kurdish, Turkmenistan, Uzbekistan, Viet Nam |
| Oceania | Australia, French Polynesia, New Zealand |
| Latin America and the Caribbean | Argentina, Aruba, Bolivia, Brazil, Chile, Colombia, Costa Rica, Cuba, Curaçao, Dominican Republic, Ecuador, El Salvador, French Guiana, Grenada, Guadeloupe, Guyana, Haiti, Honduras, Jamaica, Martinique, Mexico, Netherlands Antilles, Paraguay, Peru, South America, Suriname, Uruguay, Venezuela, Virgin Islands (U.S.) |
| Eastern Europe | Armenia, Azerbaijan, Belarus, Bulgaria, Chechnya, Czech Republic, Czechoslovakia, Estonia, Former Eastern Territory of German Reich, Georgia, Hungary, Kazakhstan, Kyrgyzstan, Latvia, Lithuania, Moldova, Republic of, Poland, Romania, Russian Federation, Slovakia, Tajikistan, Turkmenistan, Ukraine, Union of Soviet Socialist Republics, Uzbekistan |
| Other Europe & North America |  |
| Other Europe | Albania, Austria, Belgium, Bosnia and Herzegovina, Croatia, Cyprus, Denmark, Faroe Islands, Finland, Former Territories of German Reich, France, German Spanish, Germany, Greece, Iceland, Ireland, Italy, Kosovo, Liechtenstein, Luxembourg, Macedonia (former Yugoslav Republic of), Malta, Monaco, Montenegro, Netherlands, Norway, Portugal, Serbia, Slovenia, Socialist Federal Republic of Yugoslavia, Spain, Sweden, Switzerland, United Kingdom |
| North America | Canada, Greenland, United States of America |
| **Region of residence** |  |
| Northern Europe | Denmark, Finland, Ireland, Sweden |
| Western Europe | Austria, Belgium, France, Germany, Luxembourg, Netherlands, Switzerland |
| Southern Europe | Croatia, Cyprus, Greece, Italy, Malta, Portugal, Slovenia, Spain |
| Eastern Europe | Bulgaria, Czech Republic, Estonia, Hungary, Latvia, Lithuania, Poland, Romania, Slovakia |
| **Chronic disease groups** |  |
| Cancer | Ever diagnosed with cancer or malignant tumor |
| Cardiovascular diseases |  |
| Heart attack | Ever diagnosed with heart attack (including myocardial infarction, coronary thrombosis, or any other heart problem including congestive heart failure) or currently taking medication for coronary or cerebrovascular diseases or other heart diseases |
| Hypertension | Ever diagnosed with high blood pressure or hypertension or currently taking medication for high blood pressure |
| Stroke | Ever diagnosed with stroke or cerebral vascular disease or currently taking medication for coronary or cerebrovascular diseases |
| Diabetes | Ever diagnosed with diabetes or high blood sugar or currently taking medication for diabetes |
| Dementia & Parkinson’s disease | Ever diagnosed with Alzheimer's disease, dementia, organic brain syndrome, senility, or any other serious memory impairment or Parkinson’s disease |
| Mental disorders | Ever diagnosed with affective or emotional disorders including anxiety, nervous, or psychiatric problems, ever treated for depression, ever admitted to hospital for depression, or currently taking medication for sleep problems, anxiety, and/or depression |
| Musculoskeletal diseases |  |
| Arthritis | Ever diagnosed with arthritis, rheumatoid arthritis, osteoarthritis, or other rheumatism |
| Osteoporosis | Ever diagnosed with osteoporosis or currently taking medication for osteoporosis, including hormonal and non-hormonal drugs |
| Hip fracture | Ever diagnosed with hip fracture or femoral fracture |
| Respiratory diseases |  |
| Chronic lung disease | Ever diagnosed with chronic lung disease such as chronic bronchitis or emphysema or currently taking medication for chronic bronchitis |
| Asthma | Ever diagnosed with asthma or currently taking medication for asthma |
| Stomach ulcer | Ever diagnosed with stomach ulcer, duodenal ulcer, and/or peptic ulcer |
| **Covariates for supplemental analysis** |  |
| Education | Low (lower secondary education or less), medium (upper secondary or post-secondary non-tertiary education), and high (tertiary education) levels of education based on International Standard Classification of Education (ISCED) 1997 |
| Household income | Low, medium, and high levels based on tertile cutoffs of imputed household net income in each country |
| Employment status | Working versus not working status |
| Marital status | Married versus not married |
| Country | Austria, Belgium, Bulgaria, Croatia, Czech Republic, Denmark, Estonia, Finland, France, Germany, Greece, Hungary, Ireland, Italy, Latvia, Lithuania, Luxembourg, Malta, Netherlands, Poland, Portugal, Romania, Slovakia, Slovenia, Spain, Sweden, Switzerland |
| Survey wave | Wave 2, 4, 5, 6, 7, 8, 9 |

# Section II: Distribution of Chronic Diseases

## **Table S2.** Number and percentage of individuals with eight chronic diseases by gender, immigrant status, region of origin, and region of residence

|  | **Men** | | **Women** | |
| --- | --- | --- | --- | --- |
|  | **Native-born** | **Immigrant** | **Native-born** | **Immigrant** |
| **Total population** |  |  |  |  |
| Cardiovascular | 83,463 (58.7%) | 7,437 (58.2%) | 98,282 (55.1%) | 9,940 (58.7%) |
| Musculoskeletal | 38,982 (27.4%) | 3,734 (29.2%) | 80,462 (45.1%) | 8,161 (48.2%) |
| Mental disorder | 23,067 (16.2%) | 2,210 (17.3%) | 52,593 (29.5%) | 5,352 (31.6%) |
| Diabetes | 24,142 (17.0%) | 2,431 (19.0%) | 24,383 (13.7%) | 2,876 (17.0%) |
| Respiratory | 14,654 (10.3%) | 1,476 (11.5%) | 18,117 (10.1%) | 2,130 (12.6%) |
| Cancer | 10,591 ( 7.4%) | 949 ( 7.4%) | 15,403 ( 8.6%) | 1,508 ( 8.9%) |
| Stomach ulcer | 11,401 ( 8.0%) | 1,356 (10.6%) | 13,725 ( 7.7%) | 1,955 (11.5%) |
| Dementia & Parkinson's | 3,294 ( 2.3%) | 314 ( 2.5%) | 3,698 ( 2.1%) | 366 ( 2.2%) |
| **Age group: 50-59** |  |  |  |  |
| Cardiovascular | 14,705 (39.3%) | 1,414 (37.6%) | 18,241 (34.9%) | 2,096 (38.9%) |
| Musculoskeletal | 6,486 (17.3%) | 751 (20.0%) | 15,027 (28.7%) | 1,730 (32.1%) |
| Mental disorder | 5,083 (13.6%) | 602 (16.0%) | 12,599 (24.1%) | 1,449 (26.9%) |
| Diabetes | 3,469 ( 9.3%) | 474 (12.6%) | 3,634 ( 6.9%) | 520 ( 9.7%) |
| Respiratory | 2,479 ( 6.6%) | 273 ( 7.3%) | 3,735 ( 7.1%) | 520 ( 9.7%) |
| Cancer | 1,188 ( 3.2%) | 112 ( 3.0%) | 2,918 ( 5.6%) | 334 ( 6.2%) |
| Stomach ulcer | 2,343 ( 6.3%) | 357 ( 9.5%) | 2,878 ( 5.5%) | 481 ( 8.9%) |
| Dementia & Parkinson's | 276 ( 0.7%) | 31 ( 0.8%) | 315 ( 0.6%) | 42 ( 0.8%) |
| **Age group: 60-69** |  |  |  |  |
| Cardiovascular | 35,135 (58.9%) | 3,017 (60.8%) | 39,572 (55.3%) | 3,788 (59.9%) |
| Musculoskeletal | 16,171 (27.1%) | 1,512 (30.5%) | 32,792 (45.9%) | 3,197 (50.6%) |
| Mental disorder | 9,592 (16.1%) | 890 (17.9%) | 21,164 (29.6%) | 2,034 (32.2%) |
| Diabetes | 10,279 (17.2%) | 994 (20.0%) | 9,654 (13.5%) | 1,106 (17.5%) |
| Respiratory | 5,943 (10.0%) | 600 (12.1%) | 7,175 (10.0%) | 831 (13.2%) |
| Cancer | 4,008 ( 6.7%) | 350 ( 7.1%) | 6,289 ( 8.8%) | 580 ( 9.2%) |
| Stomach ulcer | 4,739 ( 7.9%) | 503 (10.1%) | 5,614 ( 7.9%) | 726 (11.5%) |
| Dementia & Parkinson's | 996 ( 1.7%) | 89 ( 1.8%) | 1,063 ( 1.5%) | 110 ( 1.7%) |
| **Age group: 70-79** |  |  |  |  |
| Cardiovascular | 33,623 (74.4%) | 3,006 (74.1%) | 40,469 (74.0%) | 4,056 (77.6%) |
| Musculoskeletal | 16,325 (36.1%) | 1,471 (36.3%) | 32,643 (59.7%) | 3,234 (61.9%) |
| Mental disorder | 8,392 (18.6%) | 718 (17.7%) | 18,830 (34.4%) | 1,869 (35.8%) |
| Diabetes | 10,394 (23.0%) | 963 (23.7%) | 11,095 (20.3%) | 1,250 (23.9%) |
| Respiratory | 6,232 (13.8%) | 603 (14.9%) | 7,207 (13.2%) | 779 (14.9%) |
| Cancer | 5,395 (11.9%) | 487 (12.0%) | 6,196 (11.3%) | 594 (11.4%) |
| Stomach ulcer | 4,319 ( 9.6%) | 496 (12.2%) | 5,233 ( 9.6%) | 748 (14.3%) |
| Dementia & Parkinson's | 2,022 ( 4.5%) | 194 ( 4.8%) | 2,320 ( 4.2%) | 214 ( 4.1%) |
| **Region of origin: Africa** |  |  |  |  |
| Cardiovascular | - (-) | 605 (51.8%) | - (-) | 588 (47.2%) |
| Musculoskeletal | - (-) | 326 (27.9%) | - (-) | 615 (49.4%) |
| Mental disorder | - (-) | 203 (17.4%) | - (-) | 470 (37.7%) |
| Diabetes | - (-) | 277 (23.7%) | - (-) | 239 (19.2%) |
| Respiratory | - (-) | 153 (13.1%) | - (-) | 124 (10.0%) |
| Cancer | - (-) | 67 ( 5.7%) | - (-) | 103 ( 8.3%) |
| Stomach ulcer | - (-) | 92 ( 7.9%) | - (-) | 119 ( 9.6%) |
| Dementia & Parkinson's | - (-) | 11 ( 0.9%) | - (-) | 21 ( 1.7%) |
| **Region of origin: Asia & Oceania** |  |  |  |  |
| Cardiovascular | - (-) | 390 (46.1%) | - (-) | 495 (51.7%) |
| Musculoskeletal | - (-) | 211 (24.9%) | - (-) | 398 (41.6%) |
| Mental disorder | - (-) | 142 (16.8%) | - (-) | 247 (25.8%) |
| Diabetes | - (-) | 158 (18.7%) | - (-) | 189 (19.7%) |
| Respiratory | - (-) | 96 (11.3%) | - (-) | 104 (10.9%) |
| Cancer | - (-) | 24 ( 2.8%) | - (-) | 65 ( 6.8%) |
| Stomach ulcer | - (-) | 85 (10.0%) | - (-) | 126 (13.2%) |
| Dementia & Parkinson's | - (-) | 14 ( 1.7%) | - (-) | 19 ( 2.0%) |
| **Region of origin: Latin America and the Caribbean** |  |  |  |  |
| Cardiovascular | - (-) | 161 (50.6%) | - (-) | 214 (41.9%) |
| Musculoskeletal | - (-) | 68 (21.4%) | - (-) | 201 (39.3%) |
| Mental disorder | - (-) | 67 (21.1%) | - (-) | 148 (29.0%) |
| Diabetes | - (-) | 63 (19.8%) | - (-) | 56 (11.0%) |
| Respiratory | - (-) | 22 ( 6.9%) | - (-) | 40 ( 7.8%) |
| Cancer | - (-) | 32 (10.1%) | - (-) | 36 ( 7.0%) |
| Stomach ulcer | - (-) | 12 ( 3.8%) | - (-) | 37 ( 7.2%) |
| Dementia & Parkinson's | - (-) | 7 ( 2.2%) | - (-) | 7 ( 1.4%) |
| **Region of origin: Eastern Europe** |  |  |  |  |
| Cardiovascular | - (-) | 2,601 (66.0%) | - (-) | 4,243 (68.9%) |
| Musculoskeletal | - (-) | 1,304 (33.1%) | - (-) | 3,123 (50.7%) |
| Mental disorder | - (-) | 614 (15.6%) | - (-) | 1,945 (31.6%) |
| Diabetes | - (-) | 666 (16.9%) | - (-) | 1,340 (21.8%) |
| Respiratory | - (-) | 457 (11.6%) | - (-) | 896 (14.6%) |
| Cancer | - (-) | 274 ( 7.0%) | - (-) | 523 ( 8.5%) |
| Stomach ulcer | - (-) | 597 (15.1%) | - (-) | 890 (14.5%) |
| Dementia & Parkinson's | - (-) | 104 ( 2.6%) | - (-) | 156 ( 2.5%) |
| **Region of origin: Other Europe & North America** |  |  |  |  |
| Cardiovascular | - (-) | 3,680 (56.6%) | - (-) | 4,400 (54.6%) |
| Musculoskeletal | - (-) | 1,825 (28.0%) | - (-) | 3,824 (47.4%) |
| Mental disorder | - (-) | 1,184 (18.2%) | - (-) | 2,542 (31.5%) |
| Diabetes | - (-) | 1,267 (19.5%) | - (-) | 1,052 (13.1%) |
| Respiratory | - (-) | 748 (11.5%) | - (-) | 966 (12.0%) |
| Cancer | - (-) | 552 ( 8.5%) | - (-) | 781 ( 9.7%) |
| Stomach ulcer | - (-) | 570 ( 8.8%) | - (-) | 783 ( 9.7%) |
| Dementia & Parkinson's | - (-) | 178 ( 2.7%) | - (-) | 163 ( 2.0%) |
| **Region of residence: Northern Europe** |  |  |  |  |
| Cardiovascular | 9,521 (53.5%) | 479 (51.7%) | 9,570 (46.9%) | 670 (52.2%) |
| Musculoskeletal | 5,014 (28.2%) | 234 (25.3%) | 8,796 (43.1%) | 559 (43.6%) |
| Mental disorder | 2,494 (14.0%) | 165 (17.8%) | 4,837 (23.7%) | 368 (28.7%) |
| Diabetes | 2,329 (13.1%) | 150 (16.2%) | 1,839 ( 9.0%) | 120 ( 9.4%) |
| Respiratory | 1,862 (10.5%) | 81 ( 8.7%) | 2,402 (11.8%) | 203 (15.8%) |
| Cancer | 1,564 ( 8.8%) | 77 ( 8.3%) | 2,257 (11.1%) | 123 ( 9.6%) |
| Stomach ulcer | 974 ( 5.5%) | 79 ( 8.5%) | 1,020 ( 5.0%) | 91 ( 7.1%) |
| Dementia & Parkinson's | 324 ( 1.8%) | 38 ( 4.1%) | 267 ( 1.3%) | 20 ( 1.6%) |
| **Region of residence: Western Europe** |  |  |  |  |
| Cardiovascular | 27,333 (56.1%) | 3,437 (55.6%) | 28,735 (49.0%) | 3,934 (50.9%) |
| Musculoskeletal | 14,382 (29.5%) | 1,956 (31.7%) | 27,764 (47.3%) | 3,880 (50.2%) |
| Mental disorder | 9,847 (20.2%) | 1,173 (19.0%) | 20,336 (34.7%) | 2,661 (34.4%) |
| Diabetes | 7,502 (15.4%) | 1,241 (20.1%) | 6,338 (10.8%) | 1,139 (14.7%) |
| Respiratory | 5,477 (11.2%) | 753 (12.2%) | 6,539 (11.2%) | 964 (12.5%) |
| Cancer | 4,310 ( 8.9%) | 496 ( 8.0%) | 5,810 ( 9.9%) | 762 ( 9.9%) |
| Stomach ulcer | 3,591 ( 7.4%) | 542 ( 8.8%) | 4,144 ( 7.1%) | 765 ( 9.9%) |
| Dementia & Parkinson's | 1,139 ( 2.3%) | 112 ( 1.8%) | 1,043 ( 1.8%) | 163 ( 2.1%) |
| **Region of residence: Southern Europe** |  |  |  |  |
| Cardiovascular | 24,544 (60.7%) | 1,466 (57.5%) | 28,450 (56.5%) | 1,754 (56.5%) |
| Musculoskeletal | 9,029 (22.3%) | 429 (16.8%) | 22,859 (45.4%) | 1,150 (37.1%) |
| Mental disorder | 6,059 (15.0%) | 393 (15.4%) | 14,815 (29.4%) | 776 (25.0%) |
| Diabetes | 7,948 (19.7%) | 522 (20.5%) | 7,827 (15.5%) | 453 (14.6%) |
| Respiratory | 3,892 ( 9.6%) | 230 ( 9.0%) | 4,140 ( 8.2%) | 268 ( 8.6%) |
| Cancer | 2,433 ( 6.0%) | 184 ( 7.2%) | 3,721 ( 7.4%) | 224 ( 7.2%) |
| Stomach ulcer | 3,385 ( 8.4%) | 211 ( 8.3%) | 4,185 ( 8.3%) | 283 ( 9.1%) |
| Dementia & Parkinson's | 969 ( 2.4%) | 86 ( 3.4%) | 1,228 ( 2.4%) | 65 ( 2.1%) |
| **Region of residence: Eastern Europe** |  |  |  |  |
| Cardiovascular | 22,065 (62.4%) | 2,055 (65.7%) | 31,527 (64.2%) | 3,582 (74.5%) |
| Musculoskeletal | 10,557 (29.8%) | 1,115 (35.7%) | 21,043 (42.9%) | 2,572 (53.5%) |
| Mental disorder | 4,667 (13.2%) | 479 (15.3%) | 12,605 (25.7%) | 1,547 (32.2%) |
| Diabetes | 6,363 (18.0%) | 518 (16.6%) | 8,379 (17.1%) | 1,164 (24.2%) |
| Respiratory | 3,423 ( 9.7%) | 412 (13.2%) | 5,036 (10.3%) | 695 (14.5%) |
| Cancer | 2,284 ( 6.5%) | 192 ( 6.1%) | 3,615 ( 7.4%) | 399 ( 8.3%) |
| Stomach ulcer | 3,451 ( 9.8%) | 524 (16.8%) | 4,376 ( 8.9%) | 816 (17.0%) |
| Dementia & Parkinson's | 862 ( 2.4%) | 78 ( 2.5%) | 1,160 ( 2.4%) | 118 ( 2.5%) |

# Section III: Detailed Findings from the Main Analysis

## **Table S3.** Prevalence of eight chronic diseases and multimorbidity by gender and immigrant status

|  | **Men** | | **Women** | |
| --- | --- | --- | --- | --- |
|  | **Native-born** | **Immigrant** | **Native-born** | **Immigrant** |
| Prevalence of cardiovascular diseases | 55.2% (54.9–55.5) | 55.1% (54.3–56.0) | 52.2% (51.9–52.4) | 56.3% (55.5–57.0) |
| Prevalence of musculoskeletal diseases | 25.6% (25.4–25.8) | 27.8% (27.1–28.6) | 42.7% (42.5–43.0) | 46.3% (45.6–47.0) |
| Prevalence of mental disorder | 15.7% (15.6–15.9) | 17.1% (16.5–17.8) | 28.7% (28.5–28.9) | 31.0% (30.4–31.7) |
| Prevalence of diabetes | 15.6% (15.4–15.8) | 18.1% (17.4–18.7) | 12.7% (12.6–12.9) | 16.1% (15.5–16.6) |
| Prevalence of respiratory diseases | 9.6% ( 9.5– 9.8) | 10.9% (10.4–11.4) | 9.7% ( 9.6– 9.9) | 12.2% (11.7–12.7) |
| Prevalence of cancer | 6.7% ( 6.6– 6.8) | 6.7% ( 6.3– 7.2) | 8.2% ( 8.1– 8.3) | 8.6% ( 8.1– 9.0) |
| Prevalence of stomach ulcer | 7.7% ( 7.6– 7.8) | 10.4% ( 9.9–10.9) | 7.4% ( 7.3– 7.5) | 11.2% (10.8–11.7) |
| Prevalence of dementia | 2.0% ( 2.0– 2.1) | 2.2% ( 2.0– 2.5) | 1.9% ( 1.8– 1.9) | 2.0% ( 1.8– 2.2) |
| Prevalence of multimorbidity |  |  |  |  |
| Among the total sample | 39.5% (39.3–39.8) | 43.2% (42.3–44.0) | 48.2% (48.0–48.5) | 53.8% (53.2–54.6) |
| Among those with cardiovascular diseases | 60.1% (59.8–60.5) | 65.2% (64.0–66.3) | 71.8% (71.5–72.1) | 76.1% (75.3–76.9) |
| Among those with musculoskeletal diseases | 79.1% (78.7–79.5) | 79.5% (78.2–80.8) | 79.1% (78.8–79.4) | 82.5% (81.7–83.3) |
| Among those with mental disorder | 83.9% (83.5–84.5) | 86.1% (84.7–87.5) | 84.6% (84.2–84.9) | 87.8% (86.9–88.7) |
| Among those with diabetes | 87.4% (87.0–87.8) | 87.3% (86.0–88.6) | 92.6% (92.2–92.9) | 94.1% (93.2–94.9) |
| Among those with respiratory diseases | 84.3% (83.6–84.8) | 87.7% (86.0–89.4) | 89.9% (89.4–90.3) | 94.0% (92.9–95.0) |
| Among those with cancer | 80.0% (79.3–80.8) | 80.6% (78.3–83.0) | 82.7% (82.1–83.3) | 87.6% (86.0–89.2) |
| Among those with stomach ulcer | 83.9% (83.2–84.6) | 84.1% (82.2–85.9) | 90.4% (90.0–90.9) | 94.2% (93.1–95.1) |
| Among those with dementia | 90.6% (89.6–91.6) | 91.5% (88.5–94.3) | 94.8% (94.1–95.5) | 95.4% (93.2–97.5) |

*Notes:* Values are age-standardized using the 2013 European standard population

## **Table S4.** Prevalence and relative risk of chronic disease combinations for immigrants versus native-born individuals by gender

|  | **Men** | | | **Women** | | |
| --- | --- | --- | --- | --- | --- | --- |
|  | **Native-born** | **Immigrant** | **RR (95% CI)** | **Native-born** | **Immigrant** | **RR (95% CI)** |
| Cardiovascular |  |  |  |  |  |  |
| + Musculoskeletal | 29.9% | 32.4% | 1.08 (1.05–1.12) | 48.5% | 52.4% | 1.08 (1.06–1.10) |
| + Mental disorder | 19.0% | 21.4% | 1.13 (1.08–1.18) | 33.8% | 36.3% | 1.07 (1.05–1.10) |
| + Diabetes | 21.9% | 25.0% | 1.14 (1.10–1.19) | 19.0% | 23.4% | 1.23 (1.18–1.28) |
| + Respiratory | 11.7% | 13.6% | 1.16 (1.09–1.22) | 12.3% | 15.6% | 1.27 (1.21–1.33) |
| + Cancer | 7.4% | 7.4% | 1.00 (0.92–1.09) | 8.8% | 9.7% | 1.10 (1.03–1.17) |
| + Stomach ulcer | 9.1% | 11.6% | 1.28 (1.19–1.36) | 9.1% | 14.2% | 1.56 (1.49–1.64) |
| + Dementia | 2.5% | 2.5% | 1.02 (0.87–1.17) | 2.3% | 2.4% | 1.02 (0.89–1.16) |
| Musculoskeletal |  |  |  |  |  |  |
| + Cardiovascular | 64.6% | 63.9% | 0.99 (0.97–1.01) | 59.2% | 63.7% | 1.08 (1.06–1.10) |
| + Mental disorder | 25.5% | 25.8% | 1.01 (0.96–1.08) | 39.8% | 42.8% | 1.08 (1.05–1.10) |
| + Diabetes | 19.5% | 22.7% | 1.16 (1.09–1.23) | 15.4% | 19.6% | 1.28 (1.22–1.34) |
| + Respiratory | 15.7% | 15.9% | 1.01 (0.94–1.09) | 14.3% | 17.2% | 1.21 (1.14–1.27) |
| + Cancer | 8.1% | 7.7% | 0.94 (0.84–1.05) | 9.7% | 10.1% | 1.04 (0.97–1.12) |
| + Stomach ulcer | 12.6% | 16.0% | 1.27 (1.16–1.37) | 11.2% | 17.0% | 1.51 (1.43–1.59) |
| + Dementia | 3.0% | 2.7% | 0.90 (0.72–1.11) | 2.4% | 2.5% | 1.06 (0.91–1.21) |
| Mental disorder |  |  |  |  |  |  |
| + Cardiovascular | 65.9% | 67.3% | 1.02 (0.99–1.05) | 60.7% | 65.1% | 1.07 (1.05–1.09) |
| + Musculoskeletal | 40.7% | 41.8% | 1.03 (0.97–1.08) | 58.3% | 62.6% | 1.07 (1.05–1.10) |
| + Diabetes | 21.3% | 27.2% | 1.27 (1.18–1.37) | 17.0% | 22.0% | 1.30 (1.23–1.37) |
| + Respiratory | 18.1% | 19.6% | 1.08 (0.98–1.18) | 15.7% | 20.1% | 1.28 (1.21–1.35) |
| + Cancer | 10.0% | 9.7% | 0.96 (0.84–1.10) | 10.9% | 11.6% | 1.06 (0.98–1.15) |
| + Stomach ulcer | 15.6% | 20.0% | 1.28 (1.17–1.40) | 13.1% | 19.6% | 1.49 (1.41–1.58) |
| + Dementia | 6.5% | 6.7% | 1.03 (0.87–1.21) | 4.0% | 4.1% | 1.04 (0.89–1.17) |
| Diabetes |  |  |  |  |  |  |
| + Cardiovascular | 79.1% | 76.1% | 0.96 (0.94–0.98) | 80.1% | 83.4% | 1.04 (1.02–1.06) |
| + Musculoskeletal | 32.3% | 34.9% | 1.08 (1.02–1.14) | 52.6% | 57.0% | 1.08 (1.05–1.12) |
| + Mental disorder | 21.9% | 26.3% | 1.20 (1.12–1.28) | 39.6% | 43.3% | 1.09 (1.04–1.14) |
| + Respiratory | 13.7% | 14.3% | 1.04 (0.93–1.15) | 16.0% | 19.3% | 1.20 (1.11–1.30) |
| + Cancer | 7.9% | 7.9% | 1.01 (0.88–1.16) | 9.8% | 10.4% | 1.06 (0.96–1.18) |
| + Stomach ulcer | 9.3% | 12.9% | 1.38 (1.23–1.53) | 10.7% | 16.7% | 1.56 (1.42–1.71) |
| + Dementia | 2.9% | 3.9% | 1.35 (1.09–1.64) | 3.1% | 3.1% | 1.00 (0.78–1.21) |
| Respiratory |  |  |  |  |  |  |
| + Cardiovascular | 67.1% | 68.7% | 1.02 (0.99–1.06) | 65.4% | 70.6% | 1.08 (1.05–1.11) |
| + Musculoskeletal | 41.7% | 41.0% | 0.98 (0.92–1.05) | 62.3% | 64.7% | 1.04 (1.00–1.07) |
| + Mental disorder | 30.2% | 32.2% | 1.06 (0.98–1.15) | 47.0% | 51.7% | 1.10 (1.05–1.15) |
| + Diabetes | 21.9% | 23.8% | 1.08 (0.98–1.18) | 20.2% | 24.3% | 1.20 (1.11–1.30) |
| + Cancer | 10.0% | 12.2% | 1.22 (1.05–1.41) | 11.2% | 13.7% | 1.23 (1.10–1.38) |
| + Stomach ulcer | 15.4% | 16.9% | 1.09 (0.97–1.23) | 14.3% | 21.6% | 1.51 (1.38–1.65) |
| + Dementia | 3.9% | 3.0% | 0.77 (0.55–1.01) | 3.4% | 3.6% | 1.06 (0.82–1.34) |
| Cancer |  |  |  |  |  |  |
| + Cardiovascular | 62.0% | 60.4% | 0.97 (0.92–1.03) | 56.0% | 63.3% | 1.13 (1.08–1.18) |
| + Musculoskeletal | 31.3% | 32.3% | 1.03 (0.93–1.12) | 50.4% | 54.2% | 1.08 (1.03–1.13) |
| + Mental disorder | 24.7% | 25.6% | 1.04 (0.92–1.17) | 38.7% | 42.3% | 1.09 (1.02–1.17) |
| + Diabetes | 18.7% | 21.5% | 1.15 (1.00–1.28) | 15.1% | 19.3% | 1.28 (1.16–1.43) |
| + Respiratory | 15.0% | 20.3% | 1.35 (1.17–1.53) | 13.2% | 19.6% | 1.48 (1.32–1.64) |
| + Stomach ulcer | 11.6% | 16.4% | 1.41 (1.20–1.62) | 11.0% | 16.7% | 1.52 (1.35–1.71) |
| + Dementia | 3.0% | 2.9% | 0.97 (0.62–1.35) | 2.3% | 2.6% | 1.13 (0.77–1.50) |
| Stomach ulcer |  |  |  |  |  |  |
| + Cardiovascular | 64.9% | 61.2% | 0.94 (0.90–0.98) | 63.9% | 70.6% | 1.11 (1.07–1.14) |
| + Musculoskeletal | 41.2% | 42.3% | 1.03 (0.96–1.09) | 64.5% | 69.4% | 1.08 (1.04–1.11) |
| + Mental disorder | 32.0% | 32.8% | 1.03 (0.95–1.11) | 51.4% | 54.8% | 1.07 (1.02–1.12) |
| + Diabetes | 18.5% | 22.0% | 1.19 (1.06–1.33) | 18.0% | 23.5% | 1.31 (1.20–1.42) |
| + Respiratory | 19.1% | 17.7% | 0.93 (0.82–1.04) | 18.8% | 23.5% | 1.25 (1.15–1.36) |
| + Cancer | 9.9% | 10.7% | 1.08 (0.91–1.26) | 12.1% | 12.7% | 1.05 (0.92–1.18) |
| + Dementia | 3.7% | 3.9% | 1.06 (0.77–1.36) | 3.3% | 3.0% | 0.92 (0.68–1.18) |
| Dementia |  |  |  |  |  |  |
| + Cardiovascular | 70.4% | 61.9% | 0.88 (0.80–0.96) | 70.9% | 73.7% | 1.04 (0.97–1.10) |
| + Musculoskeletal | 40.5% | 37.4% | 0.92 (0.79–1.07) | 58.8% | 61.7% | 1.05 (0.96–1.14) |
| + Mental disorder | 58.3% | 63.2% | 1.09 (0.99–1.18) | 69.2% | 70.4% | 1.02 (0.95–1.09) |
| + Diabetes | 23.0% | 36.3% | 1.58 (1.32–1.84) | 22.6% | 25.9% | 1.15 (0.94–1.37) |
| + Respiratory | 20.1% | 15.9% | 0.79 (0.59–1.00) | 20.4% | 24.5% | 1.20 (0.98–1.42) |
| + Cancer | 10.2% | 8.7% | 0.85 (0.55–1.18) | 10.8% | 11.3% | 1.05 (0.76–1.39) |
| + Stomach ulcer | 15.3% | 22.2% | 1.45 (1.13–1.79) | 15.0% | 18.2% | 1.21 (0.94–1.48) |

*Notes:* Values are age-standardized using the 2013 European standard population.

## **Table S5.** Prevalence and relative risk of chronic disease combinations for immigrants versus native-born individuals by gender and age group

|  | **Men** | | | **Women** | | |
| --- | --- | --- | --- | --- | --- | --- |
|  | **Native-born** | **Immigrant** | **RR (95% CI)** | **Native-born** | **Immigrant** | **RR (95% CI)** |
| **50–59** |  |  |  |  |  |  |
| Cardiovascular |  |  |  |  |  |  |
| + Musculoskeletal | 22.6% | 26.1% | 1.15 (1.05–1.27) | 36.9% | 41.0% | 1.11 (1.05–1.17) |
| + Mental disorder | 18.0% | 22.6% | 1.26 (1.13–1.39) | 31.4% | 34.5% | 1.10 (1.03–1.17) |
| + Diabetes | 16.8% | 22.3% | 1.33 (1.19–1.47) | 13.9% | 18.6% | 1.33 (1.21–1.47) |
| + Respiratory | 9.2% | 11.0% | 1.19 (1.01–1.37) | 10.6% | 14.5% | 1.37 (1.22–1.53) |
| + Cancer | 4.0% | 3.3% | 0.81 (0.59–1.03) | 6.4% | 7.9% | 1.24 (1.05–1.44) |
| + Stomach ulcer | 8.4% | 10.9% | 1.30 (1.11–1.53) | 7.7% | 13.2% | 1.71 (1.50–1.90) |
| + Dementia | 1.1% | 0.8% | 0.71 (0.32–1.19) | 1.0% | 1.4% | 1.37 (0.89–1.96) |
| Musculoskeletal |  |  |  |  |  |  |
| + Cardiovascular | 51.3% | 49.1% | 0.96 (0.88–1.03) | 44.9% | 49.7% | 1.11 (1.05–1.16) |
| + Mental disorder | 25.0% | 24.8% | 0.99 (0.86–1.13) | 38.0% | 42.5% | 1.12 (1.05–1.18) |
| + Diabetes | 13.8% | 16.6% | 1.20 (1.01–1.43) | 10.6% | 14.1% | 1.34 (1.18–1.50) |
| + Respiratory | 13.3% | 13.4% | 1.01 (0.82–1.21) | 12.9% | 15.0% | 1.17 (1.03–1.31) |
| + Cancer | 4.1% | 3.9% | 0.95 (0.62–1.35) | 7.4% | 7.6% | 1.04 (0.85–1.24) |
| + Stomach ulcer | 12.1% | 15.6% | 1.29 (1.08–1.54) | 10.1% | 15.4% | 1.53 (1.35–1.73) |
| + Dementia | 1.5% | 1.6% | 1.09 (0.55–1.89) | 1.1% | 1.3% | 1.17 (0.69–1.72) |
| Mental disorder |  |  |  |  |  |  |
| + Cardiovascular | 51.9% | 53.2% | 1.02 (0.94–1.11) | 45.5% | 49.9% | 1.10 (1.03–1.15) |
| + Musculoskeletal | 31.9% | 30.9% | 0.97 (0.85–1.09) | 45.3% | 50.7% | 1.12 (1.06–1.18) |
| + Diabetes | 14.4% | 22.1% | 1.53 (1.30–1.79) | 11.2% | 14.8% | 1.33 (1.15–1.51) |
| + Respiratory | 14.8% | 16.6% | 1.12 (0.92–1.34) | 13.5% | 19.5% | 1.45 (1.29–1.60) |
| + Cancer | 5.7% | 5.0% | 0.87 (0.58–1.21) | 8.3% | 9.0% | 1.09 (0.91–1.27) |
| + Stomach ulcer | 14.6% | 18.1% | 1.24 (1.04–1.48) | 10.8% | 18.8% | 1.74 (1.54–1.95) |
| + Dementia | 3.7% | 4.0% | 1.09 (0.66–1.60) | 1.8% | 2.1% | 1.15 (0.78–1.64) |
| Diabetes |  |  |  |  |  |  |
| + Cardiovascular | 71.2% | 66.5% | 0.93 (0.87–0.99) | 69.8% | 74.8% | 1.07 (1.02–1.13) |
| + Musculoskeletal | 25.9% | 26.4% | 1.02 (0.86–1.20) | 43.6% | 46.9% | 1.08 (0.97–1.18) |
| + Mental disorder | 21.1% | 28.1% | 1.33 (1.13–1.53) | 38.7% | 41.3% | 1.07 (0.96–1.20) |
| + Respiratory | 11.8% | 11.4% | 0.97 (0.70–1.23) | 15.4% | 20.6% | 1.34 (1.11–1.60) |
| + Cancer | 5.0% | 4.0% | 0.81 (0.47–1.23) | 6.8% | 8.5% | 1.24 (0.89–1.67) |
| + Stomach ulcer | 8.7% | 12.9% | 1.47 (1.13–1.86) | 9.5% | 15.2% | 1.60 (1.25–1.96) |
| + Dementia | 1.4% | 2.5% | 1.83 (0.84–3.25) | 1.2% | 1.7% | 1.43 (0.62–2.73) |
| Respiratory |  |  |  |  |  |  |
| + Cardiovascular | 54.8% | 56.8% | 1.04 (0.93–1.15) | 51.7% | 58.3% | 1.13 (1.04–1.22) |
| + Musculoskeletal | 34.7% | 37.0% | 1.07 (0.89–1.25) | 51.9% | 50.0% | 0.96 (0.87–1.05) |
| + Mental disorder | 30.4% | 36.6% | 1.21 (1.03–1.42) | 45.4% | 54.2% | 1.19 (1.09–1.29) |
| + Diabetes | 16.5% | 19.8% | 1.20 (0.89–1.53) | 15.0% | 20.6% | 1.37 (1.14–1.64) |
| + Cancer | 6.3% | 6.6% | 1.05 (0.61–1.60) | 7.5% | 9.4% | 1.25 (0.89–1.65) |
| + Stomach ulcer | 14.0% | 14.7% | 1.05 (0.73–1.41) | 12.1% | 19.2% | 1.59 (1.29–1.91) |
| + Dementia | 2.1% | 1.8% | 0.87 (0.17–1.92) | 1.8% | 1.7% | 0.96 (0.36–1.68) |
| Cancer |  |  |  |  |  |  |
| + Cardiovascular | 49.5% | 41.1% | 0.83 (0.64–1.02) | 39.8% | 49.4% | 1.24 (1.09–1.39) |
| + Musculoskeletal | 22.1% | 25.9% | 1.17 (0.79–1.56) | 37.9% | 39.5% | 1.04 (0.90–1.19) |
| + Mental disorder | 24.4% | 26.8% | 1.10 (0.78–1.46) | 35.8% | 39.2% | 1.10 (0.94–1.25) |
| + Diabetes | 14.5% | 17.0% | 1.17 (0.70–1.74) | 8.5% | 13.2% | 1.56 (1.12–2.08) |
| + Respiratory | 13.0% | 16.1% | 1.23 (0.72–1.82) | 9.6% | 14.7% | 1.52 (1.14–1.98) |
| + Stomach ulcer | 10.1% | 14.3% | 1.41 (0.78–2.18) | 9.9% | 15.6% | 1.58 (1.15–2.06) |
| + Dementia | 1.9% | 0.9% | 0.46 (0.00–1.63) | 1.0% | 0.9% | 0.87 (0.00–2.30) |
| Stomach ulcer |  |  |  |  |  |  |
| + Cardiovascular | 52.4% | 43.1% | 0.82 (0.72–0.93) | 49.1% | 57.6% | 1.17 (1.07–1.27) |
| + Musculoskeletal | 33.5% | 32.8% | 0.98 (0.84–1.14) | 52.5% | 55.5% | 1.06 (0.97–1.15) |
| + Mental disorder | 31.7% | 30.5% | 0.96 (0.80–1.11) | 47.3% | 56.5% | 1.20 (1.09–1.30) |
| + Diabetes | 12.9% | 17.1% | 1.32 (1.02–1.70) | 12.0% | 16.4% | 1.37 (1.09–1.72) |
| + Respiratory | 14.8% | 11.2% | 0.76 (0.54–1.00) | 15.7% | 20.8% | 1.32 (1.06–1.58) |
| + Cancer | 5.1% | 4.5% | 0.88 (0.49–1.33) | 10.0% | 10.8% | 1.08 (0.80–1.38) |
| + Dementia | 1.6% | 2.2% | 1.42 (0.53–2.78) | 1.7% | 1.7% | 0.96 (0.33–1.81) |
| Dementia |  |  |  |  |  |  |
| + Cardiovascular | 58.7% | 35.5% | 0.60 (0.31–0.92) | 60.6% | 71.4% | 1.18 (0.93–1.43) |
| + Musculoskeletal | 34.4% | 38.7% | 1.12 (0.67–1.74) | 52.1% | 52.4% | 1.01 (0.71–1.33) |
| + Mental disorder | 67.4% | 77.4% | 1.15 (0.90–1.40) | 72.1% | 71.4% | 0.99 (0.79–1.19) |
| + Diabetes | 17.4% | 38.7% | 2.23 (1.23–3.45) | 14.0% | 21.4% | 1.53 (0.61–2.71) |
| + Respiratory | 18.8% | 16.1% | 0.86 (0.29–1.78) | 21.3% | 21.4% | 1.01 (0.46–1.71) |
| + Cancer | 8.3% | 3.2% | 0.39 (0.00–1.41) | 9.5% | 7.1% | 0.75 (0.00–1.79) |
| + Stomach ulcer | 13.4% | 25.8% | 1.93 (0.72–3.37) | 15.9% | 19.0% | 1.20 (0.46–2.12) |
| **60–69** |  |  |  |  |  |  |
| Cardiovascular |  |  |  |  |  |  |
| + Musculoskeletal | 31.3% | 34.4% | 1.10 (1.05–1.16) | 51.1% | 55.8% | 1.09 (1.06–1.13) |
| + Mental disorder | 18.8% | 21.4% | 1.14 (1.06–1.22) | 33.9% | 36.5% | 1.08 (1.04–1.13) |
| + Diabetes | 23.8% | 26.0% | 1.09 (1.02–1.17) | 20.5% | 25.3% | 1.23 (1.16–1.30) |
| + Respiratory | 11.9% | 14.5% | 1.22 (1.12–1.33) | 12.2% | 16.6% | 1.36 (1.27–1.47) |
| + Cancer | 7.4% | 7.7% | 1.05 (0.91–1.18) | 9.5% | 10.1% | 1.06 (0.96–1.17) |
| + Stomach ulcer | 9.1% | 11.1% | 1.22 (1.10–1.35) | 9.5% | 13.9% | 1.47 (1.34–1.60) |
| + Dementia | 2.1% | 2.2% | 1.03 (0.76–1.29) | 2.0% | 2.1% | 1.04 (0.80–1.29) |
| Musculoskeletal |  |  |  |  |  |  |
| + Cardiovascular | 68.0% | 68.7% | 1.01 (0.98–1.04) | 61.7% | 66.1% | 1.07 (1.04–1.10) |
| + Mental disorder | 25.4% | 27.2% | 1.07 (0.98–1.18) | 39.9% | 43.0% | 1.08 (1.04–1.12) |
| + Diabetes | 21.1% | 25.2% | 1.20 (1.09–1.31) | 16.0% | 20.8% | 1.30 (1.22–1.40) |
| + Respiratory | 15.9% | 16.2% | 1.02 (0.90–1.13) | 14.3% | 18.6% | 1.30 (1.20–1.40) |
| + Cancer | 8.3% | 7.9% | 0.95 (0.78–1.13) | 10.2% | 10.6% | 1.04 (0.94–1.15) |
| + Stomach ulcer | 12.9% | 15.7% | 1.22 (1.07–1.37) | 11.6% | 17.8% | 1.53 (1.41–1.65) |
| + Dementia | 2.6% | 2.0% | 0.76 (0.52–1.06) | 1.9% | 2.3% | 1.19 (0.92–1.47) |
| Mental disorder |  |  |  |  |  |  |
| + Cardiovascular | 69.0% | 72.5% | 1.05 (1.01–1.09) | 63.3% | 68.0% | 1.07 (1.04–1.11) |
| + Musculoskeletal | 42.8% | 46.3% | 1.08 (1.00–1.16) | 61.8% | 67.6% | 1.09 (1.06–1.13) |
| + Diabetes | 22.9% | 28.8% | 1.26 (1.12–1.40) | 17.8% | 23.5% | 1.32 (1.22–1.44) |
| + Respiratory | 18.6% | 21.1% | 1.14 (0.99–1.29) | 16.1% | 20.8% | 1.30 (1.19–1.41) |
| + Cancer | 10.2% | 10.1% | 0.99 (0.80–1.20) | 11.7% | 12.2% | 1.05 (0.92–1.18) |
| + Stomach ulcer | 15.6% | 20.3% | 1.30 (1.12–1.47) | 13.8% | 19.5% | 1.41 (1.28–1.54) |
| + Dementia | 5.6% | 6.3% | 1.12 (0.84–1.42) | 3.5% | 3.9% | 1.13 (0.86–1.40) |
| Diabetes |  |  |  |  |  |  |
| + Cardiovascular | 81.4% | 79.0% | 0.97 (0.94–1.00) | 84.1% | 86.6% | 1.03 (1.00–1.06) |
| + Musculoskeletal | 33.1% | 38.3% | 1.16 (1.06–1.25) | 54.2% | 60.1% | 1.11 (1.05–1.17) |
| + Mental disorder | 21.4% | 25.8% | 1.20 (1.07–1.35) | 38.9% | 43.2% | 1.11 (1.03–1.20) |
| + Respiratory | 13.7% | 15.2% | 1.11 (0.93–1.28) | 16.1% | 18.8% | 1.17 (1.03–1.33) |
| + Cancer | 7.6% | 7.6% | 1.01 (0.79–1.24) | 11.3% | 10.0% | 0.89 (0.73–1.06) |
| + Stomach ulcer | 9.3% | 13.0% | 1.40 (1.18–1.64) | 11.0% | 17.2% | 1.56 (1.35–1.80) |
| + Dementia | 2.3% | 3.1% | 1.36 (0.90–1.95) | 3.0% | 2.2% | 0.73 (0.45–1.07) |
| Respiratory |  |  |  |  |  |  |
| + Cardiovascular | 70.2% | 73.0% | 1.04 (0.99–1.09) | 67.1% | 75.7% | 1.13 (1.08–1.17) |
| + Musculoskeletal | 43.2% | 40.8% | 0.94 (0.85–1.04) | 65.3% | 71.7% | 1.10 (1.05–1.14) |
| + Mental disorder | 30.0% | 31.3% | 1.04 (0.92–1.17) | 47.3% | 51.0% | 1.08 (1.00–1.16) |
| + Diabetes | 23.7% | 25.2% | 1.06 (0.92–1.22) | 21.7% | 25.0% | 1.16 (1.02–1.30) |
| + Cancer | 10.4% | 12.5% | 1.20 (0.93–1.50) | 12.8% | 15.6% | 1.22 (1.03–1.43) |
| + Stomach ulcer | 16.4% | 17.3% | 1.06 (0.86–1.26) | 15.7% | 21.3% | 1.36 (1.17–1.55) |
| + Dementia | 3.3% | 2.0% | 0.60 (0.29–0.95) | 3.0% | 4.1% | 1.38 (0.92–1.89) |
| Cancer |  |  |  |  |  |  |
| + Cardiovascular | 64.7% | 66.6% | 1.03 (0.95–1.11) | 59.7% | 65.7% | 1.10 (1.03–1.17) |
| + Musculoskeletal | 33.7% | 34.3% | 1.02 (0.86–1.17) | 53.2% | 58.6% | 1.10 (1.03–1.18) |
| + Mental disorder | 24.5% | 25.7% | 1.05 (0.84–1.26) | 39.2% | 42.9% | 1.09 (0.99–1.20) |
| + Diabetes | 19.4% | 21.7% | 1.12 (0.89–1.36) | 17.3% | 19.1% | 1.11 (0.92–1.30) |
| + Respiratory | 15.5% | 21.4% | 1.39 (1.09–1.71) | 14.6% | 22.4% | 1.53 (1.29–1.80) |
| + Stomach ulcer | 12.1% | 16.0% | 1.33 (0.99–1.68) | 11.2% | 16.4% | 1.46 (1.21–1.74) |
| + Dementia | 2.3% | 2.3% | 0.99 (0.37–1.82) | 1.8% | 2.8% | 1.52 (0.85–2.37) |
| Stomach ulcer |  |  |  |  |  |  |
| + Cardiovascular | 67.4% | 66.6% | 0.99 (0.92–1.05) | 66.7% | 72.3% | 1.08 (1.03–1.14) |
| + Musculoskeletal | 44.0% | 47.3% | 1.07 (0.97–1.18) | 67.9% | 78.5% | 1.16 (1.11–1.21) |
| + Mental disorder | 31.7% | 36.0% | 1.14 (1.00–1.28) | 52.1% | 54.7% | 1.05 (0.97–1.13) |
| + Diabetes | 20.1% | 25.6% | 1.28 (1.08–1.48) | 18.9% | 26.2% | 1.39 (1.22–1.58) |
| + Respiratory | 20.6% | 20.7% | 1.01 (0.83–1.19) | 20.0% | 24.4% | 1.22 (1.05–1.39) |
| + Cancer | 10.2% | 11.1% | 1.09 (0.82–1.38) | 12.6% | 13.1% | 1.04 (0.85–1.27) |
| + Dementia | 3.8% | 4.0% | 1.06 (0.62–1.57) | 2.8% | 2.2% | 0.80 (0.41–1.22) |
| Dementia |  |  |  |  |  |  |
| + Cardiovascular | 74.0% | 73.0% | 0.99 (0.86–1.11) | 73.6% | 70.9% | 0.96 (0.85–1.08) |
| + Musculoskeletal | 42.2% | 33.7% | 0.80 (0.58–1.04) | 60.1% | 67.3% | 1.12 (0.97–1.27) |
| + Mental disorder | 54.0% | 62.9% | 1.16 (0.99–1.34) | 69.3% | 72.7% | 1.05 (0.92–1.18) |
| + Diabetes | 23.7% | 34.8% | 1.47 (1.03–1.96) | 27.0% | 21.8% | 0.81 (0.55–1.11) |
| + Respiratory | 19.8% | 13.5% | 0.68 (0.36–1.07) | 20.0% | 30.9% | 1.54 (1.12–2.06) |
| + Cancer | 9.3% | 9.0% | 0.96 (0.39–1.70) | 10.7% | 14.5% | 1.36 (0.73–2.06) |
| + Stomach ulcer | 17.9% | 22.5% | 1.26 (0.79–1.80) | 14.6% | 14.5% | 1.00 (0.57–1.52) |
| **70–79** |  |  |  |  |  |  |
| Cardiovascular |  |  |  |  |  |  |
| + Musculoskeletal | 38.9% | 39.1% | 1.01 (0.96–1.05) | 62.7% | 65.1% | 1.04 (1.02–1.06) |
| + Mental disorder | 20.7% | 19.5% | 0.94 (0.87–1.01) | 37.3% | 38.8% | 1.04 (1.00–1.08) |
| + Diabetes | 27.2% | 27.8% | 1.02 (0.96–1.08) | 24.8% | 28.4% | 1.14 (1.08–1.21) |
| + Respiratory | 15.1% | 16.3% | 1.08 (0.98–1.17) | 14.9% | 15.9% | 1.07 (0.99–1.14) |
| + Cancer | 12.4% | 13.2% | 1.06 (0.96–1.17) | 11.6% | 11.9% | 1.03 (0.94–1.12) |
| + Stomach ulcer | 10.3% | 13.4% | 1.30 (1.18–1.44) | 10.7% | 16.2% | 1.52 (1.40–1.63) |
| + Dementia | 5.0% | 5.6% | 1.12 (0.96–1.28) | 4.8% | 4.3% | 0.90 (0.76–1.05) |
| Musculoskeletal |  |  |  |  |  |  |
| + Cardiovascular | 80.1% | 79.9% | 1.00 (0.97–1.02) | 77.7% | 81.7% | 1.05 (1.03–1.07) |
| + Mental disorder | 26.3% | 25.6% | 0.97 (0.89–1.06) | 42.4% | 42.9% | 1.01 (0.97–1.06) |
| + Diabetes | 26.1% | 28.4% | 1.09 (1.00–1.18) | 21.8% | 26.3% | 1.21 (1.14–1.28) |
| + Respiratory | 19.2% | 19.3% | 1.01 (0.91–1.12) | 16.4% | 18.8% | 1.15 (1.06–1.23) |
| + Cancer | 13.9% | 13.0% | 0.93 (0.81–1.06) | 12.4% | 13.0% | 1.05 (0.95–1.15) |
| + Stomach ulcer | 13.0% | 16.9% | 1.30 (1.14–1.44) | 12.5% | 18.2% | 1.45 (1.35–1.57) |
| + Dementia | 5.9% | 5.3% | 0.90 (0.71–1.11) | 4.8% | 4.5% | 0.95 (0.80–1.12) |
| Mental disorder |  |  |  |  |  |  |
| + Cardiovascular | 83.0% | 81.8% | 0.99 (0.95–1.02) | 80.2% | 84.2% | 1.05 (1.03–1.07) |
| + Musculoskeletal | 51.1% | 52.4% | 1.02 (0.95–1.09) | 73.5% | 74.2% | 1.01 (0.98–1.04) |
| + Diabetes | 29.6% | 32.7% | 1.11 (0.99–1.23) | 24.7% | 30.9% | 1.25 (1.16–1.33) |
| + Respiratory | 22.5% | 22.3% | 0.99 (0.85–1.14) | 18.8% | 20.3% | 1.08 (0.98–1.19) |
| + Cancer | 16.3% | 16.2% | 0.99 (0.83–1.16) | 13.9% | 14.6% | 1.05 (0.93–1.17) |
| + Stomach ulcer | 16.9% | 22.3% | 1.32 (1.13–1.51) | 15.7% | 20.9% | 1.33 (1.20–1.46) |
| + Dementia | 12.0% | 11.4% | 0.95 (0.75–1.18) | 8.0% | 7.5% | 0.95 (0.79–1.10) |
| Diabetes |  |  |  |  |  |  |
| + Cardiovascular | 87.9% | 86.8% | 0.99 (0.96–1.01) | 90.5% | 92.1% | 1.02 (1.00–1.04) |
| + Musculoskeletal | 41.0% | 43.4% | 1.06 (0.98–1.14) | 64.1% | 68.0% | 1.06 (1.02–1.10) |
| + Mental disorder | 23.9% | 24.4% | 1.02 (0.91–1.15) | 41.9% | 46.2% | 1.10 (1.03–1.17) |
| + Respiratory | 16.7% | 17.5% | 1.05 (0.90–1.22) | 16.9% | 18.0% | 1.06 (0.93–1.19) |
| + Cancer | 12.6% | 14.2% | 1.13 (0.96–1.32) | 12.4% | 13.7% | 1.11 (0.96–1.26) |
| + Stomach ulcer | 10.3% | 12.8% | 1.24 (1.03–1.46) | 12.2% | 18.5% | 1.52 (1.35–1.73) |
| + Dementia | 5.9% | 7.0% | 1.18 (0.89–1.50) | 6.2% | 6.5% | 1.04 (0.82–1.27) |
| Respiratory |  |  |  |  |  |  |
| + Cardiovascular | 81.4% | 81.1% | 1.00 (0.95–1.04) | 83.7% | 82.7% | 0.99 (0.95–1.02) |
| + Musculoskeletal | 50.2% | 47.1% | 0.94 (0.85–1.02) | 74.1% | 77.9% | 1.05 (1.01–1.09) |
| + Mental disorder | 30.3% | 26.5% | 0.87 (0.76–1.00) | 49.0% | 48.7% | 0.99 (0.92–1.07) |
| + Diabetes | 27.9% | 28.0% | 1.00 (0.87–1.13) | 26.0% | 28.9% | 1.11 (0.97–1.24) |
| + Cancer | 15.1% | 20.4% | 1.35 (1.14–1.59) | 14.5% | 17.7% | 1.23 (1.04–1.43) |
| + Stomach ulcer | 16.3% | 19.6% | 1.20 (1.00–1.40) | 15.9% | 25.5% | 1.61 (1.40–1.83) |
| + Dementia | 7.3% | 6.0% | 0.82 (0.58–1.11) | 6.3% | 5.8% | 0.91 (0.65–1.18) |
| Cancer |  |  |  |  |  |  |
| + Cardiovascular | 77.4% | 81.5% | 1.05 (1.00–1.10) | 75.7% | 81.1% | 1.07 (1.03–1.12) |
| + Musculoskeletal | 42.1% | 39.2% | 0.93 (0.83–1.04) | 65.5% | 70.7% | 1.08 (1.02–1.14) |
| + Mental disorder | 25.3% | 23.8% | 0.94 (0.80–1.10) | 42.3% | 46.0% | 1.09 (0.99–1.19) |
| + Diabetes | 24.3% | 28.1% | 1.16 (0.98–1.33) | 22.1% | 28.8% | 1.30 (1.14–1.49) |
| + Respiratory | 17.5% | 25.3% | 1.45 (1.24–1.66) | 16.8% | 23.2% | 1.38 (1.19–1.60) |
| + Stomach ulcer | 13.4% | 19.9% | 1.49 (1.23–1.77) | 12.3% | 18.9% | 1.53 (1.27–1.81) |
| + Dementia | 5.3% | 6.6% | 1.24 (0.85–1.66) | 4.8% | 4.9% | 1.02 (0.64–1.41) |
| Stomach ulcer |  |  |  |  |  |  |
| + Cardiovascular | 80.4% | 81.5% | 1.01 (0.97–1.06) | 82.5% | 87.8% | 1.06 (1.03–1.10) |
| + Musculoskeletal | 49.2% | 50.0% | 1.02 (0.92–1.11) | 77.9% | 78.5% | 1.01 (0.96–1.05) |
| + Mental disorder | 32.9% | 32.3% | 0.98 (0.85–1.11) | 56.6% | 52.3% | 0.92 (0.85–0.99) |
| + Diabetes | 24.8% | 24.8% | 1.00 (0.85–1.17) | 25.8% | 30.9% | 1.20 (1.06–1.33) |
| + Respiratory | 23.6% | 23.8% | 1.01 (0.84–1.19) | 21.9% | 26.6% | 1.22 (1.07–1.38) |
| + Cancer | 16.7% | 19.6% | 1.17 (0.94–1.39) | 14.6% | 15.0% | 1.03 (0.84–1.22) |
| + Dementia | 6.9% | 6.5% | 0.94 (0.64–1.30) | 6.3% | 6.1% | 0.98 (0.72–1.28) |
| Dementia |  |  |  |  |  |  |
| + Cardiovascular | 83.5% | 87.1% | 1.04 (0.98–1.10) | 82.9% | 80.8% | 0.98 (0.91–1.04) |
| + Musculoskeletal | 47.4% | 40.2% | 0.85 (0.71–1.00) | 67.2% | 68.7% | 1.02 (0.93–1.12) |
| + Mental disorder | 50.0% | 42.3% | 0.85 (0.70–1.00) | 64.8% | 65.9% | 1.02 (0.92–1.12) |
| + Diabetes | 30.4% | 34.5% | 1.14 (0.91–1.38) | 29.8% | 37.9% | 1.27 (1.03–1.51) |
| + Respiratory | 22.4% | 18.6% | 0.83 (0.59–1.10) | 19.7% | 21.0% | 1.07 (0.80–1.38) |
| + Cancer | 14.2% | 16.5% | 1.16 (0.80–1.58) | 12.8% | 13.6% | 1.06 (0.70–1.45) |
| + Stomach ulcer | 14.7% | 16.5% | 1.12 (0.77–1.52) | 14.2% | 21.5% | 1.51 (1.12–1.93) |

## **Table S6.** Prevalence and relative risk of chronic disease combinations for immigrants versus native-born individuals by gender and origin country group

|  | **Men** | | | **Women** | | |
| --- | --- | --- | --- | --- | --- | --- |
|  | **Native-born** | **Immigrant** | **RR (95% CI)** | **Native-born** | **Immigrant** | **RR (95% CI)** |
| **Africa** |  |  |  |  |  |  |
| Cardiovascular |  |  |  |  |  |  |
| + Musculoskeletal | 29.9% | 33.1% | 1.11 (0.97–1.24) | 48.5% | 59.8% | 1.23 (1.15–1.32) |
| + Mental disorder | 19.0% | 23.8% | 1.25 (1.09–1.43) | 33.8% | 43.0% | 1.27 (1.15–1.39) |
| + Diabetes | 21.9% | 31.7% | 1.45 (1.28–1.62) | 19.0% | 29.7% | 1.56 (1.37–1.76) |
| + Respiratory | 11.7% | 16.7% | 1.43 (1.18–1.70) | 12.3% | 13.9% | 1.14 (0.92–1.37) |
| + Cancer | 7.4% | 7.4% | 1.01 (0.74–1.29) | 8.8% | 9.7% | 1.10 (0.82–1.37) |
| + Stomach ulcer | 9.1% | 7.5% | 0.83 (0.60–1.06) | 9.1% | 12.2% | 1.34 (1.05–1.65) |
| + Dementia | 2.5% | 1.7% | 0.70 (0.32–1.14) | 2.3% | 2.2% | 0.93 (0.44–1.43) |
| Musculoskeletal |  |  |  |  |  |  |
| + Cardiovascular | 64.6% | 60.8% | 0.94 (0.86–1.01) | 59.2% | 57.0% | 0.96 (0.89–1.03) |
| + Mental disorder | 25.5% | 23.9% | 0.94 (0.75–1.13) | 39.8% | 48.0% | 1.21 (1.11–1.30) |
| + Diabetes | 19.5% | 28.9% | 1.48 (1.24–1.73) | 15.4% | 23.4% | 1.52 (1.29–1.75) |
| + Respiratory | 15.7% | 16.2% | 1.03 (0.79–1.29) | 14.3% | 14.9% | 1.04 (0.85–1.24) |
| + Cancer | 8.1% | 8.2% | 1.01 (0.65–1.40) | 9.7% | 8.7% | 0.90 (0.64–1.13) |
| + Stomach ulcer | 12.6% | 15.5% | 1.23 (0.94–1.56) | 11.2% | 15.6% | 1.38 (1.13–1.64) |
| + Dementia | 3.0% | 1.0% | 0.33 (0.00–0.71) | 2.4% | 1.4% | 0.61 (0.27–1.01) |
| Mental disorder |  |  |  |  |  |  |
| + Cardiovascular | 65.9% | 70.0% | 1.06 (0.97–1.16) | 60.7% | 54.9% | 0.90 (0.83–0.97) |
| + Musculoskeletal | 40.7% | 38.5% | 0.95 (0.79–1.12) | 58.3% | 63.5% | 1.09 (1.02–1.16) |
| + Diabetes | 21.3% | 29.6% | 1.39 (1.11–1.69) | 17.0% | 22.0% | 1.29 (1.08–1.53) |
| + Respiratory | 18.1% | 21.7% | 1.20 (0.89–1.51) | 15.7% | 16.9% | 1.08 (0.87–1.30) |
| + Cancer | 10.0% | 15.8% | 1.58 (1.12–2.16) | 10.9% | 13.8% | 1.26 (0.99–1.56) |
| + Stomach ulcer | 15.6% | 14.3% | 0.92 (0.63–1.23) | 13.1% | 13.4% | 1.02 (0.78–1.25) |
| + Dementia | 6.5% | 3.0% | 0.45 (0.15–0.83) | 4.0% | 3.3% | 0.82 (0.43–1.24) |
| Diabetes |  |  |  |  |  |  |
| + Cardiovascular | 79.1% | 70.2% | 0.89 (0.82–0.96) | 80.1% | 73.4% | 0.92 (0.85–0.99) |
| + Musculoskeletal | 32.3% | 34.6% | 1.07 (0.89–1.25) | 52.6% | 60.8% | 1.16 (1.03–1.27) |
| + Mental disorder | 21.9% | 22.1% | 1.01 (0.79–1.24) | 39.6% | 43.2% | 1.09 (0.93–1.26) |
| + Respiratory | 13.7% | 15.3% | 1.11 (0.82–1.46) | 16.0% | 8.2% | 0.51 (0.31–0.74) |
| + Cancer | 7.9% | 6.4% | 0.81 (0.45–1.18) | 9.8% | 13.4% | 1.37 (0.95–1.81) |
| + Stomach ulcer | 9.3% | 6.3% | 0.68 (0.39–1.00) | 10.7% | 16.7% | 1.56 (1.12–1.98) |
| + Dementia | 2.9% | 1.7% | 0.58 (0.13–1.22) | 3.1% | 0.0% | 0.00 (0.00–0.00) |
| Respiratory |  |  |  |  |  |  |
| + Cardiovascular | 67.1% | 66.7% | 0.99 (0.89–1.10) | 65.4% | 66.1% | 1.01 (0.88–1.14) |
| + Musculoskeletal | 41.7% | 34.7% | 0.83 (0.64–1.02) | 62.3% | 72.8% | 1.17 (1.03–1.29) |
| + Mental disorder | 30.2% | 28.5% | 0.94 (0.70–1.20) | 47.0% | 63.6% | 1.35 (1.17–1.53) |
| + Diabetes | 21.9% | 27.5% | 1.26 (0.96–1.59) | 20.2% | 16.9% | 0.84 (0.53–1.17) |
| + Cancer | 10.0% | 13.0% | 1.30 (0.78–1.85) | 11.2% | 16.1% | 1.44 (0.88–2.07) |
| + Stomach ulcer | 15.4% | 17.2% | 1.11 (0.73–1.51) | 14.3% | 12.3% | 0.86 (0.50–1.30) |
| + Dementia | 3.9% | 3.1% | 0.80 (0.17–1.67) | 3.4% | 0.8% | 0.23 (0.00–0.73) |
| Cancer |  |  |  |  |  |  |
| + Cardiovascular | 62.0% | 60.0% | 0.97 (0.79–1.16) | 56.0% | 57.0% | 1.02 (0.86–1.17) |
| + Musculoskeletal | 31.3% | 39.0% | 1.24 (0.86–1.60) | 50.4% | 53.9% | 1.07 (0.87–1.26) |
| + Mental disorder | 24.7% | 45.4% | 1.84 (1.36–2.36) | 38.7% | 63.5% | 1.64 (1.40–1.88) |
| + Diabetes | 18.7% | 23.5% | 1.26 (0.71–1.83) | 15.1% | 30.7% | 2.04 (1.44–2.66) |
| + Respiratory | 15.0% | 29.4% | 1.96 (1.25–2.69) | 13.2% | 20.2% | 1.53 (0.93–2.12) |
| + Stomach ulcer | 11.6% | 13.8% | 1.19 (0.52–1.97) | 11.0% | 20.4% | 1.86 (1.16–2.61) |
| + Dementia | 3.0% | 2.7% | 0.92 (0.00–2.44) | 2.3% | 0.0% | 0.00 (0.00–0.00) |
| Stomach ulcer |  |  |  |  |  |  |
| + Cardiovascular | 64.9% | 52.2% | 0.80 (0.64–0.97) | 63.9% | 60.5% | 0.95 (0.82–1.08) |
| + Musculoskeletal | 41.2% | 52.4% | 1.27 (1.02–1.53) | 64.5% | 78.4% | 1.22 (1.10–1.33) |
| + Mental disorder | 32.0% | 31.7% | 0.99 (0.68–1.28) | 51.4% | 50.9% | 0.99 (0.83–1.18) |
| + Diabetes | 18.5% | 21.7% | 1.17 (0.73–1.65) | 18.0% | 31.6% | 1.76 (1.28–2.25) |
| + Respiratory | 19.1% | 28.8% | 1.51 (1.03–2.00) | 18.8% | 12.7% | 0.67 (0.37–1.01) |
| + Cancer | 9.9% | 11.0% | 1.11 (0.54–1.73) | 12.1% | 16.5% | 1.37 (0.82–1.95) |
| + Dementia | 3.7% | 4.2% | 1.14 (0.27–2.39) | 3.3% | 0.0% | 0.00 (0.00–0.00) |
| Dementia |  |  |  |  |  |  |
| + Cardiovascular | 70.4% | 100.0% | 1.42 (1.39–1.45) | 70.9% | 60.6% | 0.86 (0.54–1.14) |
| + Musculoskeletal | 40.5% | 22.6% | 0.56 (0.00–1.15) | 58.8% | 33.0% | 0.56 (0.24–0.90) |
| + Mental disorder | 58.3% | 81.1% | 1.39 (0.93–1.73) | 69.2% | 72.5% | 1.05 (0.76–1.32) |
| + Diabetes | 23.0% | 77.3% | 3.37 (2.25–4.41) | 22.6% | 0.0% | 0.00 (0.00–0.00) |
| + Respiratory | 20.1% | 33.9% | 1.69 (0.45–3.27) | 20.4% | 4.8% | 0.24 (0.00–0.71) |
| + Cancer | 10.2% | 11.3% | 1.11 (0.00–3.44) | 10.8% | 0.0% | 0.00 (0.00–0.00) |
| + Stomach ulcer | 15.3% | 15.1% | 0.99 (0.00–2.45) | 15.0% | 0.0% | 0.00 (0.00–0.00) |
| **Asia & Oceania** |  |  |  |  |  |  |
| Cardiovascular |  |  |  |  |  |  |
| + Musculoskeletal | 29.9% | 29.6% | 0.99 (0.85–1.14) | 48.5% | 51.4% | 1.06 (0.96–1.15) |
| + Mental disorder | 19.0% | 25.7% | 1.35 (1.13–1.59) | 33.8% | 32.8% | 0.97 (0.86–1.10) |
| + Diabetes | 21.9% | 28.9% | 1.32 (1.12–1.54) | 19.0% | 33.0% | 1.74 (1.53–1.98) |
| + Respiratory | 11.7% | 18.6% | 1.59 (1.27–1.94) | 12.3% | 13.8% | 1.13 (0.87–1.38) |
| + Cancer | 7.4% | 4.3% | 0.59 (0.31–0.88) | 8.8% | 9.6% | 1.10 (0.80–1.40) |
| + Stomach ulcer | 9.1% | 13.6% | 1.50 (1.11–1.89) | 9.1% | 17.4% | 1.91 (1.56–2.27) |
| + Dementia | 2.5% | 1.2% | 0.48 (0.10–0.92) | 2.3% | 3.0% | 1.30 (0.69–2.00) |
| Musculoskeletal |  |  |  |  |  |  |
| + Cardiovascular | 64.6% | 56.0% | 0.87 (0.76–0.98) | 59.2% | 64.7% | 1.09 (1.01–1.17) |
| + Mental disorder | 25.5% | 23.3% | 0.91 (0.71–1.13) | 39.8% | 34.5% | 0.87 (0.75–0.98) |
| + Diabetes | 19.5% | 22.0% | 1.13 (0.86–1.41) | 15.4% | 27.9% | 1.82 (1.51–2.11) |
| + Respiratory | 15.7% | 13.8% | 0.88 (0.57–1.19) | 14.3% | 14.3% | 1.00 (0.77–1.25) |
| + Cancer | 8.1% | 4.9% | 0.60 (0.29–0.96) | 9.7% | 7.1% | 0.73 (0.46–1.02) |
| + Stomach ulcer | 12.6% | 17.8% | 1.41 (1.01–1.82) | 11.2% | 22.1% | 1.96 (1.60–2.33) |
| + Dementia | 3.0% | 4.4% | 1.45 (0.63–2.41) | 2.4% | 2.7% | 1.16 (0.53–1.86) |
| Mental disorder |  |  |  |  |  |  |
| + Cardiovascular | 65.9% | 70.5% | 1.07 (0.95–1.18) | 60.7% | 65.6% | 1.08 (0.97–1.18) |
| + Musculoskeletal | 40.7% | 39.1% | 0.96 (0.76–1.15) | 58.3% | 55.9% | 0.96 (0.86–1.06) |
| + Diabetes | 21.3% | 37.8% | 1.77 (1.38–2.17) | 17.0% | 27.9% | 1.65 (1.33–1.98) |
| + Respiratory | 18.1% | 29.7% | 1.64 (1.21–2.10) | 15.7% | 19.5% | 1.24 (0.92–1.57) |
| + Cancer | 10.0% | 5.5% | 0.55 (0.21–0.99) | 10.9% | 9.8% | 0.90 (0.57–1.25) |
| + Stomach ulcer | 15.6% | 23.4% | 1.50 (1.08–1.99) | 13.1% | 27.2% | 2.07 (1.63–2.51) |
| + Dementia | 6.5% | 5.7% | 0.88 (0.33–1.48) | 4.0% | 5.6% | 1.41 (0.71–2.20) |
| Diabetes |  |  |  |  |  |  |
| + Cardiovascular | 79.1% | 71.7% | 0.91 (0.82–0.99) | 80.1% | 87.0% | 1.09 (1.03–1.14) |
| + Musculoskeletal | 32.3% | 29.5% | 0.91 (0.71–1.14) | 52.6% | 58.1% | 1.10 (0.98–1.24) |
| + Mental disorder | 21.9% | 33.0% | 1.50 (1.15–1.84) | 39.6% | 36.1% | 0.91 (0.73–1.08) |
| + Respiratory | 13.7% | 14.2% | 1.04 (0.65–1.47) | 16.0% | 14.4% | 0.90 (0.59–1.23) |
| + Cancer | 7.9% | 2.2% | 0.28 (0.00–0.64) | 9.8% | 9.7% | 0.99 (0.60–1.48) |
| + Stomach ulcer | 9.3% | 14.7% | 1.57 (1.00–2.21) | 10.7% | 20.3% | 1.89 (1.41–2.46) |
| + Dementia | 2.9% | 3.2% | 1.10 (0.23–2.15) | 3.1% | 6.8% | 2.18 (1.13–3.44) |
| Respiratory |  |  |  |  |  |  |
| + Cardiovascular | 67.1% | 76.4% | 1.14 (1.01–1.26) | 65.4% | 67.0% | 1.02 (0.89–1.16) |
| + Musculoskeletal | 41.7% | 30.5% | 0.73 (0.51–0.95) | 62.3% | 55.7% | 0.89 (0.75–1.05) |
| + Mental disorder | 30.2% | 43.0% | 1.42 (1.08–1.74) | 47.0% | 46.7% | 0.99 (0.78–1.21) |
| + Diabetes | 21.9% | 23.2% | 1.06 (0.67–1.49) | 20.2% | 27.9% | 1.39 (0.96–1.81) |
| + Cancer | 10.0% | 4.4% | 0.44 (0.10–0.84) | 11.2% | 7.9% | 0.71 (0.33–1.21) |
| + Stomach ulcer | 15.4% | 19.1% | 1.24 (0.79–1.77) | 14.3% | 25.6% | 1.79 (1.16–2.39) |
| + Dementia | 3.9% | 0.0% | 0.00 (0.00–0.00) | 3.4% | 5.4% | 1.58 (0.30–3.11) |
| Cancer |  |  |  |  |  |  |
| + Cardiovascular | 62.0% | 72.8% | 1.17 (0.87–1.47) | 56.0% | 74.5% | 1.33 (1.15–1.51) |
| + Musculoskeletal | 31.3% | 41.5% | 1.33 (0.79–1.92) | 50.4% | 44.3% | 0.88 (0.64–1.12) |
| + Mental disorder | 24.7% | 45.7% | 1.85 (1.02–2.67) | 38.7% | 35.9% | 0.93 (0.64–1.21) |
| + Diabetes | 18.7% | 7.9% | 0.42 (0.00–1.11) | 15.1% | 31.7% | 2.11 (1.34–2.90) |
| + Respiratory | 15.0% | 31.8% | 2.11 (1.06–3.43) | 13.2% | 13.7% | 1.04 (0.46–1.70) |
| + Stomach ulcer | 11.6% | 18.1% | 1.56 (0.36–2.92) | 11.0% | 1.5% | 0.13 (0.00–0.43) |
| + Dementia | 3.0% | 0.0% | 0.00 (0.00–0.00) | 2.3% | 0.0% | 0.00 (0.00–0.00) |
| Stomach ulcer |  |  |  |  |  |  |
| + Cardiovascular | 64.9% | 64.9% | 1.00 (0.85–1.15) | 63.9% | 69.5% | 1.09 (0.96–1.22) |
| + Musculoskeletal | 41.2% | 46.3% | 1.12 (0.87–1.39) | 64.5% | 70.9% | 1.10 (0.98–1.22) |
| + Mental disorder | 32.0% | 38.8% | 1.21 (0.88–1.53) | 51.4% | 53.1% | 1.03 (0.85–1.20) |
| + Diabetes | 18.5% | 27.2% | 1.47 (0.94–1.97) | 18.0% | 32.7% | 1.82 (1.39–2.27) |
| + Respiratory | 19.1% | 23.9% | 1.25 (0.79–1.75) | 18.8% | 20.8% | 1.11 (0.71–1.53) |
| + Cancer | 9.9% | 5.2% | 0.52 (0.12–1.07) | 12.1% | 0.8% | 0.06 (0.00–0.20) |
| + Dementia | 3.7% | 7.1% | 1.90 (0.62–3.56) | 3.3% | 8.9% | 2.69 (1.26–4.34) |
| Dementia |  |  |  |  |  |  |
| + Cardiovascular | 70.4% | 23.7% | 0.34 (0.10–0.70) | 70.9% | 88.4% | 1.25 (1.03–1.42) |
| + Musculoskeletal | 40.5% | 76.7% | 1.89 (1.36–2.37) | 58.8% | 67.7% | 1.15 (0.80–1.49) |
| + Mental disorder | 58.3% | 67.9% | 1.16 (0.74–1.58) | 69.2% | 74.9% | 1.08 (0.76–1.35) |
| + Diabetes | 23.0% | 23.3% | 1.02 (0.30–1.98) | 22.6% | 72.5% | 3.21 (2.34–4.12) |
| + Respiratory | 20.1% | 0.0% | 0.00 (0.00–0.00) | 20.4% | 16.4% | 0.80 (0.00–1.77) |
| + Cancer | 10.2% | 0.0% | 0.00 (0.00–0.00) | 10.8% | 0.0% | 0.00 (0.00–0.00) |
| + Stomach ulcer | 15.3% | 58.2% | 3.81 (1.96–5.63) | 15.0% | 67.7% | 4.51 (3.07–5.82) |
| **Latin America and the Caribbean** |  |  |  |  |  |  |
| Cardiovascular |  |  |  |  |  |  |
| + Musculoskeletal | 29.9% | 30.2% | 1.01 (0.77–1.26) | 48.5% | 50.8% | 1.05 (0.92–1.19) |
| + Mental disorder | 19.0% | 18.9% | 0.99 (0.69–1.31) | 33.8% | 42.0% | 1.24 (1.06–1.44) |
| + Diabetes | 21.9% | 28.8% | 1.31 (1.01–1.66) | 19.0% | 20.7% | 1.09 (0.81–1.35) |
| + Respiratory | 11.7% | 5.5% | 0.47 (0.21–0.81) | 12.3% | 11.6% | 0.94 (0.61–1.29) |
| + Cancer | 7.4% | 5.0% | 0.68 (0.25–1.17) | 8.8% | 11.6% | 1.32 (0.85–1.89) |
| + Stomach ulcer | 9.1% | 4.4% | 0.49 (0.20–0.88) | 9.1% | 12.5% | 1.37 (0.93–1.87) |
| + Dementia | 2.5% | 1.4% | 0.58 (0.00–1.30) | 2.3% | 3.2% | 1.36 (0.40–2.51) |
| Musculoskeletal |  |  |  |  |  |  |
| + Cardiovascular | 64.6% | 72.9% | 1.13 (0.96–1.28) | 59.2% | 56.4% | 0.95 (0.84–1.07) |
| + Mental disorder | 25.5% | 21.8% | 0.86 (0.47–1.27) | 39.8% | 45.1% | 1.13 (0.96–1.30) |
| + Diabetes | 19.5% | 26.4% | 1.35 (0.82–1.93) | 15.4% | 20.5% | 1.34 (1.00–1.72) |
| + Respiratory | 15.7% | 10.7% | 0.68 (0.28–1.19) | 14.3% | 11.8% | 0.83 (0.49–1.15) |
| + Cancer | 8.1% | 9.6% | 1.18 (0.36–2.03) | 9.7% | 11.2% | 1.16 (0.71–1.64) |
| + Stomach ulcer | 12.6% | 10.3% | 0.81 (0.35–1.42) | 11.2% | 13.5% | 1.20 (0.80–1.66) |
| + Dementia | 3.0% | 4.8% | 1.60 (0.00–3.40) | 2.4% | 2.1% | 0.90 (0.21–1.85) |
| Mental disorder |  |  |  |  |  |  |
| + Cardiovascular | 65.9% | 53.6% | 0.81 (0.64–0.98) | 60.7% | 67.9% | 1.12 (0.99–1.24) |
| + Musculoskeletal | 40.7% | 27.0% | 0.66 (0.40–0.93) | 58.3% | 65.8% | 1.13 (1.01–1.25) |
| + Diabetes | 21.3% | 29.8% | 1.40 (0.91–1.91) | 17.0% | 26.2% | 1.54 (1.11–1.99) |
| + Respiratory | 18.1% | 0.0% | 0.00 (0.00–0.00) | 15.7% | 14.0% | 0.89 (0.56–1.28) |
| + Cancer | 10.0% | 1.3% | 0.13 (0.00–0.45) | 10.9% | 15.4% | 1.41 (0.88–1.94) |
| + Stomach ulcer | 15.6% | 2.5% | 0.16 (0.00–0.39) | 13.1% | 16.3% | 1.24 (0.82–1.71) |
| + Dementia | 6.5% | 9.6% | 1.47 (0.46–2.74) | 4.0% | 4.4% | 1.10 (0.34–2.01) |
| Diabetes |  |  |  |  |  |  |
| + Cardiovascular | 79.1% | 73.8% | 0.93 (0.80–1.06) | 80.1% | 75.8% | 0.95 (0.80–1.08) |
| + Musculoskeletal | 32.3% | 23.0% | 0.71 (0.40–1.04) | 52.6% | 67.5% | 1.28 (1.05–1.52) |
| + Mental disorder | 21.9% | 30.8% | 1.40 (0.87–1.94) | 39.6% | 51.9% | 1.31 (0.99–1.63) |
| + Respiratory | 13.7% | 16.1% | 1.17 (0.57–1.84) | 16.0% | 5.6% | 0.35 (0.00–0.77) |
| + Cancer | 7.9% | 6.7% | 0.85 (0.20–1.75) | 9.8% | 15.1% | 1.54 (0.71–2.56) |
| + Stomach ulcer | 9.3% | 3.9% | 0.41 (0.00–1.00) | 10.7% | 5.8% | 0.54 (0.00–1.19) |
| + Dementia | 2.9% | 1.7% | 0.57 (0.00–1.70) | 3.1% | 2.2% | 0.70 (0.00–2.29) |
| Respiratory |  |  |  |  |  |  |
| + Cardiovascular | 67.1% | 37.9% | 0.56 (0.27–0.88) | 65.4% | 71.2% | 1.09 (0.88–1.30) |
| + Musculoskeletal | 41.7% | 15.4% | 0.37 (0.00–0.77) | 62.3% | 62.7% | 1.01 (0.76–1.25) |
| + Mental disorder | 30.2% | 0.0% | 0.00 (0.00–0.00) | 47.0% | 53.8% | 1.14 (0.80–1.44) |
| + Diabetes | 21.9% | 36.1% | 1.65 (0.80–2.65) | 20.2% | 12.9% | 0.64 (0.13–1.15) |
| + Cancer | 10.0% | 53.3% | 5.32 (3.20–7.29) | 11.2% | 9.7% | 0.87 (0.22–1.79) |
| + Stomach ulcer | 15.4% | 0.0% | 0.00 (0.00–0.00) | 14.3% | 25.6% | 1.79 (0.87–2.79) |
| + Dementia | 3.9% | 0.0% | 0.00 (0.00–0.00) | 3.4% | 6.4% | 1.87 (0.00–4.46) |
| Cancer |  |  |  |  |  |  |
| + Cardiovascular | 62.0% | 25.1% | 0.40 (0.20–0.66) | 56.0% | 65.8% | 1.17 (0.90–1.44) |
| + Musculoskeletal | 31.3% | 17.8% | 0.57 (0.20–1.00) | 50.4% | 56.6% | 1.12 (0.77–1.42) |
| + Mental disorder | 24.7% | 4.0% | 0.16 (0.00–0.51) | 38.7% | 55.2% | 1.43 (1.01–1.85) |
| + Diabetes | 18.7% | 13.0% | 0.69 (0.17–1.35) | 15.1% | 21.2% | 1.41 (0.55–2.36) |
| + Respiratory | 15.0% | 30.6% | 2.04 (1.03–3.16) | 13.2% | 13.1% | 0.99 (0.21–1.90) |
| + Stomach ulcer | 11.6% | 4.0% | 0.34 (0.00–1.04) | 11.0% | 22.4% | 2.04 (0.99–3.35) |
| + Dementia | 3.0% | 0.0% | 0.00 (0.00–0.00) | 2.3% | 0.0% | 0.00 (0.00–0.00) |
| Stomach ulcer |  |  |  |  |  |  |
| + Cardiovascular | 64.9% | 66.6% | 1.03 (0.64–1.42) | 63.9% | 70.0% | 1.10 (0.86–1.30) |
| + Musculoskeletal | 41.2% | 66.6% | 1.62 (1.00–2.24) | 64.5% | 69.5% | 1.08 (0.84–1.30) |
| + Mental disorder | 32.0% | 13.4% | 0.42 (0.00–1.04) | 51.4% | 55.8% | 1.09 (0.78–1.38) |
| + Diabetes | 18.5% | 33.1% | 1.79 (0.45–3.25) | 18.0% | 9.4% | 0.52 (0.00–1.08) |
| + Respiratory | 19.1% | 0.0% | 0.00 (0.00–0.00) | 18.8% | 23.4% | 1.25 (0.57–2.02) |
| + Cancer | 9.9% | 6.6% | 0.67 (0.00–2.49) | 12.1% | 21.6% | 1.79 (0.86–2.99) |
| + Dementia | 3.7% | 26.5% | 7.11 (2.05–14.18) | 3.3% | 8.0% | 2.42 (0.00–5.23) |
| Dementia |  |  |  |  |  |  |
| + Cardiovascular | 70.4% | 26.5% | 0.38 (0.00–0.83) | 70.9% | 100.0% | 1.41 (1.38–1.44) |
| + Musculoskeletal | 40.5% | 26.5% | 0.65 (0.00–1.45) | 58.8% | 62.3% | 1.06 (0.48–1.69) |
| + Mental disorder | 58.3% | 86.8% | 1.49 (0.97–1.75) | 69.2% | 86.8% | 1.25 (0.82–1.47) |
| + Diabetes | 23.0% | 13.2% | 0.58 (0.00–1.89) | 22.6% | 26.5% | 1.17 (0.00–2.61) |
| + Respiratory | 20.1% | 0.0% | 0.00 (0.00–0.00) | 20.4% | 37.7% | 1.85 (0.00–3.56) |
| + Cancer | 10.2% | 0.0% | 0.00 (0.00–0.00) | 10.8% | 0.0% | 0.00 (0.00–0.00) |
| + Stomach ulcer | 15.3% | 13.2% | 0.87 (0.00–2.85) | 15.0% | 35.8% | 2.39 (0.00–4.86) |
| **Eastern Europe** |  |  |  |  |  |  |
| Cardiovascular |  |  |  |  |  |  |
| + Musculoskeletal | 29.9% | 35.2% | 1.18 (1.12–1.24) | 48.5% | 52.8% | 1.09 (1.06–1.12) |
| + Mental disorder | 19.0% | 19.0% | 1.00 (0.92–1.08) | 33.8% | 35.5% | 1.05 (1.01–1.09) |
| + Diabetes | 21.9% | 21.5% | 0.98 (0.91–1.05) | 19.0% | 25.8% | 1.36 (1.28–1.43) |
| + Respiratory | 11.7% | 12.5% | 1.07 (0.97–1.19) | 12.3% | 16.6% | 1.35 (1.26–1.44) |
| + Cancer | 7.4% | 5.7% | 0.77 (0.66–0.90) | 8.8% | 9.4% | 1.07 (0.97–1.17) |
| + Stomach ulcer | 9.1% | 16.2% | 1.77 (1.61–1.93) | 9.1% | 17.3% | 1.91 (1.77–2.04) |
| + Dementia | 2.5% | 2.4% | 0.95 (0.73–1.19) | 2.3% | 2.4% | 1.02 (0.83–1.22) |
| Musculoskeletal |  |  |  |  |  |  |
| + Cardiovascular | 64.6% | 70.0% | 1.08 (1.05–1.12) | 59.2% | 72.5% | 1.22 (1.20–1.25) |
| + Mental disorder | 25.5% | 23.6% | 0.93 (0.83–1.02) | 39.8% | 41.9% | 1.05 (1.01–1.10) |
| + Diabetes | 19.5% | 20.5% | 1.05 (0.93–1.17) | 15.4% | 24.1% | 1.57 (1.48–1.67) |
| + Respiratory | 15.7% | 15.9% | 1.01 (0.89–1.14) | 14.3% | 18.8% | 1.31 (1.22–1.41) |
| + Cancer | 8.1% | 5.2% | 0.64 (0.49–0.81) | 9.7% | 9.8% | 1.01 (0.90–1.13) |
| + Stomach ulcer | 12.6% | 18.8% | 1.49 (1.32–1.66) | 11.2% | 20.4% | 1.82 (1.69–1.96) |
| + Dementia | 3.0% | 2.4% | 0.81 (0.56–1.09) | 2.4% | 2.7% | 1.16 (0.91–1.41) |
| Mental disorder |  |  |  |  |  |  |
| + Cardiovascular | 65.9% | 79.6% | 1.21 (1.16–1.26) | 60.7% | 73.6% | 1.21 (1.18–1.24) |
| + Musculoskeletal | 40.7% | 49.8% | 1.23 (1.14–1.32) | 58.3% | 63.2% | 1.08 (1.05–1.12) |
| + Diabetes | 21.3% | 21.6% | 1.01 (0.85–1.17) | 17.0% | 28.8% | 1.70 (1.58–1.83) |
| + Respiratory | 18.1% | 18.5% | 1.02 (0.86–1.19) | 15.7% | 23.1% | 1.47 (1.34–1.60) |
| + Cancer | 10.0% | 8.4% | 0.84 (0.63–1.07) | 10.9% | 11.3% | 1.04 (0.91–1.17) |
| + Stomach ulcer | 15.6% | 25.5% | 1.64 (1.42–1.87) | 13.1% | 21.8% | 1.66 (1.51–1.81) |
| + Dementia | 6.5% | 6.0% | 0.92 (0.64–1.21) | 4.0% | 4.4% | 1.10 (0.87–1.36) |
| Diabetes |  |  |  |  |  |  |
| + Cardiovascular | 79.1% | 86.3% | 1.09 (1.06–1.12) | 80.1% | 89.0% | 1.11 (1.09–1.13) |
| + Musculoskeletal | 32.3% | 41.4% | 1.28 (1.17–1.40) | 52.6% | 60.2% | 1.14 (1.09–1.20) |
| + Mental disorder | 21.9% | 20.8% | 0.95 (0.82–1.08) | 39.6% | 48.4% | 1.22 (1.15–1.29) |
| + Respiratory | 13.7% | 17.4% | 1.27 (1.08–1.50) | 16.0% | 23.8% | 1.49 (1.34–1.63) |
| + Cancer | 7.9% | 8.1% | 1.02 (0.78–1.30) | 9.8% | 10.2% | 1.04 (0.88–1.24) |
| + Stomach ulcer | 9.3% | 17.5% | 1.88 (1.57–2.18) | 10.7% | 18.9% | 1.76 (1.57–1.96) |
| + Dementia | 2.9% | 3.1% | 1.08 (0.65–1.54) | 3.1% | 2.6% | 0.84 (0.56–1.13) |
| Respiratory |  |  |  |  |  |  |
| + Cardiovascular | 67.1% | 72.8% | 1.09 (1.02–1.15) | 65.4% | 74.8% | 1.14 (1.10–1.19) |
| + Musculoskeletal | 41.7% | 46.9% | 1.12 (1.01–1.23) | 62.3% | 62.3% | 1.00 (0.95–1.05) |
| + Mental disorder | 30.2% | 25.8% | 0.85 (0.73–0.99) | 47.0% | 50.5% | 1.07 (1.00–1.14) |
| + Diabetes | 21.9% | 25.3% | 1.15 (0.99–1.33) | 20.2% | 30.2% | 1.50 (1.35–1.65) |
| + Cancer | 10.0% | 7.8% | 0.78 (0.54–1.00) | 11.2% | 13.0% | 1.17 (0.96–1.38) |
| + Stomach ulcer | 15.4% | 22.0% | 1.43 (1.18–1.69) | 14.3% | 25.0% | 1.75 (1.56–1.95) |
| + Dementia | 3.9% | 3.6% | 0.92 (0.52–1.41) | 3.4% | 4.3% | 1.26 (0.88–1.68) |
| Cancer |  |  |  |  |  |  |
| + Cardiovascular | 62.0% | 56.0% | 0.90 (0.81–1.00) | 56.0% | 75.4% | 1.35 (1.28–1.42) |
| + Musculoskeletal | 31.3% | 25.5% | 0.81 (0.65–0.98) | 50.4% | 57.7% | 1.15 (1.06–1.23) |
| + Mental disorder | 24.7% | 20.5% | 0.83 (0.65–1.03) | 38.7% | 43.9% | 1.13 (1.03–1.25) |
| + Diabetes | 18.7% | 21.0% | 1.12 (0.87–1.40) | 15.1% | 24.8% | 1.65 (1.42–1.91) |
| + Respiratory | 15.0% | 13.4% | 0.89 (0.64–1.16) | 13.2% | 23.2% | 1.75 (1.47–2.03) |
| + Stomach ulcer | 11.6% | 26.0% | 2.24 (1.77–2.71) | 11.0% | 18.1% | 1.64 (1.34–1.96) |
| + Dementia | 3.0% | 3.7% | 1.25 (0.55–2.03) | 2.3% | 2.6% | 1.15 (0.60–1.74) |
| Stomach ulcer |  |  |  |  |  |  |
| + Cardiovascular | 64.9% | 65.2% | 1.00 (0.95–1.07) | 63.9% | 82.3% | 1.29 (1.25–1.33) |
| + Musculoskeletal | 41.2% | 38.2% | 0.93 (0.83–1.03) | 64.5% | 70.3% | 1.09 (1.04–1.14) |
| + Mental disorder | 32.0% | 24.4% | 0.76 (0.66–0.87) | 51.4% | 49.8% | 0.97 (0.91–1.03) |
| + Diabetes | 18.5% | 16.9% | 0.91 (0.75–1.08) | 18.0% | 26.2% | 1.46 (1.30–1.64) |
| + Respiratory | 19.1% | 15.5% | 0.82 (0.66–0.98) | 18.8% | 26.1% | 1.39 (1.24–1.55) |
| + Cancer | 9.9% | 9.1% | 0.92 (0.69–1.16) | 12.1% | 10.5% | 0.86 (0.71–1.03) |
| + Dementia | 3.7% | 3.6% | 0.97 (0.59–1.40) | 3.3% | 3.3% | 1.00 (0.65–1.39) |
| Dementia |  |  |  |  |  |  |
| + Cardiovascular | 70.4% | 66.8% | 0.95 (0.82–1.07) | 70.9% | 80.7% | 1.14 (1.05–1.23) |
| + Musculoskeletal | 40.5% | 46.9% | 1.16 (0.91–1.39) | 58.8% | 66.6% | 1.13 (1.01–1.27) |
| + Mental disorder | 58.3% | 60.6% | 1.04 (0.88–1.19) | 69.2% | 69.0% | 1.00 (0.89–1.10) |
| + Diabetes | 23.0% | 16.0% | 0.70 (0.44–1.00) | 22.6% | 23.4% | 1.04 (0.76–1.35) |
| + Respiratory | 20.1% | 25.5% | 1.27 (0.86–1.71) | 20.4% | 31.3% | 1.53 (1.17–1.89) |
| + Cancer | 10.2% | 10.9% | 1.06 (0.50–1.76) | 10.8% | 8.3% | 0.77 (0.40–1.23) |
| + Stomach ulcer | 15.3% | 33.3% | 2.18 (1.58–2.81) | 15.0% | 22.3% | 1.49 (1.07–1.94) |
| **Other Europe & North America** |  |  |  |  |  |  |
| Cardiovascular |  |  |  |  |  |  |
| + Musculoskeletal | 29.9% | 31.3% | 1.05 (1.00–1.10) | 48.5% | 51.7% | 1.06 (1.03–1.10) |
| + Mental disorder | 19.0% | 21.6% | 1.14 (1.07–1.21) | 33.8% | 36.4% | 1.08 (1.04–1.12) |
| + Diabetes | 21.9% | 25.2% | 1.15 (1.08–1.21) | 19.0% | 19.0% | 1.00 (0.93–1.06) |
| + Respiratory | 11.7% | 13.5% | 1.15 (1.06–1.25) | 12.3% | 15.4% | 1.26 (1.17–1.35) |
| + Cancer | 7.4% | 9.1% | 1.23 (1.11–1.36) | 8.8% | 10.0% | 1.14 (1.03–1.24) |
| + Stomach ulcer | 9.1% | 9.4% | 1.03 (0.92–1.13) | 9.1% | 11.7% | 1.28 (1.18–1.40) |
| + Dementia | 2.5% | 2.9% | 1.18 (0.98–1.41) | 2.3% | 2.3% | 0.96 (0.78–1.15) |
| Musculoskeletal |  |  |  |  |  |  |
| + Cardiovascular | 64.6% | 61.7% | 0.96 (0.92–0.99) | 59.2% | 59.4% | 1.00 (0.97–1.03) |
| + Mental disorder | 25.5% | 28.8% | 1.13 (1.05–1.21) | 39.8% | 43.4% | 1.09 (1.05–1.13) |
| + Diabetes | 19.5% | 23.3% | 1.20 (1.08–1.30) | 15.4% | 14.5% | 0.95 (0.87–1.02) |
| + Respiratory | 15.7% | 16.7% | 1.06 (0.96–1.18) | 14.3% | 17.2% | 1.21 (1.12–1.29) |
| + Cancer | 8.1% | 9.6% | 1.18 (1.03–1.37) | 9.7% | 10.8% | 1.12 (1.01–1.22) |
| + Stomach ulcer | 12.6% | 14.0% | 1.11 (0.98–1.24) | 11.2% | 14.2% | 1.26 (1.17–1.36) |
| + Dementia | 3.0% | 3.1% | 1.04 (0.79–1.33) | 2.4% | 2.4% | 1.03 (0.82–1.25) |
| Mental disorder |  |  |  |  |  |  |
| + Cardiovascular | 65.9% | 62.2% | 0.94 (0.90–0.99) | 60.7% | 61.3% | 1.01 (0.98–1.04) |
| + Musculoskeletal | 40.7% | 42.1% | 1.04 (0.96–1.10) | 58.3% | 63.0% | 1.08 (1.05–1.11) |
| + Diabetes | 21.3% | 28.8% | 1.35 (1.22–1.47) | 17.0% | 16.3% | 0.96 (0.88–1.04) |
| + Respiratory | 18.1% | 19.9% | 1.10 (0.97–1.22) | 15.7% | 19.2% | 1.22 (1.12–1.32) |
| + Cancer | 10.0% | 10.1% | 1.01 (0.83–1.18) | 10.9% | 11.6% | 1.06 (0.95–1.17) |
| + Stomach ulcer | 15.6% | 18.8% | 1.21 (1.06–1.34) | 13.1% | 18.6% | 1.42 (1.31–1.55) |
| + Dementia | 6.5% | 8.0% | 1.22 (0.98–1.49) | 4.0% | 3.8% | 0.96 (0.77–1.16) |
| Diabetes |  |  |  |  |  |  |
| + Cardiovascular | 79.1% | 73.1% | 0.92 (0.89–0.96) | 80.1% | 81.0% | 1.01 (0.98–1.04) |
| + Musculoskeletal | 32.3% | 34.2% | 1.06 (0.97–1.14) | 52.6% | 53.6% | 1.02 (0.96–1.08) |
| + Mental disorder | 21.9% | 29.1% | 1.33 (1.20–1.45) | 39.6% | 40.9% | 1.03 (0.95–1.10) |
| + Respiratory | 13.7% | 12.9% | 0.94 (0.81–1.08) | 16.0% | 20.5% | 1.28 (1.13–1.42) |
| + Cancer | 7.9% | 9.3% | 1.18 (0.98–1.40) | 9.8% | 9.1% | 0.93 (0.77–1.10) |
| + Stomach ulcer | 9.3% | 12.4% | 1.33 (1.14–1.53) | 10.7% | 14.8% | 1.38 (1.18–1.60) |
| + Dementia | 2.9% | 5.0% | 1.71 (1.30–2.15) | 3.1% | 3.6% | 1.16 (0.82–1.52) |
| Respiratory |  |  |  |  |  |  |
| + Cardiovascular | 67.1% | 66.7% | 1.00 (0.94–1.04) | 65.4% | 68.4% | 1.05 (1.00–1.09) |
| + Musculoskeletal | 41.7% | 42.1% | 1.01 (0.92–1.09) | 62.3% | 66.6% | 1.07 (1.02–1.12) |
| + Mental disorder | 30.2% | 35.2% | 1.16 (1.04–1.28) | 47.0% | 51.2% | 1.09 (1.02–1.16) |
| + Diabetes | 21.9% | 22.0% | 1.00 (0.87–1.15) | 20.2% | 20.6% | 1.02 (0.89–1.16) |
| + Cancer | 10.0% | 14.0% | 1.39 (1.15–1.66) | 11.2% | 15.3% | 1.37 (1.15–1.58) |
| + Stomach ulcer | 15.4% | 13.7% | 0.89 (0.75–1.05) | 14.3% | 19.0% | 1.33 (1.15–1.50) |
| + Dementia | 3.9% | 3.3% | 0.86 (0.53–1.21) | 3.4% | 2.9% | 0.87 (0.58–1.24) |
| Cancer |  |  |  |  |  |  |
| + Cardiovascular | 62.0% | 64.0% | 1.03 (0.96–1.10) | 56.0% | 56.9% | 1.02 (0.95–1.07) |
| + Musculoskeletal | 31.3% | 35.2% | 1.12 (0.99–1.26) | 50.4% | 53.5% | 1.06 (0.99–1.13) |
| + Mental disorder | 24.7% | 25.1% | 1.02 (0.86–1.18) | 38.7% | 39.5% | 1.02 (0.93–1.12) |
| + Diabetes | 18.7% | 23.0% | 1.23 (1.04–1.43) | 15.1% | 12.3% | 0.82 (0.66–0.98) |
| + Respiratory | 15.0% | 19.6% | 1.30 (1.09–1.53) | 13.2% | 19.0% | 1.44 (1.22–1.65) |
| + Stomach ulcer | 11.6% | 13.2% | 1.13 (0.89–1.40) | 11.0% | 16.9% | 1.54 (1.30–1.80) |
| + Dementia | 3.0% | 2.8% | 0.94 (0.51–1.48) | 2.3% | 3.1% | 1.34 (0.81–1.94) |
| Stomach ulcer |  |  |  |  |  |  |
| + Cardiovascular | 64.9% | 57.7% | 0.89 (0.82–0.96) | 63.9% | 63.4% | 0.99 (0.94–1.04) |
| + Musculoskeletal | 41.2% | 43.5% | 1.06 (0.97–1.16) | 64.5% | 67.0% | 1.04 (0.99–1.09) |
| + Mental disorder | 32.0% | 40.6% | 1.27 (1.14–1.40) | 51.4% | 60.2% | 1.17 (1.11–1.24) |
| + Diabetes | 18.5% | 26.1% | 1.41 (1.21–1.63) | 18.0% | 18.7% | 1.04 (0.89–1.20) |
| + Respiratory | 19.1% | 17.2% | 0.90 (0.75–1.07) | 18.8% | 23.1% | 1.23 (1.07–1.40) |
| + Cancer | 9.9% | 13.2% | 1.33 (1.04–1.63) | 12.1% | 15.8% | 1.30 (1.09–1.54) |
| + Dementia | 3.7% | 4.0% | 1.07 (0.67–1.51) | 3.3% | 1.9% | 0.58 (0.32–0.91) |
| Dementia |  |  |  |  |  |  |
| + Cardiovascular | 70.4% | 62.4% | 0.89 (0.78–0.99) | 70.9% | 65.9% | 0.93 (0.82–1.03) |
| + Musculoskeletal | 40.5% | 34.7% | 0.86 (0.69–1.05) | 58.8% | 62.8% | 1.07 (0.94–1.20) |
| + Mental disorder | 58.3% | 62.9% | 1.08 (0.95–1.21) | 69.2% | 68.5% | 0.99 (0.88–1.09) |
| + Diabetes | 23.0% | 40.6% | 1.77 (1.46–2.13) | 22.6% | 25.0% | 1.11 (0.79–1.41) |
| + Respiratory | 20.1% | 14.5% | 0.72 (0.48–0.99) | 20.4% | 20.5% | 1.00 (0.71–1.31) |
| + Cancer | 10.2% | 8.4% | 0.82 (0.46–1.24) | 10.8% | 16.4% | 1.52 (0.99–2.17) |
| + Stomach ulcer | 15.3% | 17.4% | 1.14 (0.80–1.52) | 15.0% | 9.2% | 0.62 (0.33–0.94) |

*Notes:* Values are age-standardized using the 2013 European standard population.

## **Table S7.** Prevalence and relative risk of chronic disease combinations for immigrants versus native-born individuals by gender and region of residence group

|  | **Men** | | | **Women** | | |
| --- | --- | --- | --- | --- | --- | --- |
|  | **Native-born** | **Immigrant** | **RR (95% CI)** | **Native-born** | **Immigrant** | **RR (95% CI)** |
| **Northern Europe** |  |  |  |  |  |  |
| Cardiovascular |  |  |  |  |  |  |
| + Musculoskeletal | 31.5% | 27.1% | 0.86 (0.74–0.99) | 44.9% | 48.5% | 1.08 (0.99–1.16) |
| + Mental disorder | 16.1% | 24.5% | 1.52 (1.27–1.77) | 27.2% | 31.9% | 1.17 (1.04–1.31) |
| + Diabetes | 18.6% | 26.6% | 1.43 (1.21–1.65) | 14.9% | 16.8% | 1.13 (0.93–1.33) |
| + Respiratory | 11.7% | 10.1% | 0.86 (0.65–1.11) | 14.4% | 21.9% | 1.52 (1.30–1.75) |
| + Cancer | 8.7% | 9.2% | 1.06 (0.77–1.37) | 10.2% | 10.4% | 1.02 (0.78–1.28) |
| + Stomach ulcer | 7.1% | 12.6% | 1.78 (1.37–2.24) | 6.2% | 10.3% | 1.68 (1.29–2.11) |
| + Dementia | 1.9% | 4.8% | 2.57 (1.54–3.65) | 1.5% | 2.4% | 1.63 (0.87–2.51) |
| Musculoskeletal |  |  |  |  |  |  |
| + Cardiovascular | 58.7% | 55.6% | 0.95 (0.83–1.06) | 48.3% | 58.4% | 1.21 (1.12–1.30) |
| + Mental disorder | 17.5% | 16.6% | 0.95 (0.67–1.23) | 30.7% | 41.8% | 1.36 (1.23–1.50) |
| + Diabetes | 14.6% | 18.4% | 1.26 (0.91–1.64) | 9.8% | 9.0% | 0.92 (0.68–1.17) |
| + Respiratory | 14.5% | 8.7% | 0.60 (0.36–0.87) | 16.7% | 24.2% | 1.45 (1.24–1.67) |
| + Cancer | 8.8% | 13.9% | 1.58 (1.04–2.12) | 11.6% | 10.0% | 0.86 (0.63–1.08) |
| + Stomach ulcer | 7.6% | 14.6% | 1.94 (1.31–2.61) | 8.0% | 12.7% | 1.59 (1.23–1.96) |
| + Dementia | 2.0% | 2.3% | 1.11 (0.32–2.26) | 1.3% | 2.0% | 1.51 (0.72–2.56) |
| Mental disorder |  |  |  |  |  |  |
| + Cardiovascular | 57.7% | 66.1% | 1.14 (1.02–1.27) | 50.8% | 56.4% | 1.11 (1.01–1.21) |
| + Musculoskeletal | 33.8% | 24.6% | 0.73 (0.53–0.95) | 52.9% | 59.2% | 1.12 (1.02–1.22) |
| + Diabetes | 16.4% | 33.1% | 2.02 (1.57–2.51) | 11.5% | 11.7% | 1.02 (0.75–1.33) |
| + Respiratory | 18.4% | 5.4% | 0.29 (0.13–0.49) | 18.3% | 19.0% | 1.04 (0.81–1.28) |
| + Cancer | 7.8% | 10.2% | 1.31 (0.73–1.94) | 11.6% | 9.6% | 0.82 (0.57–1.09) |
| + Stomach ulcer | 11.0% | 22.9% | 2.09 (1.55–2.75) | 9.8% | 18.6% | 1.90 (1.48–2.32) |
| + Dementia | 4.8% | 8.3% | 1.74 (0.93–2.76) | 2.3% | 2.4% | 1.01 (0.38–1.74) |
| Diabetes |  |  |  |  |  |  |
| + Cardiovascular | 79.3% | 83.6% | 1.05 (0.98–1.14) | 79.0% | 89.6% | 1.13 (1.05–1.20) |
| + Musculoskeletal | 33.4% | 28.7% | 0.86 (0.64–1.08) | 48.2% | 40.6% | 0.84 (0.66–1.02) |
| + Mental disorder | 19.7% | 38.7% | 1.96 (1.56–2.39) | 32.6% | 36.6% | 1.12 (0.87–1.42) |
| + Respiratory | 12.6% | 3.5% | 0.28 (0.06–0.53) | 21.3% | 18.8% | 0.88 (0.56–1.25) |
| + Cancer | 8.3% | 6.9% | 0.82 (0.37–1.35) | 11.9% | 5.6% | 0.47 (0.15–0.87) |
| + Stomach ulcer | 7.9% | 22.0% | 2.77 (1.87–3.78) | 9.5% | 10.6% | 1.12 (0.59–1.79) |
| + Dementia | 1.9% | 4.6% | 2.43 (0.78–4.78) | 1.9% | 2.5% | 1.27 (0.00–3.25) |
| Respiratory |  |  |  |  |  |  |
| + Cardiovascular | 58.6% | 61.7% | 1.05 (0.87–1.23) | 55.0% | 69.3% | 1.26 (1.14–1.38) |
| + Musculoskeletal | 39.0% | 26.1% | 0.67 (0.42–0.94) | 58.9% | 63.5% | 1.08 (0.96–1.20) |
| + Mental disorder | 25.7% | 10.5% | 0.41 (0.15–0.67) | 37.5% | 35.4% | 0.94 (0.75–1.12) |
| + Diabetes | 14.8% | 6.2% | 0.42 (0.09–0.82) | 15.3% | 11.0% | 0.72 (0.43–1.05) |
| + Cancer | 8.4% | 6.3% | 0.76 (0.16–1.47) | 12.1% | 19.9% | 1.64 (1.20–2.17) |
| + Stomach ulcer | 13.0% | 9.6% | 0.74 (0.29–1.30) | 9.7% | 9.7% | 1.00 (0.57–1.47) |
| + Dementia | 1.9% | 0.9% | 0.45 (0.00–1.70) | 2.4% | 1.8% | 0.76 (0.19–1.72) |
| Cancer |  |  |  |  |  |  |
| + Cardiovascular | 58.8% | 59.4% | 1.01 (0.82–1.20) | 43.4% | 59.0% | 1.36 (1.17–1.58) |
| + Musculoskeletal | 31.7% | 45.2% | 1.43 (1.08–1.80) | 45.5% | 44.4% | 0.98 (0.78–1.17) |
| + Mental disorder | 13.7% | 23.6% | 1.72 (1.04–2.48) | 26.1% | 24.3% | 0.93 (0.64–1.23) |
| + Diabetes | 13.6% | 14.7% | 1.09 (0.56–1.75) | 9.8% | 5.7% | 0.59 (0.23–1.07) |
| + Respiratory | 10.6% | 5.3% | 0.50 (0.11–1.02) | 13.4% | 34.9% | 2.61 (1.93–3.32) |
| + Stomach ulcer | 9.7% | 15.1% | 1.56 (0.82–2.43) | 5.1% | 9.2% | 1.81 (0.87–3.00) |
| + Dementia | 1.8% | 3.3% | 1.86 (0.00–5.08) | 1.3% | 3.0% | 2.31 (0.51–5.50) |
| Stomach ulcer |  |  |  |  |  |  |
| + Cardiovascular | 64.8% | 62.0% | 0.96 (0.79–1.13) | 57.1% | 65.0% | 1.14 (0.97–1.31) |
| + Musculoskeletal | 37.5% | 36.4% | 0.97 (0.70–1.29) | 69.0% | 70.5% | 1.02 (0.89–1.16) |
| + Mental disorder | 28.4% | 47.6% | 1.68 (1.30–2.14) | 49.3% | 70.9% | 1.44 (1.22–1.65) |
| + Diabetes | 17.1% | 33.2% | 1.94 (1.36–2.61) | 16.5% | 14.7% | 0.89 (0.48–1.39) |
| + Respiratory | 24.1% | 13.3% | 0.55 (0.27–0.88) | 23.7% | 21.2% | 0.89 (0.55–1.29) |
| + Cancer | 12.7% | 14.8% | 1.16 (0.58–1.83) | 11.2% | 14.1% | 1.26 (0.64–1.95) |
| + Dementia | 3.0% | 1.2% | 0.40 (0.00–1.42) | 3.3% | 3.3% | 1.01 (0.00–2.49) |
| Dementia |  |  |  |  |  |  |
| + Cardiovascular | 70.4% | 66.9% | 0.95 (0.71–1.17) | 62.5% | 78.7% | 1.26 (0.92–1.57) |
| + Musculoskeletal | 35.9% | 12.4% | 0.35 (0.08–0.68) | 49.1% | 53.4% | 1.09 (0.63–1.58) |
| + Mental disorder | 49.7% | 44.7% | 0.90 (0.58–1.23) | 50.0% | 43.5% | 0.87 (0.42–1.33) |
| + Diabetes | 17.4% | 14.3% | 0.82 (0.27–1.54) | 14.4% | 14.6% | 1.01 (0.00–2.30) |
| + Respiratory | 12.0% | 2.1% | 0.18 (0.00–0.61) | 27.4% | 17.9% | 0.65 (0.16–1.42) |
| + Cancer | 9.6% | 5.6% | 0.58 (0.00–1.61) | 12.4% | 17.9% | 1.45 (0.33–3.22) |
| + Stomach ulcer | 13.1% | 2.1% | 0.16 (0.00–0.66) | 15.3% | 17.7% | 1.16 (0.26–2.38) |
| **Western Europe** |  |  |  |  |  |  |
| Cardiovascular |  |  |  |  |  |  |
| + Musculoskeletal | 32.2% | 35.9% | 1.11 (1.06–1.16) | 51.3% | 57.3% | 1.12 (1.08–1.15) |
| + Mental disorder | 23.4% | 23.2% | 0.99 (0.93–1.06) | 39.9% | 41.6% | 1.04 (1.00–1.09) |
| + Diabetes | 21.0% | 26.4% | 1.25 (1.18–1.33) | 16.5% | 22.9% | 1.39 (1.30–1.47) |
| + Respiratory | 13.1% | 15.0% | 1.15 (1.05–1.25) | 14.0% | 16.6% | 1.18 (1.09–1.28) |
| + Cancer | 9.0% | 8.5% | 0.94 (0.84–1.05) | 10.4% | 10.6% | 1.02 (0.92–1.11) |
| + Stomach ulcer | 8.5% | 9.9% | 1.17 (1.04–1.31) | 8.9% | 12.3% | 1.39 (1.26–1.52) |
| + Dementia | 2.6% | 2.2% | 0.85 (0.66–1.04) | 2.1% | 2.7% | 1.28 (1.04–1.53) |
| Musculoskeletal |  |  |  |  |  |  |
| + Cardiovascular | 61.4% | 62.3% | 1.01 (0.98–1.05) | 52.9% | 57.9% | 1.10 (1.06–1.13) |
| + Mental disorder | 30.5% | 27.1% | 0.89 (0.83–0.96) | 45.4% | 44.7% | 0.98 (0.95–1.02) |
| + Diabetes | 18.0% | 24.9% | 1.38 (1.27–1.50) | 12.2% | 17.3% | 1.42 (1.31–1.52) |
| + Respiratory | 16.8% | 16.5% | 0.98 (0.88–1.09) | 15.1% | 17.1% | 1.13 (1.05–1.21) |
| + Cancer | 9.7% | 8.8% | 0.91 (0.77–1.06) | 11.1% | 11.3% | 1.02 (0.92–1.10) |
| + Stomach ulcer | 11.8% | 14.3% | 1.21 (1.07–1.35) | 10.3% | 15.2% | 1.47 (1.36–1.60) |
| + Dementia | 2.8% | 1.9% | 0.68 (0.48–0.92) | 2.1% | 2.6% | 1.27 (1.02–1.54) |
| Mental disorder |  |  |  |  |  |  |
| + Cardiovascular | 61.7% | 63.2% | 1.02 (0.97–1.07) | 54.3% | 59.9% | 1.10 (1.07–1.14) |
| + Musculoskeletal | 42.2% | 43.9% | 1.04 (0.97–1.11) | 59.9% | 63.6% | 1.06 (1.03–1.09) |
| + Diabetes | 19.2% | 28.4% | 1.48 (1.33–1.64) | 13.5% | 18.9% | 1.40 (1.28–1.52) |
| + Respiratory | 18.4% | 21.9% | 1.19 (1.05–1.32) | 16.5% | 20.7% | 1.26 (1.16–1.36) |
| + Cancer | 11.3% | 11.2% | 0.99 (0.82–1.16) | 12.0% | 12.6% | 1.05 (0.95–1.17) |
| + Stomach ulcer | 13.9% | 19.6% | 1.41 (1.22–1.60) | 12.0% | 17.6% | 1.47 (1.34–1.61) |
| + Dementia | 5.4% | 4.8% | 0.90 (0.66–1.15) | 3.0% | 4.2% | 1.41 (1.13–1.68) |
| Diabetes |  |  |  |  |  |  |
| + Cardiovascular | 78.6% | 72.1% | 0.92 (0.88–0.95) | 75.8% | 79.4% | 1.05 (1.01–1.08) |
| + Musculoskeletal | 35.2% | 39.4% | 1.12 (1.04–1.21) | 54.4% | 59.2% | 1.09 (1.03–1.15) |
| + Mental disorder | 27.0% | 28.2% | 1.04 (0.95–1.14) | 46.0% | 45.8% | 1.00 (0.93–1.07) |
| + Respiratory | 15.6% | 14.5% | 0.93 (0.80–1.07) | 18.2% | 20.4% | 1.12 (0.98–1.26) |
| + Cancer | 9.9% | 7.9% | 0.80 (0.64–0.97) | 11.6% | 12.4% | 1.06 (0.90–1.24) |
| + Stomach ulcer | 9.0% | 13.2% | 1.46 (1.23–1.70) | 11.3% | 13.9% | 1.23 (1.03–1.44) |
| + Dementia | 2.7% | 3.4% | 1.23 (0.85–1.65) | 2.4% | 3.4% | 1.37 (0.94–1.91) |
| Respiratory |  |  |  |  |  |  |
| + Cardiovascular | 64.8% | 68.1% | 1.05 (1.00–1.10) | 60.3% | 66.2% | 1.10 (1.04–1.15) |
| + Musculoskeletal | 43.7% | 43.1% | 0.99 (0.90–1.08) | 63.2% | 67.8% | 1.07 (1.02–1.12) |
| + Mental disorder | 34.7% | 37.0% | 1.07 (0.96–1.18) | 52.3% | 57.8% | 1.10 (1.04–1.17) |
| + Diabetes | 20.8% | 24.0% | 1.15 (1.00–1.31) | 16.8% | 23.2% | 1.38 (1.22–1.57) |
| + Cancer | 11.9% | 13.5% | 1.13 (0.92–1.36) | 12.2% | 13.6% | 1.12 (0.94–1.31) |
| + Stomach ulcer | 14.6% | 15.8% | 1.08 (0.90–1.28) | 12.5% | 19.3% | 1.54 (1.34–1.76) |
| + Dementia | 4.6% | 2.4% | 0.52 (0.30–0.78) | 2.8% | 3.4% | 1.21 (0.78–1.67) |
| Cancer |  |  |  |  |  |  |
| + Cardiovascular | 59.9% | 61.8% | 1.03 (0.96–1.11) | 51.6% | 54.9% | 1.07 (0.99–1.14) |
| + Musculoskeletal | 33.4% | 36.6% | 1.10 (0.96–1.23) | 53.2% | 58.1% | 1.09 (1.02–1.16) |
| + Mental disorder | 29.4% | 29.9% | 1.02 (0.88–1.16) | 43.2% | 45.4% | 1.05 (0.97–1.14) |
| + Diabetes | 18.1% | 21.2% | 1.17 (0.97–1.39) | 12.6% | 18.5% | 1.47 (1.24–1.72) |
| + Respiratory | 16.5% | 22.0% | 1.34 (1.09–1.58) | 13.9% | 17.1% | 1.23 (1.02–1.46) |
| + Stomach ulcer | 10.6% | 13.8% | 1.31 (1.01–1.64) | 10.8% | 16.7% | 1.55 (1.29–1.85) |
| + Dementia | 2.7% | 2.1% | 0.78 (0.34–1.32) | 2.1% | 2.5% | 1.21 (0.73–1.85) |
| Stomach ulcer |  |  |  |  |  |  |
| + Cardiovascular | 62.9% | 60.4% | 0.96 (0.89–1.03) | 60.8% | 62.7% | 1.03 (0.97–1.09) |
| + Musculoskeletal | 45.8% | 49.2% | 1.07 (0.97–1.18) | 68.3% | 75.3% | 1.10 (1.05–1.15) |
| + Mental disorder | 39.3% | 42.9% | 1.09 (0.98–1.21) | 60.7% | 61.7% | 1.02 (0.96–1.08) |
| + Diabetes | 17.7% | 29.0% | 1.64 (1.41–1.92) | 16.7% | 20.3% | 1.22 (1.03–1.40) |
| + Respiratory | 21.8% | 21.6% | 0.99 (0.82–1.18) | 19.8% | 24.2% | 1.22 (1.04–1.39) |
| + Cancer | 11.9% | 13.4% | 1.13 (0.88–1.40) | 14.9% | 16.2% | 1.09 (0.91–1.30) |
| + Dementia | 3.9% | 4.4% | 1.13 (0.72–1.71) | 3.1% | 3.3% | 1.08 (0.67–1.58) |
| Dementia |  |  |  |  |  |  |
| + Cardiovascular | 67.4% | 70.1% | 1.04 (0.92–1.18) | 62.5% | 78.3% | 1.25 (1.14–1.38) |
| + Musculoskeletal | 39.3% | 34.1% | 0.87 (0.63–1.09) | 61.1% | 66.4% | 1.09 (0.96–1.21) |
| + Mental disorder | 57.3% | 68.4% | 1.19 (1.04–1.36) | 68.0% | 81.0% | 1.19 (1.09–1.29) |
| + Diabetes | 19.2% | 45.9% | 2.39 (1.87–2.99) | 16.7% | 27.9% | 1.67 (1.22–2.17) |
| + Respiratory | 25.2% | 15.9% | 0.63 (0.37–0.91) | 20.4% | 21.8% | 1.07 (0.76–1.41) |
| + Cancer | 10.4% | 8.7% | 0.84 (0.35–1.44) | 13.4% | 11.6% | 0.86 (0.51–1.26) |
| + Stomach ulcer | 16.0% | 26.0% | 1.62 (1.14–2.27) | 15.5% | 13.9% | 0.90 (0.56–1.29) |
| **Southern Europe** |  |  |  |  |  |  |
| Cardiovascular |  |  |  |  |  |  |
| + Musculoskeletal | 23.5% | 19.0% | 0.81 (0.71–0.90) | 48.6% | 42.6% | 0.88 (0.82–0.92) |
| + Mental disorder | 17.3% | 19.5% | 1.12 (1.00–1.24) | 34.8% | 28.7% | 0.82 (0.76–0.89) |
| + Diabetes | 23.3% | 26.4% | 1.13 (1.03–1.22) | 20.4% | 20.1% | 0.99 (0.89–1.08) |
| + Respiratory | 10.4% | 11.3% | 1.09 (0.93–1.26) | 10.0% | 10.7% | 1.07 (0.91–1.21) |
| + Cancer | 5.6% | 7.4% | 1.32 (1.07–1.61) | 8.0% | 8.6% | 1.08 (0.91–1.26) |
| + Stomach ulcer | 8.9% | 8.2% | 0.92 (0.76–1.08) | 9.3% | 10.9% | 1.17 (1.02–1.33) |
| + Dementia | 2.2% | 3.1% | 1.40 (1.00–1.87) | 2.7% | 2.2% | 0.82 (0.58–1.12) |
| Musculoskeletal |  |  |  |  |  |  |
| + Cardiovascular | 67.1% | 63.5% | 0.95 (0.88–1.01) | 60.7% | 65.0% | 1.07 (1.02–1.12) |
| + Mental disorder | 26.0% | 28.1% | 1.08 (0.93–1.25) | 40.0% | 37.9% | 0.95 (0.87–1.01) |
| + Diabetes | 22.4% | 21.7% | 0.97 (0.80–1.14) | 17.1% | 17.8% | 1.04 (0.90–1.17) |
| + Respiratory | 15.8% | 17.9% | 1.13 (0.90–1.37) | 11.8% | 13.5% | 1.15 (0.98–1.34) |
| + Cancer | 6.7% | 7.7% | 1.15 (0.78–1.57) | 7.9% | 7.4% | 0.93 (0.75–1.14) |
| + Stomach ulcer | 13.5% | 12.7% | 0.94 (0.72–1.17) | 12.0% | 14.3% | 1.19 (1.02–1.36) |
| + Dementia | 3.2% | 6.8% | 2.13 (1.40–2.98) | 2.7% | 2.5% | 0.89 (0.58–1.24) |
| Mental disorder |  |  |  |  |  |  |
| + Cardiovascular | 69.5% | 68.0% | 0.98 (0.90–1.05) | 63.7% | 64.4% | 1.01 (0.96–1.06) |
| + Musculoskeletal | 35.8% | 29.6% | 0.83 (0.70–0.95) | 58.6% | 55.2% | 0.94 (0.89–1.00) |
| + Diabetes | 24.4% | 28.4% | 1.16 (0.99–1.36) | 20.1% | 20.9% | 1.04 (0.89–1.19) |
| + Respiratory | 17.2% | 14.1% | 0.82 (0.62–1.04) | 13.0% | 15.9% | 1.22 (1.04–1.42) |
| + Cancer | 9.3% | 5.5% | 0.60 (0.36–0.88) | 9.6% | 9.8% | 1.01 (0.80–1.25) |
| + Stomach ulcer | 15.4% | 11.6% | 0.75 (0.55–0.96) | 13.5% | 16.2% | 1.21 (1.02–1.41) |
| + Dementia | 7.1% | 12.4% | 1.76 (1.28–2.29) | 4.8% | 5.5% | 1.14 (0.81–1.50) |
| Diabetes |  |  |  |  |  |  |
| + Cardiovascular | 75.4% | 73.9% | 0.98 (0.92–1.03) | 77.5% | 79.3% | 1.02 (0.97–1.07) |
| + Musculoskeletal | 25.2% | 18.1% | 0.72 (0.59–0.86) | 51.6% | 45.3% | 0.88 (0.79–0.97) |
| + Mental disorder | 19.5% | 22.8% | 1.17 (0.99–1.37) | 41.9% | 35.4% | 0.84 (0.75–0.95) |
| + Respiratory | 12.7% | 13.6% | 1.07 (0.84–1.31) | 12.9% | 11.4% | 0.88 (0.66–1.12) |
| + Cancer | 5.9% | 9.5% | 1.60 (1.16–2.07) | 7.8% | 8.6% | 1.10 (0.78–1.45) |
| + Stomach ulcer | 9.6% | 8.5% | 0.88 (0.63–1.17) | 9.9% | 13.9% | 1.41 (1.09–1.78) |
| + Dementia | 2.9% | 5.9% | 1.99 (1.33–2.84) | 4.0% | 2.4% | 0.60 (0.28–1.00) |
| Respiratory |  |  |  |  |  |  |
| + Cardiovascular | 68.3% | 73.6% | 1.08 (0.99–1.16) | 68.6% | 69.6% | 1.02 (0.93–1.09) |
| + Musculoskeletal | 36.2% | 35.6% | 0.98 (0.81–1.16) | 64.5% | 57.9% | 0.90 (0.81–0.99) |
| + Mental disorder | 28.7% | 26.8% | 0.94 (0.73–1.15) | 48.9% | 45.9% | 0.94 (0.82–1.06) |
| + Diabetes | 26.0% | 31.2% | 1.20 (0.98–1.45) | 23.2% | 18.6% | 0.80 (0.60–1.01) |
| + Cancer | 8.5% | 16.9% | 2.00 (1.41–2.64) | 9.8% | 15.1% | 1.54 (1.13–2.01) |
| + Stomach ulcer | 15.8% | 11.1% | 0.70 (0.46–0.98) | 16.8% | 21.3% | 1.27 (0.98–1.56) |
| + Dementia | 2.6% | 6.4% | 2.46 (1.34–4.01) | 4.4% | 5.6% | 1.26 (0.64–2.00) |
| Cancer |  |  |  |  |  |  |
| + Cardiovascular | 60.5% | 59.4% | 0.98 (0.85–1.10) | 59.8% | 67.5% | 1.13 (1.02–1.23) |
| + Musculoskeletal | 25.6% | 20.3% | 0.80 (0.56–1.06) | 47.6% | 40.0% | 0.84 (0.70–0.98) |
| + Mental disorder | 25.0% | 13.7% | 0.55 (0.35–0.76) | 39.1% | 34.4% | 0.88 (0.72–1.04) |
| + Diabetes | 19.8% | 27.4% | 1.39 (1.08–1.77) | 15.9% | 16.9% | 1.06 (0.76–1.43) |
| + Respiratory | 14.0% | 22.3% | 1.60 (1.17–2.09) | 10.7% | 17.9% | 1.67 (1.21–2.17) |
| + Stomach ulcer | 14.3% | 15.1% | 1.06 (0.70–1.43) | 11.0% | 16.6% | 1.51 (1.07–2.01) |
| + Dementia | 3.2% | 5.6% | 1.76 (0.76–3.01) | 2.9% | 4.6% | 1.61 (0.66–2.68) |
| Stomach ulcer |  |  |  |  |  |  |
| + Cardiovascular | 64.1% | 55.6% | 0.87 (0.76–0.98) | 61.9% | 66.4% | 1.07 (0.97–1.16) |
| + Musculoskeletal | 33.7% | 25.8% | 0.77 (0.58–0.94) | 63.3% | 57.9% | 0.91 (0.83–1.01) |
| + Mental disorder | 27.7% | 21.8% | 0.79 (0.59–1.00) | 48.4% | 44.1% | 0.91 (0.79–1.03) |
| + Diabetes | 21.2% | 20.8% | 0.98 (0.73–1.24) | 17.6% | 21.4% | 1.22 (0.94–1.49) |
| + Respiratory | 17.3% | 11.1% | 0.64 (0.42–0.89) | 16.1% | 20.1% | 1.25 (0.95–1.56) |
| + Cancer | 9.2% | 13.4% | 1.45 (0.98–1.98) | 9.8% | 13.2% | 1.35 (0.94–1.77) |
| + Dementia | 3.6% | 4.6% | 1.26 (0.50–2.16) | 3.4% | 1.3% | 0.38 (0.09–0.82) |
| Dementia |  |  |  |  |  |  |
| + Cardiovascular | 64.3% | 51.1% | 0.80 (0.64–0.97) | 74.4% | 47.9% | 0.64 (0.48–0.81) |
| + Musculoskeletal | 33.8% | 40.0% | 1.19 (0.87–1.52) | 60.2% | 43.3% | 0.72 (0.51–0.93) |
| + Mental disorder | 59.9% | 67.2% | 1.12 (0.94–1.30) | 73.4% | 70.1% | 0.95 (0.81–1.10) |
| + Diabetes | 26.2% | 41.6% | 1.59 (1.15–2.04) | 28.9% | 10.6% | 0.37 (0.11–0.63) |
| + Respiratory | 11.2% | 18.1% | 1.62 (0.89–2.47) | 19.8% | 32.8% | 1.65 (1.09–2.24) |
| + Cancer | 9.1% | 11.5% | 1.27 (0.60–2.08) | 10.4% | 18.8% | 1.81 (0.91–2.82) |
| + Stomach ulcer | 13.9% | 15.1% | 1.09 (0.58–1.70) | 14.5% | 4.2% | 0.29 (0.00–0.65) |
| **Eastern Europe** |  |  |  |  |  |  |
| Cardiovascular |  |  |  |  |  |  |
| + Musculoskeletal | 32.9% | 37.3% | 1.14 (1.08–1.21) | 46.8% | 53.0% | 1.13 (1.09–1.17) |
| + Mental disorder | 16.2% | 18.5% | 1.15 (1.04–1.25) | 29.3% | 35.3% | 1.21 (1.15–1.27) |
| + Diabetes | 22.8% | 21.1% | 0.92 (0.85–1.01) | 21.4% | 27.0% | 1.27 (1.19–1.34) |
| + Respiratory | 11.2% | 13.5% | 1.20 (1.06–1.34) | 11.9% | 15.8% | 1.33 (1.22–1.44) |
| + Cancer | 6.7% | 5.1% | 0.76 (0.61–0.93) | 7.6% | 9.3% | 1.22 (1.09–1.36) |
| + Stomach ulcer | 10.9% | 16.8% | 1.54 (1.39–1.71) | 9.9% | 19.4% | 1.96 (1.82–2.12) |
| + Dementia | 2.8% | 2.1% | 0.74 (0.53–0.98) | 2.5% | 2.4% | 0.95 (0.75–1.16) |
| Musculoskeletal |  |  |  |  |  |  |
| + Cardiovascular | 69.7% | 69.4% | 1.00 (0.95–1.04) | 70.9% | 74.5% | 1.05 (1.03–1.08) |
| + Mental disorder | 21.5% | 24.2% | 1.13 (1.00–1.26) | 35.3% | 42.4% | 1.20 (1.14–1.26) |
| + Diabetes | 21.5% | 19.7% | 0.92 (0.80–1.03) | 19.8% | 26.6% | 1.34 (1.25–1.44) |
| + Respiratory | 14.4% | 15.9% | 1.10 (0.95–1.26) | 14.7% | 17.9% | 1.22 (1.12–1.33) |
| + Cancer | 6.9% | 4.2% | 0.61 (0.44–0.80) | 8.8% | 9.7% | 1.10 (0.96–1.24) |
| + Stomach ulcer | 15.2% | 20.3% | 1.34 (1.16–1.51) | 13.1% | 22.5% | 1.71 (1.58–1.85) |
| + Dementia | 3.5% | 2.6% | 0.75 (0.48–1.06) | 2.7% | 2.7% | 0.98 (0.75–1.24) |
| Mental disorder |  |  |  |  |  |  |
| + Cardiovascular | 75.3% | 80.7% | 1.07 (1.02–1.13) | 72.1% | 78.0% | 1.08 (1.05–1.11) |
| + Musculoskeletal | 46.9% | 56.7% | 1.21 (1.11–1.30) | 57.2% | 66.2% | 1.16 (1.11–1.20) |
| + Diabetes | 24.2% | 21.3% | 0.88 (0.73–1.04) | 20.7% | 30.1% | 1.46 (1.33–1.58) |
| + Respiratory | 18.3% | 23.7% | 1.30 (1.07–1.51) | 16.5% | 22.6% | 1.37 (1.24–1.50) |
| + Cancer | 9.5% | 9.4% | 1.00 (0.70–1.29) | 10.6% | 11.8% | 1.11 (0.97–1.28) |
| + Stomach ulcer | 21.6% | 26.6% | 1.23 (1.03–1.40) | 15.9% | 25.6% | 1.61 (1.46–1.76) |
| + Dementia | 9.1% | 6.0% | 0.65 (0.42–0.89) | 5.2% | 4.1% | 0.77 (0.59–0.99) |
| Diabetes |  |  |  |  |  |  |
| + Cardiovascular | 83.6% | 88.2% | 1.06 (1.02–1.09) | 86.6% | 90.2% | 1.04 (1.02–1.06) |
| + Musculoskeletal | 36.6% | 43.8% | 1.20 (1.08–1.32) | 52.7% | 62.9% | 1.19 (1.13–1.25) |
| + Mental disorder | 19.0% | 20.4% | 1.08 (0.89–1.28) | 33.4% | 45.0% | 1.35 (1.25–1.44) |
| + Respiratory | 12.9% | 18.5% | 1.43 (1.18–1.71) | 15.6% | 22.3% | 1.43 (1.27–1.61) |
| + Cancer | 7.6% | 6.7% | 0.89 (0.63–1.19) | 9.6% | 9.5% | 0.98 (0.81–1.17) |
| + Stomach ulcer | 9.8% | 13.3% | 1.35 (1.05–1.67) | 11.2% | 22.5% | 2.00 (1.78–2.26) |
| + Dementia | 3.3% | 2.5% | 0.76 (0.39–1.17) | 3.0% | 3.6% | 1.20 (0.86–1.60) |
| Respiratory |  |  |  |  |  |  |
| + Cardiovascular | 74.1% | 68.9% | 0.93 (0.87–0.99) | 75.1% | 78.6% | 1.05 (1.01–1.09) |
| + Musculoskeletal | 44.8% | 44.0% | 0.98 (0.87–1.09) | 60.9% | 63.4% | 1.04 (0.98–1.10) |
| + Mental disorder | 26.3% | 28.2% | 1.07 (0.90–1.25) | 42.5% | 51.2% | 1.21 (1.11–1.30) |
| + Diabetes | 23.4% | 22.8% | 0.97 (0.80–1.15) | 24.2% | 32.4% | 1.34 (1.18–1.51) |
| + Cancer | 9.6% | 8.6% | 0.89 (0.61–1.23) | 10.3% | 12.1% | 1.17 (0.94–1.44) |
| + Stomach ulcer | 17.3% | 23.4% | 1.35 (1.10–1.60) | 17.4% | 29.4% | 1.69 (1.49–1.90) |
| + Dementia | 5.0% | 2.6% | 0.52 (0.25–0.86) | 3.8% | 3.6% | 0.94 (0.59–1.36) |
| Cancer |  |  |  |  |  |  |
| + Cardiovascular | 69.7% | 55.6% | 0.80 (0.70–0.91) | 67.3% | 79.3% | 1.18 (1.11–1.24) |
| + Musculoskeletal | 33.5% | 24.8% | 0.74 (0.54–0.93) | 51.7% | 59.8% | 1.16 (1.06–1.27) |
| + Mental disorder | 20.8% | 26.6% | 1.28 (0.98–1.59) | 38.4% | 46.2% | 1.20 (1.07–1.34) |
| + Diabetes | 22.1% | 19.2% | 0.87 (0.60–1.13) | 21.5% | 26.3% | 1.22 (1.02–1.44) |
| + Respiratory | 15.8% | 19.7% | 1.25 (0.88–1.64) | 14.5% | 20.7% | 1.43 (1.14–1.73) |
| + Stomach ulcer | 12.3% | 28.2% | 2.29 (1.77–2.95) | 14.9% | 18.1% | 1.21 (0.96–1.49) |
| + Dementia | 4.2% | 2.2% | 0.53 (0.12–1.13) | 2.6% | 1.3% | 0.51 (0.10–0.98) |
| Stomach ulcer |  |  |  |  |  |  |
| + Cardiovascular | 67.4% | 63.2% | 0.94 (0.87–1.00) | 69.9% | 83.3% | 1.19 (1.15–1.23) |
| + Musculoskeletal | 43.8% | 41.4% | 0.94 (0.84–1.05) | 61.0% | 68.4% | 1.12 (1.06–1.18) |
| + Mental disorder | 28.5% | 22.8% | 0.80 (0.67–0.95) | 45.5% | 49.6% | 1.09 (1.01–1.17) |
| + Diabetes | 17.5% | 12.3% | 0.70 (0.55–0.86) | 19.9% | 28.8% | 1.45 (1.29–1.64) |
| + Respiratory | 16.2% | 17.2% | 1.06 (0.85–1.27) | 19.4% | 25.4% | 1.31 (1.15–1.49) |
| + Cancer | 8.0% | 7.1% | 0.89 (0.61–1.20) | 11.7% | 8.9% | 0.76 (0.59–0.94) |
| + Dementia | 3.6% | 3.6% | 0.99 (0.56–1.52) | 3.4% | 3.9% | 1.15 (0.76–1.66) |
| Dementia |  |  |  |  |  |  |
| + Cardiovascular | 79.6% | 64.8% | 0.81 (0.68–0.94) | 77.4% | 83.7% | 1.08 (0.98–1.17) |
| + Musculoskeletal | 48.0% | 59.0% | 1.23 (0.98–1.46) | 57.3% | 66.4% | 1.16 (1.00–1.32) |
| + Mental disorder | 60.0% | 58.1% | 0.97 (0.77–1.16) | 71.1% | 65.7% | 0.92 (0.80–1.03) |
| + Diabetes | 26.0% | 12.1% | 0.47 (0.21–0.76) | 23.2% | 38.7% | 1.67 (1.30–2.13) |
| + Respiratory | 22.8% | 19.4% | 0.85 (0.49–1.29) | 19.9% | 29.3% | 1.47 (1.06–1.95) |
| + Cancer | 11.5% | 6.8% | 0.59 (0.12–1.11) | 8.1% | 4.1% | 0.51 (0.10–1.09) |
| + Stomach ulcer | 15.3% | 40.1% | 2.62 (1.82–3.42) | 14.3% | 30.6% | 2.14 (1.55–2.88) |

*Notes:* Values are age-standardized using the 2013 European standard population

# Section IV: Supplemental Analysis on Adjusted Risk Ratios

## **Table S8.** Adjusted risk ratios for multimorbidity that includes each chronic disease among men

|  | **Have multimorbidity including this condition** | | | | | | | | | | | | | | | |
| --- | --- | --- | --- | --- | --- | --- | --- | --- | --- | --- | --- | --- | --- | --- | --- | --- |
|  | **Cardiovascular diseases** | | **Musculoskeletal diseases** | | **Mental disorder** | | **Diabetes** | | **Respiratory diseases** | | **Cancer** | | **Stomach ulcer** | | **Dementia** | |
| (Intercept) | -0.909 | *** | -2.201 | *** | -1.771 | *** | -2.735 | *** | -2.195 | *** | -3.827 | *** | -2.667 | *** | -4.658 | *** |
|  | (0.021) |  | (0.050) |  | (0.057) |  | (0.060) |  | (0.076) |  | (0.100) |  | (0.093) |  | (0.161) |  |
| Immigrant | -0.025 |  | 0.085 |  | -0.041 |  | 0.293 | *** | 0.139 |  | -0.174 |  | 0.317 | *** | 0.092 |  |
|  | (0.019) |  | (0.045) |  | (0.068) |  | (0.075) |  | (0.088) |  | (0.105) |  | (0.087) |  | (0.205) |  |
| Have cardiovascular |  |  | 0.256 | *** | 0.300 | *** | 1.013 | *** | 0.296 | *** | 0.126 | *** | 0.179 | *** | 0.244 | *** |
|  |  |  | (0.016) |  | (0.023) |  | (0.027) |  | (0.030) |  | (0.034) |  | (0.035) |  | (0.064) |  |
| Have musculoskeletal | 0.112 | *** |  |  | 0.442 | *** | 0.096 | *** | 0.454 | *** | 0.094 | ** | 0.471 | *** | 0.147 | ** |
|  | (0.007) |  |  |  | (0.021) |  | (0.020) |  | (0.027) |  | (0.032) |  | (0.032) |  | (0.055) |  |
| Have mental disorder | 0.112 | *** | 0.347 | *** |  |  | 0.187 | *** | 0.470 | *** | 0.316 | *** | 0.632 | *** | 1.482 | *** |
|  | (0.008) |  | (0.016) |  |  |  | (0.024) |  | (0.030) |  | (0.037) |  | (0.036) |  | (0.056) |  |
| Have diabetes | 0.304 | *** | 0.074 | *** | 0.182 | *** |  |  | 0.166 | *** | 0.046 |  | -0.028 |  | 0.089 |  |
|  | (0.006) |  | (0.016) |  | (0.024) |  |  |  | (0.030) |  | (0.038) |  | (0.039) |  | (0.057) |  |
| Have respiratory | 0.088 | *** | 0.293 | *** | 0.371 | *** | 0.149 | *** |  |  | 0.255 | *** | 0.477 | *** | 0.258 | *** |
|  | (0.009) |  | (0.017) |  | (0.025) |  | (0.027) |  |  |  | (0.042) |  | (0.039) |  | (0.064) |  |
| Have cancer | 0.032 | ** | 0.050 | * | 0.239 | *** | 0.040 |  | 0.228 | *** |  |  | 0.279 | *** | -0.000 |  |
|  | (0.010) |  | (0.021) |  | (0.029) |  | (0.031) |  | (0.038) |  |  |  | (0.046) |  | (0.075) |  |
| Have stomach ulcer | 0.054 | *** | 0.291 | *** | 0.479 | *** | -0.023 |  | 0.452 | *** | 0.294 | *** |  |  | 0.173 | * |
|  | (0.011) |  | (0.019) |  | (0.027) |  | (0.033) |  | (0.036) |  | (0.047) |  |  |  | (0.077) |  |
| Have dementia | 0.032 | * | 0.053 |  | 0.894 | *** | 0.076 |  | 0.192 | *** | -0.005 |  | 0.104 |  |  |  |
|  | (0.014) |  | (0.029) |  | (0.031) |  | (0.042) |  | (0.053) |  | (0.069) |  | (0.068) |  |  |  |
| Cardiovascular × Immigrant |  |  | -0.030 |  | 0.058 |  | -0.188 | * | 0.105 |  | 0.044 |  | -0.155 |  | -0.052 |  |
|  |  |  | (0.050) |  | (0.073) |  | (0.078) |  | (0.097) |  | (0.117) |  | (0.095) |  | (0.221) |  |
| Musculoskeletal × Immigrant | -0.006 |  |  |  | -0.062 |  | 0.074 |  | -0.186 | * | -0.165 |  | -0.071 |  | -0.296 |  |
|  | (0.023) |  |  |  | (0.067) |  | (0.064) |  | (0.085) |  | (0.108) |  | (0.092) |  | (0.179) |  |
| Mental disorder × Immigrant | 0.022 |  | -0.012 |  |  |  | 0.108 |  | 0.007 |  | -0.068 |  | 0.026 |  | -0.051 |  |
|  | (0.027) |  | (0.050) |  |  |  | (0.074) |  | (0.091) |  | (0.125) |  | (0.100) |  | (0.197) |  |
| Diabetes × Immigrant | -0.010 |  | 0.053 |  | 0.077 |  |  |  | -0.112 |  | 0.019 |  | 0.080 |  | 0.265 |  |
|  | (0.022) |  | (0.050) |  | (0.074) |  |  |  | (0.098) |  | (0.125) |  | (0.109) |  | (0.200) |  |
| Respiratory × Immigrant | 0.029 |  | -0.148 | ** | -0.000 |  | -0.120 |  |  |  | 0.288 | * | -0.185 |  | -0.355 |  |
|  | (0.030) |  | (0.057) |  | (0.077) |  | (0.085) |  |  |  | (0.129) |  | (0.111) |  | (0.217) |  |
| Cancer × Immigrant | 0.020 |  | -0.087 |  | -0.076 |  | -0.033 |  | 0.297 | ** |  |  | 0.141 |  | 0.187 |  |
|  | (0.035) |  | (0.072) |  | (0.099) |  | (0.102) |  | (0.114) |  |  |  | (0.134) |  | (0.240) |  |
| Stomach ulcer × Immigrant | -0.045 |  | -0.005 |  | 0.052 |  | 0.071 |  | -0.148 |  | 0.124 |  |  |  | 0.059 |  |
|  | (0.034) |  | (0.058) |  | (0.079) |  | (0.093) |  | (0.107) |  | (0.146) |  |  |  | (0.222) |  |
| Dementia × Immigrant | -0.008 |  | -0.085 |  | 0.032 |  | 0.119 |  | -0.266 |  | 0.118 |  | 0.106 |  |  |  |
|  | (0.045) |  | (0.107) |  | (0.104) |  | (0.136) |  | (0.182) |  | (0.217) |  | (0.189) |  |  |  |
| Age group (ref: 50–59) |  |  |  |  |  |  |  |  |  |  |  |  |  |  |  |  |
| 60–69 | 0.238 | *** | 0.186 | *** | -0.227 | *** | 0.212 | *** | 0.097 | ** | 0.384 | *** | 0.012 |  | 0.093 |  |
|  | (0.009) |  | (0.017) |  | (0.021) |  | (0.024) |  | (0.031) |  | (0.045) |  | (0.033) |  | (0.083) |  |
| 70–79 | 0.378 | *** | 0.324 | *** | -0.350 | *** | 0.276 | *** | 0.186 | *** | 0.785 | *** | 0.031 |  | 0.779 | *** |
|  | (0.010) |  | (0.020) |  | (0.027) |  | (0.029) |  | (0.038) |  | (0.051) |  | (0.042) |  | (0.089) |  |
| Education (ref: Low) |  |  |  |  |  |  |  |  |  |  |  |  |  |  |  |  |
| Medium | -0.024 | ** | -0.098 | *** | -0.001 |  | -0.065 | ** | -0.266 | *** | 0.041 |  | -0.106 | ** | -0.219 | *** |
|  | (0.008) |  | (0.017) |  | (0.026) |  | (0.024) |  | (0.033) |  | (0.040) |  | (0.038) |  | (0.066) |  |
| High | -0.052 | *** | -0.248 | *** | 0.010 |  | -0.192 | *** | -0.423 | *** | 0.131 | ** | -0.253 | *** | -0.171 | * |
|  | (0.010) |  | (0.021) |  | (0.030) |  | (0.030) |  | (0.041) |  | (0.044) |  | (0.048) |  | (0.078) |  |
| Income (ref: Low) |  |  |  |  |  |  |  |  |  |  |  |  |  |  |  |  |
| Medium | 0.012 | * | -0.010 |  | 0.029 |  | -0.009 |  | -0.063 | ** | 0.056 | * | -0.010 |  | -0.101 | * |
|  | (0.006) |  | (0.012) |  | (0.017) |  | (0.017) |  | (0.022) |  | (0.027) |  | (0.026) |  | (0.048) |  |
| High | 0.001 |  | -0.048 | *** | -0.020 |  | -0.052 | ** | -0.108 | *** | 0.076 | ** | -0.060 | * | -0.105 | * |
|  | (0.006) |  | (0.012) |  | (0.018) |  | (0.017) |  | (0.023) |  | (0.027) |  | (0.027) |  | (0.048) |  |
| Working (ref: Not working) | -0.216 | *** | -0.326 | *** | -0.525 | *** | -0.315 | *** | -0.410 | *** | -0.587 | *** | -0.183 | *** | -1.516 | *** |
|  | (0.010) |  | (0.018) |  | (0.027) |  | (0.026) |  | (0.036) |  | (0.047) |  | (0.037) |  | (0.125) |  |
| Married (ref: Not married) | 0.040 | *** | 0.000 |  | -0.296 | *** | -0.058 | * | -0.101 | *** | 0.151 | *** | -0.080 | * | 0.067 |  |
|  | (0.009) |  | (0.017) |  | (0.022) |  | (0.024) |  | (0.030) |  | (0.039) |  | (0.036) |  | (0.062) |  |
| Country (ref: Austria) |  |  |  |  |  |  |  |  |  |  |  |  |  |  |  |  |
| Belgium | -0.088 | *** | 0.560 | *** | 0.403 | *** | -0.128 | * | -0.165 | * | 0.194 | * | 0.092 |  | -0.828 | *** |
|  | (0.020) |  | (0.046) |  | (0.053) |  | (0.059) |  | (0.072) |  | (0.089) |  | (0.087) |  | (0.142) |  |
| Bulgaria | 0.054 |  | -0.403 | *** | -0.755 | *** | -0.348 | *** | -0.625 | *** | -1.090 | *** | -0.438 | * | -0.904 | ** |
|  | (0.028) |  | (0.096) |  | (0.138) |  | (0.103) |  | (0.171) |  | (0.232) |  | (0.186) |  | (0.296) |  |
| Croatia | -0.065 | ** | -0.105 |  | -0.105 |  | -0.049 |  | -0.712 | *** | -0.109 |  | -0.159 |  | -0.555 | ** |
|  | (0.022) |  | (0.059) |  | (0.067) |  | (0.064) |  | (0.100) |  | (0.107) |  | (0.111) |  | (0.172) |  |
| Cyprus | -0.071 |  | -0.575 | *** | -0.794 | *** | 0.241 | ** | -0.915 | *** | -0.671 | ** | -0.857 | ** | -0.387 |  |
|  | (0.037) |  | (0.131) |  | (0.179) |  | (0.091) |  | (0.230) |  | (0.229) |  | (0.282) |  | (0.295) |  |
| Czech Republic | 0.048 | ** | 0.468 | *** | -0.323 | *** | 0.221 | *** | -0.206 | ** | 0.016 |  | 0.046 |  | -0.387 | ** |
|  | (0.018) |  | (0.046) |  | (0.062) |  | (0.053) |  | (0.075) |  | (0.094) |  | (0.091) |  | (0.137) |  |
| Denmark | -0.130 | *** | 0.671 | *** | -0.081 |  | -0.374 | *** | 0.112 |  | 0.217 | * | -0.240 | * | -0.574 | ** |
|  | (0.024) |  | (0.050) |  | (0.069) |  | (0.075) |  | (0.080) |  | (0.099) |  | (0.109) |  | (0.182) |  |
| Estonia | 0.060 | ** | 0.418 | *** | -0.059 |  | -0.294 | *** | -0.163 | * | 0.199 | * | 0.507 | *** | -0.246 |  |
|  | (0.019) |  | (0.048) |  | (0.058) |  | (0.063) |  | (0.076) |  | (0.092) |  | (0.084) |  | (0.132) |  |
| Finland | -0.082 | ** | 0.202 | ** | -0.429 | *** | 0.001 |  | -0.409 | *** | 0.254 | * | -0.736 | *** | -0.483 | * |
|  | (0.028) |  | (0.066) |  | (0.099) |  | (0.079) |  | (0.117) |  | (0.118) |  | (0.178) |  | (0.221) |  |
| France | -0.186 | *** | 0.624 | *** | 0.239 | *** | -0.043 |  | -0.187 | * | 0.102 |  | -0.369 | *** | -0.697 | *** |
|  | (0.022) |  | (0.047) |  | (0.058) |  | (0.061) |  | (0.078) |  | (0.095) |  | (0.103) |  | (0.159) |  |
| Germany | 0.019 |  | 0.515 | *** | -0.256 | *** | 0.017 |  | -0.035 |  | 0.428 | *** | -0.217 | * | -0.319 | * |
|  | (0.019) |  | (0.047) |  | (0.061) |  | (0.057) |  | (0.074) |  | (0.085) |  | (0.096) |  | (0.133) |  |
| Greece | -0.059 | ** | -0.482 | *** | -0.607 | *** | -0.007 |  | -0.511 | *** | -0.796 | *** | 0.232 | * | -0.790 | *** |
|  | (0.021) |  | (0.066) |  | (0.083) |  | (0.063) |  | (0.095) |  | (0.129) |  | (0.099) |  | (0.180) |  |
| Hungary | 0.039 |  | 0.666 | *** | -0.458 | *** | 0.249 | *** | -0.442 | *** | -0.267 | * | -0.096 |  | -0.125 |  |
|  | (0.022) |  | (0.051) |  | (0.081) |  | (0.061) |  | (0.103) |  | (0.127) |  | (0.115) |  | (0.164) |  |
| Ireland | -0.259 | *** | 0.313 | ** | -0.023 |  | -0.096 |  | -0.104 |  | 0.079 |  | -0.187 |  | -0.455 |  |
|  | (0.061) |  | (0.111) |  | (0.128) |  | (0.154) |  | (0.155) |  | (0.241) |  | (0.196) |  | (0.501) |  |
| Italy | -0.020 |  | 0.325 | *** | -0.111 |  | -0.064 |  | -0.254 | ** | -0.227 | * | -0.164 |  | -0.722 | *** |
|  | (0.020) |  | (0.050) |  | (0.063) |  | (0.059) |  | (0.077) |  | (0.101) |  | (0.099) |  | (0.147) |  |
| Latvia | -0.060 |  | -0.045 |  | -0.726 | *** | -0.553 | *** | -1.028 | *** | -0.653 | *** | -0.095 |  | -1.195 | ** |
|  | (0.031) |  | (0.083) |  | (0.125) |  | (0.115) |  | (0.182) |  | (0.187) |  | (0.155) |  | (0.394) |  |
| Lithuania | -0.006 |  | -0.292 | ** | -0.080 |  | -0.400 | *** | -0.151 |  | 0.123 |  | 0.428 | *** | -0.285 |  |
|  | (0.031) |  | (0.091) |  | (0.090) |  | (0.110) |  | (0.125) |  | (0.142) |  | (0.123) |  | (0.214) |  |
| Luxembourg | -0.114 | *** | 0.561 | *** | -0.145 |  | -0.103 |  | -0.263 | * | 0.478 | *** | -0.004 |  | -0.324 |  |
|  | (0.030) |  | (0.058) |  | (0.083) |  | (0.085) |  | (0.109) |  | (0.117) |  | (0.125) |  | (0.199) |  |
| Malta | -0.110 | ** | -0.247 | * | -0.494 | *** | 0.240 | ** | -1.287 | *** | -0.327 |  | -0.460 | * | -0.934 | * |
|  | (0.036) |  | (0.100) |  | (0.137) |  | (0.088) |  | (0.217) |  | (0.190) |  | (0.212) |  | (0.379) |  |
| Netherlands | -0.196 | *** | -0.081 |  | 0.184 | ** | -0.126 |  | 0.055 |  | 0.283 | ** | -0.626 | *** | -0.649 | *** |
|  | (0.026) |  | (0.062) |  | (0.067) |  | (0.069) |  | (0.083) |  | (0.100) |  | (0.131) |  | (0.175) |  |
| Poland | -0.044 | * | 0.550 | *** | -0.411 | *** | 0.003 |  | -0.250 | ** | -0.196 |  | 0.050 |  | -0.344 | * |
|  | (0.020) |  | (0.047) |  | (0.066) |  | (0.058) |  | (0.080) |  | (0.100) |  | (0.094) |  | (0.142) |  |
| Portugal | -0.057 | * | 0.073 |  | 0.400 | *** | 0.179 | * | -0.448 | *** | 0.056 |  | 0.139 |  | -0.754 | *** |
|  | (0.026) |  | (0.067) |  | (0.068) |  | (0.072) |  | (0.108) |  | (0.125) |  | (0.116) |  | (0.193) |  |
| Romania | -0.178 | *** | -0.158 | * | -0.627 | *** | -0.310 | ** | -0.524 | *** | -0.823 | *** | -0.521 | ** | -0.935 | ** |
|  | (0.033) |  | (0.078) |  | (0.122) |  | (0.098) |  | (0.139) |  | (0.191) |  | (0.170) |  | (0.290) |  |
| Slovakia | -0.359 | *** | -0.385 | *** | -0.701 | *** | -0.410 | *** | -1.067 | *** | -1.212 | *** | -0.386 | * | -1.258 | *** |
|  | (0.039) |  | (0.095) |  | (0.130) |  | (0.105) |  | (0.183) |  | (0.228) |  | (0.183) |  | (0.357) |  |
| Slovenia | -0.014 |  | -0.306 | *** | -0.315 | *** | 0.025 |  | -0.324 | *** | 0.020 |  | 0.149 |  | -0.180 |  |
|  | (0.020) |  | (0.060) |  | (0.068) |  | (0.060) |  | (0.086) |  | (0.099) |  | (0.097) |  | (0.135) |  |
| Spain | -0.118 | *** | 0.181 | *** | 0.135 | * | 0.186 | *** | -0.228 | ** | -0.067 |  | -0.161 |  | -0.443 | *** |
|  | (0.020) |  | (0.051) |  | (0.058) |  | (0.056) |  | (0.077) |  | (0.098) |  | (0.098) |  | (0.134) |  |
| Sweden | -0.096 | *** | 0.160 | ** | -0.071 |  | -0.115 |  | -0.276 | ** | 0.338 | *** | -0.467 | *** | -0.468 | ** |
|  | (0.022) |  | (0.054) |  | (0.069) |  | (0.066) |  | (0.089) |  | (0.093) |  | (0.117) |  | (0.151) |  |
| Switzerland | -0.131 | *** | 0.350 | *** | 0.221 | ** | -0.281 | *** | -0.279 | ** | 0.327 | ** | -0.782 | *** | -0.653 | *** |
|  | (0.026) |  | (0.055) |  | (0.067) |  | (0.077) |  | (0.096) |  | (0.104) |  | (0.144) |  | (0.197) |  |
| Wave (ref: Wave 2) |  |  |  |  |  |  |  |  |  |  |  |  |  |  |  |  |
| Wave 4 | 0.051 | *** | 0.040 | * | -0.010 |  | 0.106 | *** | -0.105 | *** | 0.144 | *** | -0.101 | *** | 0.254 | ** |
|  | (0.009) |  | (0.016) |  | (0.020) |  | (0.023) |  | (0.025) |  | (0.040) |  | (0.030) |  | (0.088) |  |
| Wave 5 | 0.081 | *** | 0.165 | *** | -0.051 | * | 0.178 | *** | -0.174 | *** | 0.300 | *** | -0.126 | *** | 0.427 | *** |
|  | (0.009) |  | (0.018) |  | (0.021) |  | (0.024) |  | (0.026) |  | (0.041) |  | (0.032) |  | (0.090) |  |
| Wave 6 | 0.105 | *** | 0.293 | *** | 0.003 |  | 0.201 | *** | -0.110 | *** | 0.358 | *** | -0.096 | ** | 0.607 | *** |
|  | (0.009) |  | (0.018) |  | (0.022) |  | (0.024) |  | (0.027) |  | (0.043) |  | (0.032) |  | (0.090) |  |
| Wave 7 | 0.135 | *** | 0.363 | *** | 0.059 | * | 0.223 | *** | -0.164 | *** | 0.459 | *** | -0.056 |  | 0.630 | *** |
|  | (0.010) |  | (0.019) |  | (0.024) |  | (0.026) |  | (0.030) |  | (0.044) |  | (0.035) |  | (0.094) |  |
| Wave 8 | 0.142 | *** | 0.423 | *** | 0.071 | ** | 0.250 | *** | -0.139 | *** | 0.475 | *** | -0.063 |  | 0.651 | *** |
|  | (0.010) |  | (0.019) |  | (0.025) |  | (0.027) |  | (0.032) |  | (0.046) |  | (0.038) |  | (0.096) |  |
| Wave 9 | 0.158 | *** | 0.404 | *** | 0.060 | * | 0.286 | *** | -0.112 | *** | 0.493 | *** | -0.131 | *** | 0.656 | *** |
|  | (0.010) |  | (0.019) |  | (0.025) |  | (0.027) |  | (0.032) |  | (0.046) |  | (0.038) |  | (0.095) |  |
| n | 58,558 |  | 58,558 |  | 58,558 |  | 58,558 |  | 58,558 |  | 58,558 |  | 58,558 |  | 58,558 |  |
| N | 153,392 |  | 153,392 |  | 153,392 |  | 153,392 |  | 153,392 |  | 153,392 |  | 153,392 |  | 153,392 |  |
| AIC | 267,052 |  | 181,251 |  | 130,123 |  | 135,469 |  | 97,581 |  | 77,664 |  | 83,276 |  | 29,342 |  |

* p < 0.05; ** p < 0.01; *** p < 0.001

## **Table S9.** Adjusted risk ratios for multimorbidity including each chronic disease among women

|  | **Have multimorbidity including this condition** | | | | | | | | | | | | | | | |
| --- | --- | --- | --- | --- | --- | --- | --- | --- | --- | --- | --- | --- | --- | --- | --- | --- |
|  | **Cardiovascular diseases** | | **Musculoskeletal diseases** | | **Mental disorder** | | **Diabetes** | | **Respiratory diseases** | | **Cancer** | | **Stomach ulcer** | | **Dementia** | |
| (Intercept) | -1.003 | *** | -1.529 | *** | -1.418 | *** | -3.085 | *** | -2.761 | *** | -3.283 | *** | -3.289 | *** | -5.037 | *** |
|  | (0.019) |  | (0.026) |  | (0.034) |  | (0.061) |  | (0.067) |  | (0.075) |  | (0.078) |  | (0.149) |  |
| Immigrant | 0.067 | *** | 0.057 | * | -0.021 |  | 0.227 | ** | 0.180 | * | -0.145 |  | 0.263 | ** | 0.142 |  |
|  | (0.019) |  | (0.028) |  | (0.044) |  | (0.085) |  | (0.084) |  | (0.087) |  | (0.091) |  | (0.214) |  |
| Have cardiovascular |  |  | 0.191 | *** | 0.227 | *** | 1.206 | *** | 0.367 | *** | 0.083 | ** | 0.187 | *** | 0.279 | *** |
|  |  |  | (0.009) |  | (0.014) |  | (0.029) |  | (0.028) |  | (0.029) |  | (0.033) |  | (0.062) |  |
| Have musculoskeletal | 0.144 | *** |  |  | 0.458 | *** | 0.079 | *** | 0.574 | *** | 0.153 | *** | 0.681 | *** | 0.069 |  |
|  | (0.007) |  |  |  | (0.013) |  | (0.020) |  | (0.027) |  | (0.028) |  | (0.032) |  | (0.054) |  |
| Have mental disorder | 0.118 | *** | 0.302 | *** |  |  | 0.186 | *** | 0.472 | *** | 0.292 | *** | 0.684 | *** | 1.299 | *** |
|  | (0.007) |  | (0.009) |  |  |  | (0.021) |  | (0.026) |  | (0.030) |  | (0.031) |  | (0.057) |  |
| Have diabetes | 0.326 | *** | 0.035 | *** | 0.133 | *** |  |  | 0.267 | *** | 0.148 | *** | 0.095 | ** | 0.283 | *** |
|  | (0.006) |  | (0.010) |  | (0.015) |  |  |  | (0.030) |  | (0.035) |  | (0.035) |  | (0.057) |  |
| Have respiratory | 0.112 | *** | 0.224 | *** | 0.274 | *** | 0.238 | *** |  |  | 0.173 | *** | 0.422 | *** | 0.279 | *** |
|  | (0.009) |  | (0.010) |  | (0.015) |  | (0.027) |  |  |  | (0.038) |  | (0.036) |  | (0.063) |  |
| Have cancer | 0.025 | * | 0.066 | *** | 0.178 | *** | 0.121 | *** | 0.169 | *** |  |  | 0.240 | *** | 0.005 |  |
|  | (0.010) |  | (0.012) |  | (0.018) |  | (0.030) |  | (0.036) |  |  |  | (0.042) |  | (0.076) |  |
| Have stomach ulcer | 0.051 | *** | 0.249 | *** | 0.379 | *** | 0.081 | ** | 0.397 | *** | 0.247 | *** |  |  | 0.081 |  |
|  | (0.010) |  | (0.011) |  | (0.016) |  | (0.031) |  | (0.034) |  | (0.043) |  |  |  | (0.071) |  |
| Have dementia | 0.031 | * | 0.006 |  | 0.562 | *** | 0.196 | *** | 0.240 | *** | 0.015 |  | 0.053 |  |  |  |
|  | (0.013) |  | (0.018) |  | (0.021) |  | (0.041) |  | (0.051) |  | (0.070) |  | (0.062) |  |  |  |
| Cardiovascular x Immigrant |  |  | 0.024 |  | 0.004 |  | -0.015 |  | -0.018 |  | 0.077 |  | 0.076 |  | -0.272 |  |
|  |  |  | (0.030) |  | (0.045) |  | (0.088) |  | (0.089) |  | (0.100) |  | (0.090) |  | (0.195) |  |
| Musculoskeletal x Immigrant | -0.013 |  |  |  | -0.004 |  | 0.024 |  | -0.072 |  | -0.030 |  | 0.041 |  | 0.024 |  |
|  | (0.021) |  |  |  | (0.042) |  | (0.058) |  | (0.080) |  | (0.089) |  | (0.088) |  | (0.168) |  |
| Mental disorder x Immigrant | -0.010 |  | -0.018 |  |  |  | 0.069 |  | 0.002 |  | 0.009 |  | -0.061 |  | 0.037 |  |
|  | (0.022) |  | (0.026) |  |  |  | (0.060) |  | (0.078) |  | (0.093) |  | (0.082) |  | (0.177) |  |
| Diabetes x Immigrant | -0.052 | ** | 0.007 |  | 0.033 |  |  |  | -0.144 |  | -0.108 |  | -0.041 |  | -0.119 |  |
|  | (0.019) |  | (0.028) |  | (0.043) |  |  |  | (0.084) |  | (0.111) |  | (0.087) |  | (0.178) |  |
| Respiratory x Immigrant | -0.005 |  | -0.021 |  | 0.025 |  | -0.097 |  |  |  | 0.228 | * | -0.005 |  | 0.080 |  |
|  | (0.027) |  | (0.029) |  | (0.046) |  | (0.075) |  |  |  | (0.113) |  | (0.091) |  | (0.204) |  |
| Cancer x Immigrant | 0.020 |  | -0.015 |  | -0.007 |  | -0.075 |  | 0.167 |  |  |  | -0.024 |  | 0.080 |  |
|  | (0.031) |  | (0.036) |  | (0.055) |  | (0.092) |  | (0.102) |  |  |  | (0.111) |  | (0.261) |  |
| Stomach ulcer x Immigrant | 0.006 |  | 0.020 |  | -0.019 |  | 0.016 |  | 0.008 |  | -0.014 |  |  |  | -0.135 |  |
|  | (0.026) |  | (0.029) |  | (0.046) |  | (0.077) |  | (0.092) |  | (0.124) |  |  |  | (0.206) |  |
| Dementia x Immigrant | -0.060 |  | 0.005 |  | 0.018 |  | -0.036 |  | -0.048 |  | 0.023 |  | -0.071 |  |  |  |
|  | (0.046) |  | (0.053) |  | (0.062) |  | (0.117) |  | (0.160) |  | (0.232) |  | (0.168) |  |  |  |
| Age group (ref:50-59) |  |  |  |  |  |  |  |  |  |  |  |  |  |  |  |  |
| 60-69 | 0.281 | *** | 0.265 | *** | -0.111 | *** | 0.187 | *** | -0.039 |  | 0.150 | *** | 0.015 |  | 0.294 | *** |
|  | (0.009) |  | (0.010) |  | (0.013) |  | (0.024) |  | (0.026) |  | (0.030) |  | (0.030) |  | (0.082) |  |
| 70-79 | 0.463 | *** | 0.411 | *** | -0.196 | *** | 0.284 | *** | -0.052 |  | 0.291 | *** | -0.024 |  | 1.006 | *** |
|  | (0.010) |  | (0.012) |  | (0.016) |  | (0.029) |  | (0.033) |  | (0.037) |  | (0.037) |  | (0.092) |  |
| Education (ref: Low) |  |  |  |  |  |  |  |  |  |  |  |  |  |  |  |  |
| Medium | -0.076 | *** | -0.042 | *** | -0.068 | *** | -0.221 | *** | -0.141 | *** | 0.155 | *** | -0.049 |  | -0.378 | *** |
|  | (0.008) |  | (0.010) |  | (0.015) |  | (0.023) |  | (0.029) |  | (0.034) |  | (0.033) |  | (0.061) |  |
| High | -0.186 | *** | -0.098 | *** | -0.105 | *** | -0.382 | *** | -0.181 | *** | 0.230 | *** | -0.214 | *** | -0.317 | *** |
|  | (0.011) |  | (0.013) |  | (0.019) |  | (0.034) |  | (0.038) |  | (0.040) |  | (0.044) |  | (0.083) |  |
| Income (ref: Low) |  |  |  |  |  |  |  |  |  |  |  |  |  |  |  |  |
| Medium | -0.008 |  | 0.024 | *** | 0.019 |  | -0.087 | *** | -0.057 | ** | 0.036 |  | 0.031 |  | -0.095 | * |
|  | (0.005) |  | (0.007) |  | (0.010) |  | (0.016) |  | (0.020) |  | (0.022) |  | (0.023) |  | (0.043) |  |
| High | -0.024 | *** | 0.002 |  | 0.024 | * | -0.152 | *** | -0.050 | * | 0.065 | ** | -0.015 |  | -0.189 | *** |
|  | (0.006) |  | (0.007) |  | (0.010) |  | (0.017) |  | (0.021) |  | (0.023) |  | (0.024) |  | (0.047) |  |
| Working (ref: Not working) | -0.219 | *** | -0.243 | *** | -0.288 | *** | -0.395 | *** | -0.279 | *** | -0.448 | *** | -0.168 | *** | -1.144 | *** |
|  | (0.011) |  | (0.012) |  | (0.016) |  | (0.032) |  | (0.033) |  | (0.036) |  | (0.037) |  | (0.133) |  |
| Married (ref: Not married) | -0.005 |  | -0.013 |  | -0.208 | *** | -0.057 | ** | -0.174 | *** | 0.011 |  | -0.069 | * | -0.016 |  |
|  | (0.007) |  | (0.008) |  | (0.012) |  | (0.019) |  | (0.024) |  | (0.028) |  | (0.028) |  | (0.049) |  |
| Country (ref: Austria) |  |  |  |  |  |  |  |  |  |  |  |  |  |  |  |  |
| Belgium | -0.041 | * | 0.395 | *** | 0.364 | *** | 0.003 |  | -0.035 |  | 0.099 |  | 0.141 | * | -0.415 | ** |
|  | (0.020) |  | (0.024) |  | (0.032) |  | (0.061) |  | (0.068) |  | (0.076) |  | (0.071) |  | (0.139) |  |
| Bulgaria | 0.252 | *** | -0.308 | *** | -0.787 | *** | 0.095 |  | -0.163 |  | -0.789 | *** | -0.543 | ** | -0.196 |  |
|  | (0.024) |  | (0.053) |  | (0.085) |  | (0.086) |  | (0.130) |  | (0.177) |  | (0.170) |  | (0.248) |  |
| Croatia | 0.093 | *** | -0.012 |  | -0.220 | *** | -0.014 |  | -0.508 | *** | 0.098 |  | -0.285 | ** | -0.302 |  |
|  | (0.021) |  | (0.031) |  | (0.043) |  | (0.067) |  | (0.094) |  | (0.086) |  | (0.096) |  | (0.163) |  |
| Cyprus | -0.042 |  | 0.074 |  | -0.682 | *** | 0.208 | * | -0.424 | * | -0.370 | * | -0.236 |  | -0.028 |  |
|  | (0.036) |  | (0.049) |  | (0.101) |  | (0.102) |  | (0.170) |  | (0.181) |  | (0.170) |  | (0.253) |  |
| Czech Republic | 0.092 | *** | 0.178 | *** | -0.085 | * | 0.310 | *** | 0.161 | * | -0.001 |  | -0.185 | * | -0.322 | * |
|  | (0.018) |  | (0.025) |  | (0.035) |  | (0.054) |  | (0.066) |  | (0.077) |  | (0.078) |  | (0.136) |  |
| Denmark | -0.078 | ** | 0.341 | *** | -0.167 | *** | -0.239 | ** | 0.443 | *** | 0.334 | *** | -0.343 | *** | -0.460 | * |
|  | (0.026) |  | (0.029) |  | (0.046) |  | (0.083) |  | (0.074) |  | (0.084) |  | (0.100) |  | (0.205) |  |
| Estonia | 0.176 | *** | 0.119 | *** | -0.016 |  | 0.167 | ** | 0.064 |  | 0.018 |  | 0.297 | *** | 0.101 |  |
|  | (0.018) |  | (0.027) |  | (0.035) |  | (0.057) |  | (0.069) |  | (0.078) |  | (0.070) |  | (0.129) |  |
| Finland | -0.002 |  | 0.126 | ** | -0.617 | *** | 0.131 |  | 0.103 |  | 0.258 | * | -0.913 | *** | -0.450 |  |
|  | (0.031) |  | (0.040) |  | (0.076) |  | (0.088) |  | (0.107) |  | (0.101) |  | (0.184) |  | (0.254) |  |
| France | -0.168 | *** | 0.422 | *** | 0.387 | *** | -0.120 |  | -0.032 |  | 0.124 |  | -0.578 | *** | -0.676 | *** |
|  | (0.023) |  | (0.025) |  | (0.033) |  | (0.067) |  | (0.073) |  | (0.080) |  | (0.089) |  | (0.159) |  |
| Germany | 0.055 | ** | 0.181 | *** | -0.120 | ** | 0.070 |  | 0.270 | *** | 0.400 | *** | -0.539 | *** | 0.043 |  |
|  | (0.020) |  | (0.027) |  | (0.039) |  | (0.062) |  | (0.070) |  | (0.074) |  | (0.095) |  | (0.145) |  |
| Greece | -0.057 | ** | 0.096 | *** | -0.439 | *** | 0.136 | * | -0.295 | *** | -0.815 | *** | 0.156 |  | -0.403 | ** |
|  | (0.021) |  | (0.029) |  | (0.046) |  | (0.062) |  | (0.086) |  | (0.115) |  | (0.083) |  | (0.151) |  |
| Hungary | 0.212 | *** | 0.272 | *** | -0.246 | *** | 0.379 | *** | -0.130 |  | -0.268 | * | -0.053 |  | 0.186 |  |
|  | (0.019) |  | (0.029) |  | (0.046) |  | (0.062) |  | (0.088) |  | (0.106) |  | (0.094) |  | (0.162) |  |
| Ireland | -0.220 | *** | 0.060 |  | -0.430 | *** | -0.131 |  | 0.365 | ** | -0.129 |  | 0.097 |  | -0.345 |  |
|  | (0.060) |  | (0.071) |  | (0.107) |  | (0.172) |  | (0.139) |  | (0.213) |  | (0.185) |  | (0.502) |  |
| Italy | -0.025 |  | 0.337 | *** | -0.119 | ** | -0.029 |  | -0.104 |  | 0.015 |  | -0.219 | ** | -0.523 | *** |
|  | (0.020) |  | (0.025) |  | (0.038) |  | (0.062) |  | (0.075) |  | (0.083) |  | (0.085) |  | (0.143) |  |
| Latvia | 0.263 | *** | -0.241 | *** | -0.673 | *** | 0.020 |  | -0.291 | * | -0.664 | *** | -0.063 |  | -0.130 |  |
|  | (0.023) |  | (0.046) |  | (0.071) |  | (0.084) |  | (0.124) |  | (0.142) |  | (0.118) |  | (0.237) |  |
| Lithuania | 0.326 | *** | -0.324 | *** | 0.012 |  | -0.248 | * | -0.003 |  | -0.158 |  | 0.453 | *** | 0.327 |  |
|  | (0.023) |  | (0.050) |  | (0.051) |  | (0.098) |  | (0.103) |  | (0.119) |  | (0.096) |  | (0.169) |  |
| Luxembourg | -0.119 | *** | 0.329 | *** | -0.049 |  | -0.251 | * | -0.039 |  | 0.422 | *** | -0.009 |  | -0.173 |  |
|  | (0.034) |  | (0.034) |  | (0.054) |  | (0.101) |  | (0.104) |  | (0.105) |  | (0.107) |  | (0.209) |  |
| Malta | -0.083 | * | -0.028 |  | -0.494 | *** | 0.317 | *** | -0.764 | *** | -0.469 | ** | -0.759 | *** | -0.938 | * |
|  | (0.038) |  | (0.053) |  | (0.091) |  | (0.094) |  | (0.187) |  | (0.178) |  | (0.210) |  | (0.385) |  |
| Netherlands | -0.173 | *** | -0.003 |  | -0.003 |  | -0.132 |  | 0.488 | *** | 0.305 | *** | -0.600 | *** | -0.460 | ** |
|  | (0.026) |  | (0.034) |  | (0.045) |  | (0.076) |  | (0.074) |  | (0.086) |  | (0.117) |  | (0.177) |  |
| Poland | 0.134 | *** | 0.258 | *** | -0.271 | *** | 0.244 | *** | -0.191 | * | -0.208 | * | -0.007 |  | -0.103 |  |
|  | (0.019) |  | (0.026) |  | (0.040) |  | (0.058) |  | (0.079) |  | (0.085) |  | (0.083) |  | (0.141) |  |
| Portugal | 0.046 |  | 0.114 | *** | 0.560 | *** | 0.205 | ** | -0.343 | *** | 0.075 |  | 0.028 |  | -0.252 |  |
|  | (0.024) |  | (0.034) |  | (0.036) |  | (0.072) |  | (0.100) |  | (0.110) |  | (0.096) |  | (0.171) |  |
| Romania | 0.193 | *** | -0.086 | * | -0.783 | *** | 0.040 |  | -0.597 | *** | -1.159 | *** | -0.293 | * | -0.063 |  |
|  | (0.024) |  | (0.041) |  | (0.077) |  | (0.083) |  | (0.132) |  | (0.187) |  | (0.136) |  | (0.210) |  |
| Slovakia | -0.137 | *** | -0.291 | *** | -0.714 | *** | 0.229 | * | -0.554 | *** | -0.897 | *** | -0.411 | * | -0.553 |  |
|  | (0.035) |  | (0.055) |  | (0.085) |  | (0.090) |  | (0.158) |  | (0.178) |  | (0.160) |  | (0.304) |  |
| Slovenia | 0.072 | *** | -0.229 | *** | -0.250 | *** | -0.046 |  | -0.233 | ** | 0.018 |  | 0.203 | * | 0.223 |  |
|  | (0.020) |  | (0.033) |  | (0.043) |  | (0.064) |  | (0.083) |  | (0.085) |  | (0.081) |  | (0.136) |  |
| Spain | -0.107 | *** | 0.267 | *** | 0.289 | *** | 0.239 | *** | -0.320 | *** | -0.189 | * | -0.353 | *** | -0.097 |  |
|  | (0.020) |  | (0.026) |  | (0.034) |  | (0.058) |  | (0.078) |  | (0.089) |  | (0.087) |  | (0.132) |  |
| Sweden | -0.077 | ** | 0.094 | ** | 0.037 |  | -0.189 | * | 0.230 | ** | 0.330 | *** | -0.479 | *** | -0.399 | * |
|  | (0.024) |  | (0.030) |  | (0.042) |  | (0.075) |  | (0.077) |  | (0.081) |  | (0.103) |  | (0.168) |  |
| Switzerland | -0.320 | *** | 0.238 | *** | 0.165 | *** | -0.501 | *** | 0.084 |  | 0.077 |  | -0.978 | *** | -0.563 | ** |
|  | (0.031) |  | (0.031) |  | (0.044) |  | (0.097) |  | (0.090) |  | (0.096) |  | (0.138) |  | (0.209) |  |
| Wave (ref: Wave 2) |  |  |  |  |  |  |  |  |  |  |  |  |  |  |  |  |
| Wave 4 | 0.034 | *** | -0.039 | *** | 0.028 | * | 0.069 | ** | -0.039 |  | 0.099 | *** | 0.030 |  | -0.076 |  |
|  | (0.008) |  | (0.009) |  | (0.012) |  | (0.023) |  | (0.022) |  | (0.030) |  | (0.030) |  | (0.077) |  |
| Wave 5 | 0.053 | *** | 0.040 | *** | -0.039 | ** | 0.120 | *** | -0.123 | *** | 0.251 | *** | 0.077 | * | 0.156 | * |
|  | (0.009) |  | (0.010) |  | (0.013) |  | (0.024) |  | (0.024) |  | (0.031) |  | (0.032) |  | (0.079) |  |
| Wave 6 | 0.061 | *** | 0.107 | *** | 0.015 |  | 0.154 | *** | -0.065 | ** | 0.287 | *** | 0.139 | *** | 0.325 | *** |
|  | (0.009) |  | (0.010) |  | (0.013) |  | (0.024) |  | (0.025) |  | (0.033) |  | (0.032) |  | (0.078) |  |
| Wave 7 | 0.070 | *** | 0.168 | *** | 0.071 | *** | 0.118 | *** | -0.086 | ** | 0.355 | *** | 0.175 | *** | 0.277 | *** |
|  | (0.009) |  | (0.011) |  | (0.014) |  | (0.026) |  | (0.027) |  | (0.035) |  | (0.034) |  | (0.082) |  |
| Wave 8 | 0.068 | *** | 0.192 | *** | 0.076 | *** | 0.194 | *** | -0.006 |  | 0.397 | *** | 0.171 | *** | 0.371 | *** |
|  | (0.010) |  | (0.011) |  | (0.015) |  | (0.027) |  | (0.029) |  | (0.036) |  | (0.037) |  | (0.084) |  |
| Wave 9 | 0.074 | *** | 0.171 | *** | 0.064 | *** | 0.246 | *** | -0.006 |  | 0.418 | *** | 0.159 | *** | 0.398 | *** |
|  | (0.009) |  | (0.011) |  | (0.015) |  | (0.027) |  | (0.028) |  | (0.036) |  | (0.036) |  | (0.082) |  |
| n | 70,873 |  | 70,873 |  | 70,873 |  | 70,873 |  | 70,873 |  | 70,873 |  | 70,873 |  | 70,873 |  |
| N | 193,441 |  | 193,441 |  | 193,441 |  | 193,441 |  | 193,441 |  | 193,441 |  | 193,441 |  | 193,441 |  |
| AIC | 325,427 |  | 297,984 |  | 237,478 |  | 144,781 |  | 122,345 |  | 111,686 |  | 100,664 |  | 34,199 |  |

* p < 0.05; ** p < 0.01; *** p < 0.001


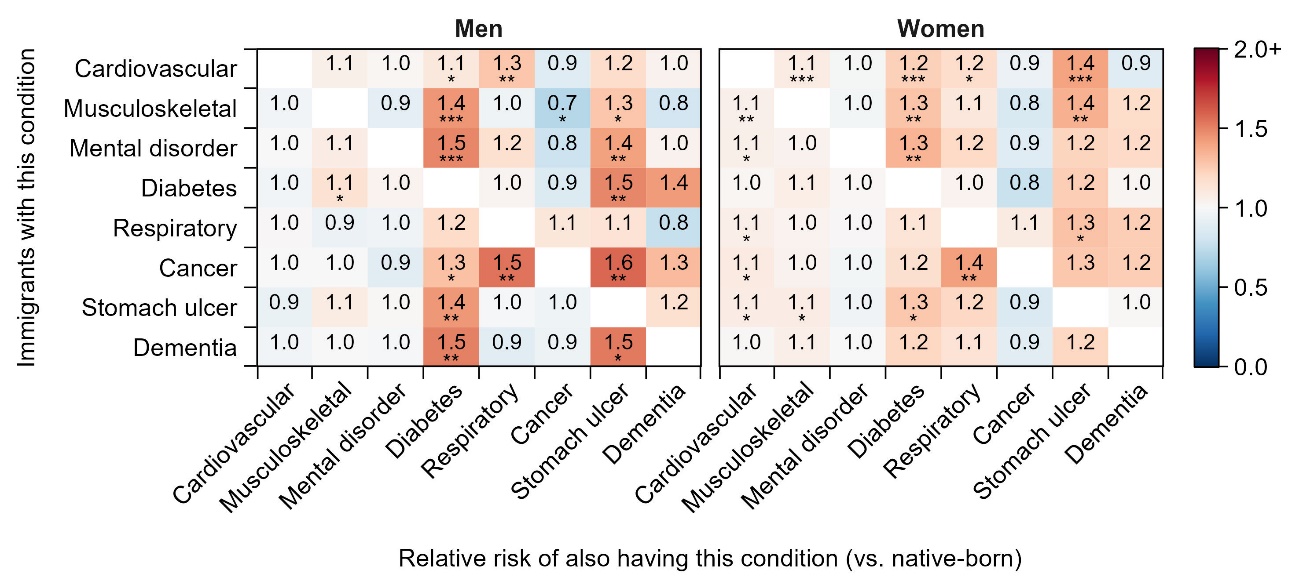


## **Fig. S1** Relative risks of multimorbidity among immigrants versus native-born individuals by gender based on Poisson regression models

* p < 0.05; ** p < 0.01; *** p < 0.001

# Section V: Supplemental Analysis Excluding Pre-Migration Conditions

## **Table S10.** Number and percentage of pre- and post-migration onsets among immigrants with eight chronic diseases by gender, region of origin, and region of residence

|  | **Immigrant men** | | **Immigrant women** | |
| --- | --- | --- | --- | --- |
|  | **Pre-migration** | **Post-migration** | **Pre-migration** | **Post-migration** |
| **Total population** |  |  |  |  |
| Cardiovascular | 326 ( 4.5%) | 6,922 ( 95.5%) | 485 ( 5.0%) | 9,150 ( 95.0%) |
| Musculoskeletal | 171 ( 4.7%) | 3,477 ( 95.3%) | 304 ( 3.8%) | 7,622 ( 96.2%) |
| Mental disorder | 139 ( 6.6%) | 1,981 ( 93.4%) | 337 ( 6.5%) | 4,830 ( 93.5%) |
| Diabetes | 63 ( 2.7%) | 2,306 ( 97.3%) | 84 ( 3.0%) | 2,707 ( 97.0%) |
| Respiratory | 115 ( 8.1%) | 1,311 ( 91.9%) | 201 ( 9.7%) | 1,870 ( 90.3%) |
| Stomach ulcer | 139 (10.5%) | 1,180 ( 89.5%) | 91 ( 4.8%) | 1,818 ( 95.2%) |
| Cancer | 23 ( 2.5%) | 893 ( 97.5%) | 22 ( 1.5%) | 1,446 ( 98.5%) |
| Dementia & Parkinson's | 8 ( 2.6%) | 300 ( 97.4%) | 3 ( 0.8%) | 354 ( 99.2%) |
| **Region of origin: Africa** |  |  |  |  |
| Cardiovascular | 10 ( 1.7%) | 581 ( 98.3%) | 42 ( 7.3%) | 532 ( 92.7%) |
| Musculoskeletal | 7 ( 2.2%) | 312 ( 97.8%) | 21 ( 3.5%) | 577 ( 96.5%) |
| Mental disorder | 6 ( 3.1%) | 187 ( 96.9%) | 39 ( 8.7%) | 409 ( 91.3%) |
| Diabetes | 10 ( 3.6%) | 264 ( 96.4%) | 28 (12.1%) | 204 ( 87.9%) |
| Respiratory | 10 ( 6.7%) | 140 ( 93.3%) | 15 (12.6%) | 104 ( 87.4%) |
| Stomach ulcer | 6 ( 6.8%) | 82 ( 93.2%) | 6 ( 5.2%) | 109 ( 94.8%) |
| Cancer | 0 ( 0.0%) | 66 (100.0%) | 1 ( 1.0%) | 101 ( 99.0%) |
| Dementia & Parkinson's | 0 ( 0.0%) | 11 (100.0%) | 0 ( 0.0%) | 21 (100.0%) |
| **Region of origin: Asia** |  |  |  |  |
| Cardiovascular | 15 ( 4.0%) | 356 ( 96.0%) | 25 ( 5.2%) | 452 ( 94.8%) |
| Musculoskeletal | 5 ( 2.5%) | 199 ( 97.5%) | 2 ( 0.5%) | 380 ( 99.5%) |
| Mental disorder | 19 (14.1%) | 116 ( 85.9%) | 16 ( 6.8%) | 218 ( 93.2%) |
| Diabetes | 4 ( 2.6%) | 147 ( 97.4%) | 0 ( 0.0%) | 179 (100.0%) |
| Respiratory | 5 ( 5.4%) | 88 ( 94.6%) | 4 ( 4.0%) | 96 ( 96.0%) |
| Stomach ulcer | 5 ( 6.3%) | 74 ( 93.7%) | 16 (13.0%) | 107 ( 87.0%) |
| Cancer | 0 ( 0.0%) | 22 (100.0%) | 0 ( 0.0%) | 62 (100.0%) |
| Dementia & Parkinson's | 0 ( 0.0%) | 14 (100.0%) | 3 (15.8%) | 16 ( 84.2%) |
| **Region of origin: Latin America** |  |  |  |  |
| Cardiovascular | 21 (13.4%) | 136 ( 86.6%) | 15 ( 7.2%) | 192 ( 92.8%) |
| Musculoskeletal | 2 ( 3.1%) | 62 ( 96.9%) | 5 ( 2.6%) | 187 ( 97.4%) |
| Mental disorder | 1 ( 1.5%) | 64 ( 98.5%) | 13 ( 9.0%) | 131 ( 91.0%) |
| Diabetes | 8 (13.1%) | 53 ( 86.9%) | 7 (12.7%) | 48 ( 87.3%) |
| Respiratory | 4 (18.2%) | 18 ( 81.8%) | 0 ( 0.0%) | 37 (100.0%) |
| Stomach ulcer | 5 (41.7%) | 7 ( 58.3%) | 8 (21.6%) | 29 ( 78.4%) |
| Cancer | 2 ( 6.2%) | 30 ( 93.8%) | 2 ( 5.6%) | 34 ( 94.4%) |
| Dementia & Parkinson's | 0 ( 0.0%) | 7 (100.0%) | 0 ( 0.0%) | 7 (100.0%) |
| **Region of origin: Eastern Europe** |  |  |  |  |
| Cardiovascular | 91 ( 3.6%) | 2,448 ( 96.4%) | 204 ( 4.9%) | 3,921 ( 95.1%) |
| Musculoskeletal | 68 ( 5.3%) | 1,220 ( 94.7%) | 117 ( 3.8%) | 2,939 ( 96.2%) |
| Mental disorder | 20 ( 3.4%) | 568 ( 96.6%) | 66 ( 3.5%) | 1,828 ( 96.5%) |
| Diabetes | 12 ( 1.9%) | 636 ( 98.1%) | 21 ( 1.6%) | 1,288 ( 98.4%) |
| Respiratory | 38 ( 8.6%) | 403 ( 91.4%) | 95 (10.8%) | 781 ( 89.2%) |
| Stomach ulcer | 62 (10.6%) | 522 ( 89.4%) | 35 ( 4.0%) | 833 ( 96.0%) |
| Cancer | 2 ( 0.8%) | 263 ( 99.2%) | 9 ( 1.8%) | 499 ( 98.2%) |
| Dementia | 4 ( 3.9%) | 98 ( 96.1%) | 0 ( 0.0%) | 154 (100.0%) |
| **Region of origin: Other Europe & North America** |  |  |  |  |
| Cardiovascular | 189 ( 5.3%) | 3,401 ( 94.7%) | 199 ( 4.7%) | 4,053 ( 95.3%) |
| Musculoskeletal | 89 ( 5.0%) | 1,684 ( 95.0%) | 159 ( 4.3%) | 3,539 ( 95.7%) |
| Mental disorder | 93 ( 8.2%) | 1,046 ( 91.8%) | 203 ( 8.3%) | 2,244 ( 91.7%) |
| Diabetes | 29 ( 2.3%) | 1,206 ( 97.7%) | 28 ( 2.8%) | 988 ( 97.2%) |
| Respiratory | 58 ( 8.1%) | 662 ( 91.9%) | 87 ( 9.3%) | 852 ( 90.7%) |
| Stomach ulcer | 61 (11.0%) | 495 ( 89.0%) | 26 ( 3.4%) | 740 ( 96.6%) |
| Cancer | 19 ( 3.6%) | 512 ( 96.4%) | 10 ( 1.3%) | 750 ( 98.7%) |
| Dementia & Parkinson's | 4 ( 2.3%) | 170 ( 97.7%) | 0 ( 0.0%) | 156 (100.0%) |
| **Region of residence: Northern Europe** |  |  |  |  |
| Cardiovascular | 10 ( 2.1%) | 461 ( 97.9%) | 29 ( 4.4%) | 631 ( 95.6%) |
| Musculoskeletal | 2 ( 0.9%) | 227 ( 99.1%) | 39 ( 7.1%) | 512 ( 92.9%) |
| Mental disorder | 32 (20.1%) | 127 ( 79.9%) | 20 ( 5.5%) | 343 ( 94.5%) |
| Diabetes | 0 ( 0.0%) | 148 (100.0%) | 2 ( 1.7%) | 118 ( 98.3%) |
| Respiratory | 9 (11.5%) | 69 ( 88.5%) | 13 ( 6.5%) | 187 ( 93.5%) |
| Stomach ulcer | 4 ( 5.3%) | 72 ( 94.7%) | 13 (14.6%) | 76 ( 85.4%) |
| Cancer | 0 ( 0.0%) | 73 (100.0%) | 4 ( 3.3%) | 117 ( 96.7%) |
| Dementia & Parkinson's | 0 ( 0.0%) | 38 (100.0%) | 0 ( 0.0%) | 19 (100.0%) |
| **Region of residence: Western Europe** |  |  |  |  |
| Cardiovascular | 156 ( 4.6%) | 3,207 ( 95.4%) | 234 ( 6.1%) | 3,600 ( 93.9%) |
| Musculoskeletal | 100 ( 5.3%) | 1,802 ( 94.7%) | 174 ( 4.6%) | 3,601 ( 95.4%) |
| Mental disorder | 63 ( 5.5%) | 1,073 ( 94.5%) | 226 ( 8.8%) | 2,347 ( 91.2%) |
| Diabetes | 31 ( 2.6%) | 1,184 ( 97.4%) | 49 ( 4.4%) | 1,054 ( 95.6%) |
| Respiratory | 54 ( 7.4%) | 676 ( 92.6%) | 113 (12.0%) | 828 ( 88.0%) |
| Stomach ulcer | 67 (12.7%) | 460 ( 87.3%) | 44 ( 5.8%) | 709 ( 94.2%) |
| Cancer | 13 ( 2.7%) | 475 ( 97.3%) | 12 ( 1.6%) | 734 ( 98.4%) |
| Dementia & Parkinson's | 0 ( 0.0%) | 110 (100.0%) | 3 ( 1.9%) | 156 ( 98.1%) |
| **Region of residence: Southern Europe** |  |  |  |  |
| Cardiovascular | 75 ( 5.3%) | 1,345 ( 94.7%) | 105 ( 6.3%) | 1,561 ( 93.7%) |
| Musculoskeletal | 14 ( 3.4%) | 400 ( 96.6%) | 15 ( 1.4%) | 1,067 ( 98.6%) |
| Mental disorder | 24 ( 6.5%) | 344 ( 93.5%) | 48 ( 6.6%) | 683 ( 93.4%) |
| Diabetes | 24 ( 4.7%) | 486 ( 95.3%) | 21 ( 4.8%) | 414 ( 95.2%) |
| Respiratory | 19 ( 8.5%) | 204 ( 91.5%) | 14 ( 5.5%) | 241 ( 94.5%) |
| Stomach ulcer | 29 (14.1%) | 176 ( 85.9%) | 14 ( 5.1%) | 258 ( 94.9%) |
| Cancer | 8 ( 4.6%) | 167 ( 95.4%) | 3 ( 1.4%) | 214 ( 98.6%) |
| Dementia & Parkinson's | 4 ( 4.8%) | 80 ( 95.2%) | 0 ( 0.0%) | 62 (100.0%) |
| **Region of residence: Eastern Europe** |  |  |  |  |
| Cardiovascular | 85 ( 4.3%) | 1,909 ( 95.7%) | 117 ( 3.4%) | 3,358 ( 96.6%) |
| Musculoskeletal | 55 ( 5.0%) | 1,048 ( 95.0%) | 76 ( 3.0%) | 2,442 ( 97.0%) |
| Mental disorder | 20 ( 4.4%) | 437 ( 95.6%) | 43 ( 2.9%) | 1,457 ( 97.1%) |
| Diabetes | 8 ( 1.6%) | 488 ( 98.4%) | 12 ( 1.1%) | 1,121 ( 98.9%) |
| Respiratory | 33 ( 8.4%) | 362 ( 91.6%) | 61 ( 9.0%) | 614 ( 91.0%) |
| Stomach ulcer | 39 ( 7.6%) | 472 ( 92.4%) | 20 ( 2.5%) | 775 ( 97.5%) |
| Cancer | 2 ( 1.1%) | 178 ( 98.9%) | 3 ( 0.8%) | 381 ( 99.2%) |
| Dementia & Parkinson's | 4 ( 5.3%) | 72 ( 94.7%) | 0 ( 0.0%) | 117 (100.0%) |


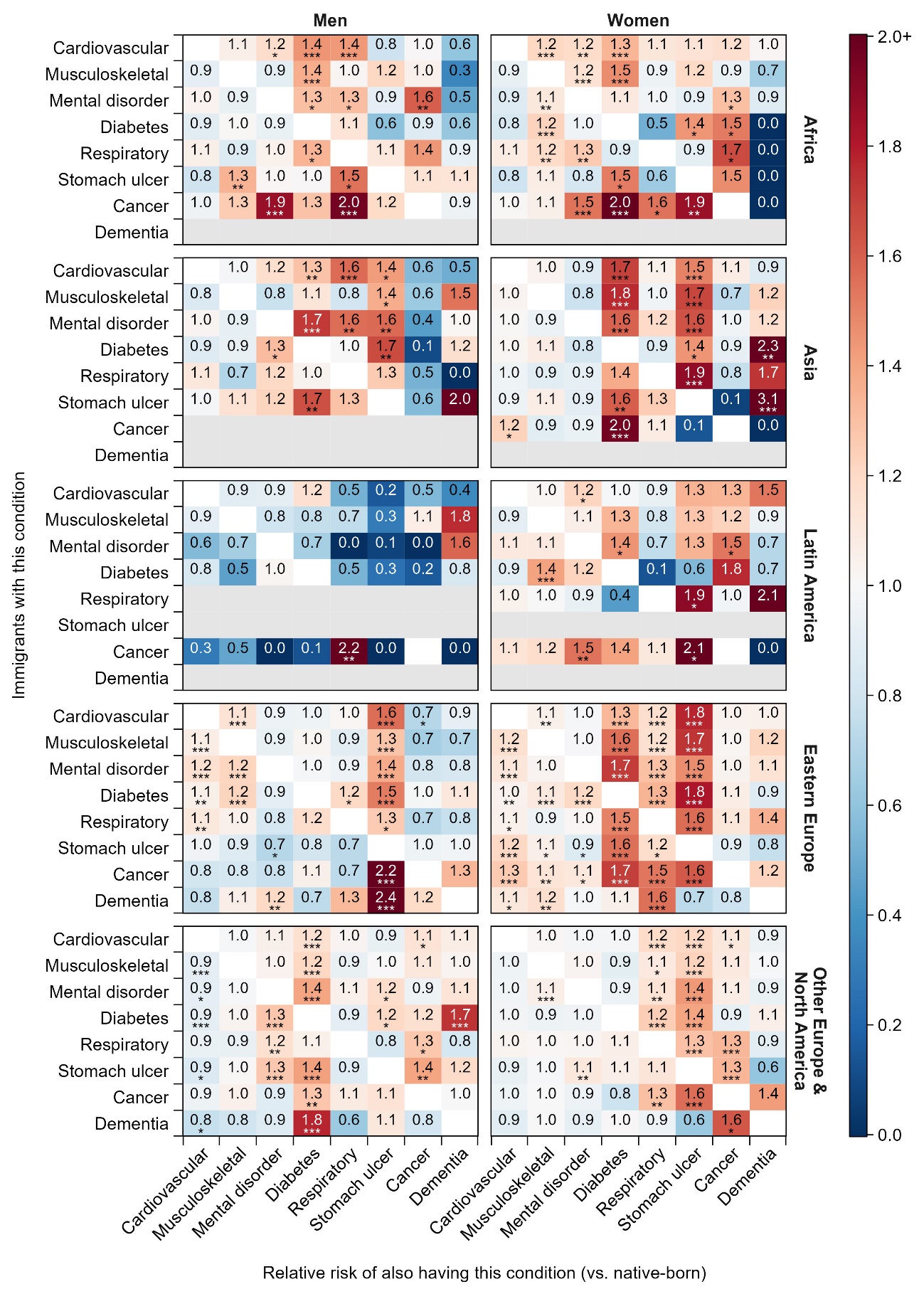


## **Fig. S2** Relative risks of multimorbidity among immigrants versus native-born individuals by gender and region of origin based on conditions developed post-migration

* p < 0.05; ** p < 0.01; *** p < 0.001


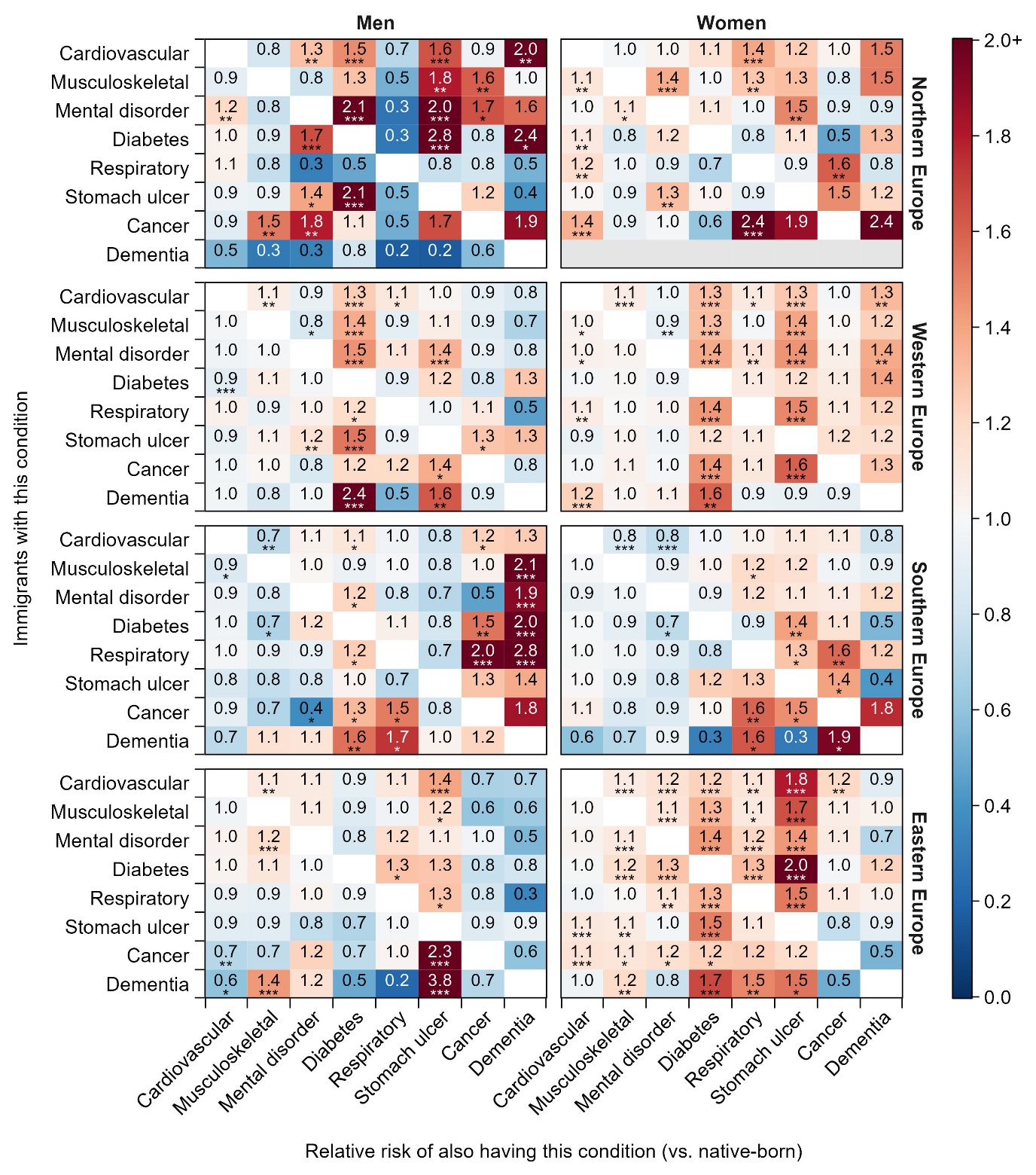


## **Fig. S3** Relative risks of multimorbidity among immigrants versus native-born individuals by gender and region of residence region based on conditions developed post-migration

* p < 0.05; ** p < 0.01; *** p < 0.001

# Section VI: Supplemental Analysis Including Individuals Aged 80 and Above

## **Table S11.** Number and percentage of individuals with eight chronic diseases among samples aged 50–79 years versus those aged 80 years and above by gender

|  | **Age 50-79** | |  | **Age 80+** | |  |
| --- | --- | --- | --- | --- | --- | --- |
|  | **Native-born** | **Immigrant** |  | **Native-born** | **Immigrant** |  |
| **Men** |  |  |  |  |  |  |
| Total sample | 142,287 (100.0%) | 12,781 (100.0%) |  | 21,146 (100.0%) | 1,770 (100.0%) |  |
| With cardiovascular diseases | 83,463 ( 58.7%) | 7,437 ( 58.2%) |  | 17,522 ( 82.9%) | 1,505 ( 85.0%) | * |
| With musculoskeletal diseases | 38,982 ( 27.4%) | 3,734 ( 29.2%) | *** | 9,718 ( 46.0%) | 893 ( 50.5%) | *** |
| With mental disorder | 23,067 ( 16.2%) | 2,210 ( 17.3%) | ** | 5,284 ( 25.0%) | 483 ( 27.3%) | * |
| With diabetes | 24,142 ( 17.0%) | 2,431 ( 19.0%) | *** | 4,973 ( 23.5%) | 415 ( 23.4%) |  |
| With respiratory diseases | 14,654 ( 10.3%) | 1,476 ( 11.5%) | *** | 3,734 ( 17.7%) | 333 ( 18.8%) |  |
| With cancer | 10,591 ( 7.4%) | 949 ( 7.4%) |  | 3,077 ( 14.6%) | 284 ( 16.0%) | * |
| With stomach ulcer | 11,401 ( 8.0%) | 1,356 ( 10.6%) | *** | 2,380 ( 11.3%) | 258 ( 14.6%) | *** |
| With dementia & Parkinson's | 3,294 ( 2.3%) | 314 ( 2.5%) |  | 2,425 ( 11.5%) | 216 ( 12.2%) |  |
| **Women** |  |  |  |  |  |  |
| Total sample | 178,495 (100.0%) | 16,929 (100.0%) |  | 30,408 (100.0%) | 2,943 (100.0%) |  |
| With cardiovascular diseases | 98,282 ( 55.1%) | 9,940 ( 58.7%) | *** | 25,701 ( 84.5%) | 2,564 ( 87.1%) | *** |
| With musculoskeletal diseases | 80,462 ( 45.1%) | 8,161 ( 48.2%) | *** | 21,171 ( 69.6%) | 2,048 ( 69.6%) |  |
| With mental disorder | 52,593 ( 29.5%) | 5,352 ( 31.6%) | *** | 13,125 ( 43.2%) | 1,328 ( 45.1%) | * |
| With diabetes | 24,383 ( 13.7%) | 2,876 ( 17.0%) | *** | 6,979 ( 23.0%) | 677 ( 23.0%) |  |
| With respiratory diseases | 18,117 ( 10.1%) | 2,130 ( 12.6%) | *** | 4,772 ( 15.7%) | 520 ( 17.7%) | ** |
| With cancer | 15,403 ( 8.6%) | 1,508 ( 8.9%) |  | 3,312 ( 10.9%) | 355 ( 12.1%) | * |
| With stomach ulcer | 13,725 ( 7.7%) | 1,955 ( 11.5%) | *** | 3,527 ( 11.6%) | 459 ( 15.6%) | *** |
| With dementia & Parkinson's | 3,698 ( 2.1%) | 366 ( 2.2%) |  | 3,990 ( 13.1%) | 347 ( 11.8%) | * |

* p < 0.05; ** p < 0.01; *** p < 0.001

*Note:* Statistical significance was determined using chi-square tests.


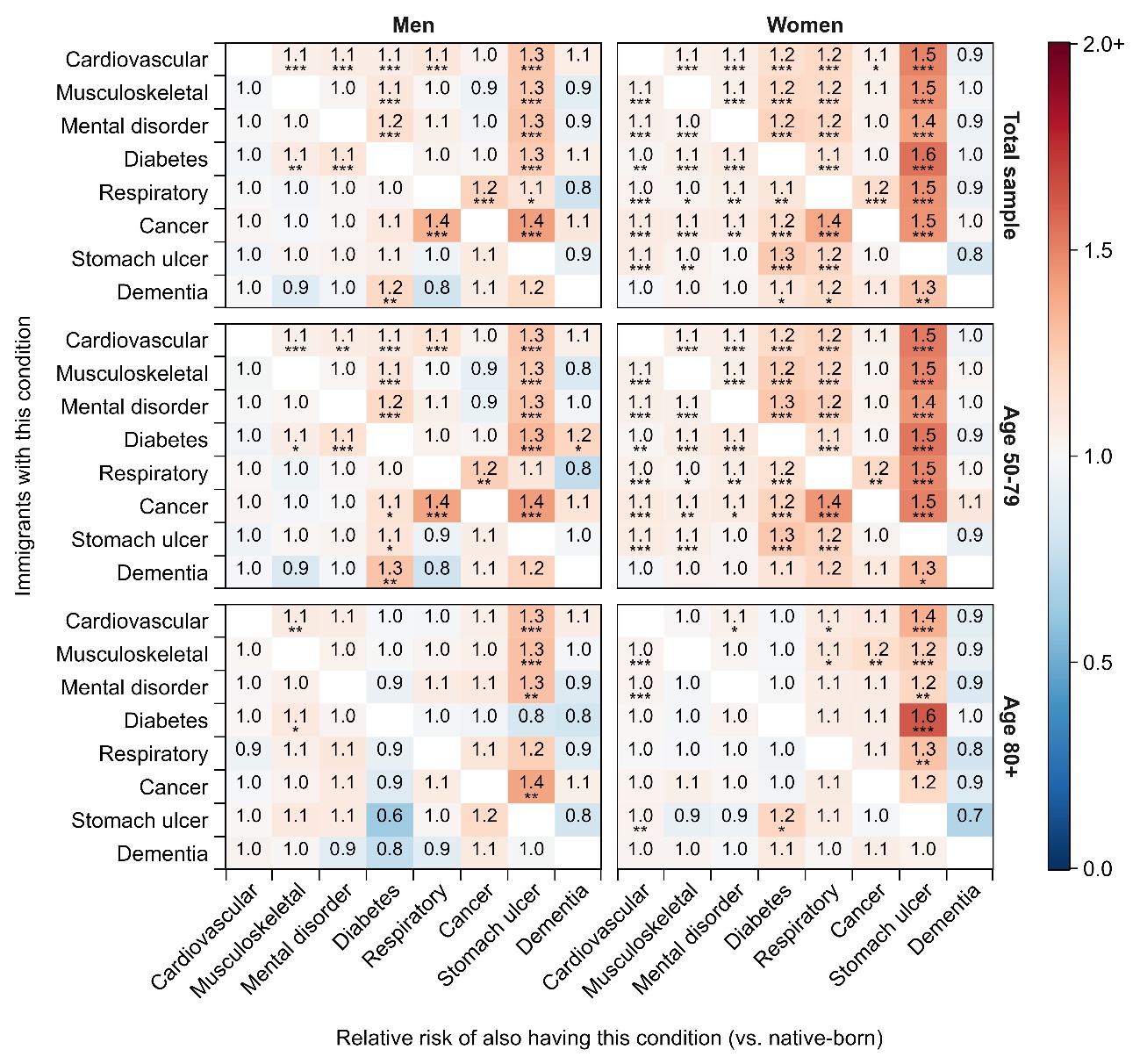


## **Fig. S4** Relative risks of multimorbidity among immigrants versus native-born individuals by gender in samples including aged 80 and above

* p < 0.05; ** p < 0.01; *** p < 0.001

# Section VII: Supplemental Analysis on Balanced Panel


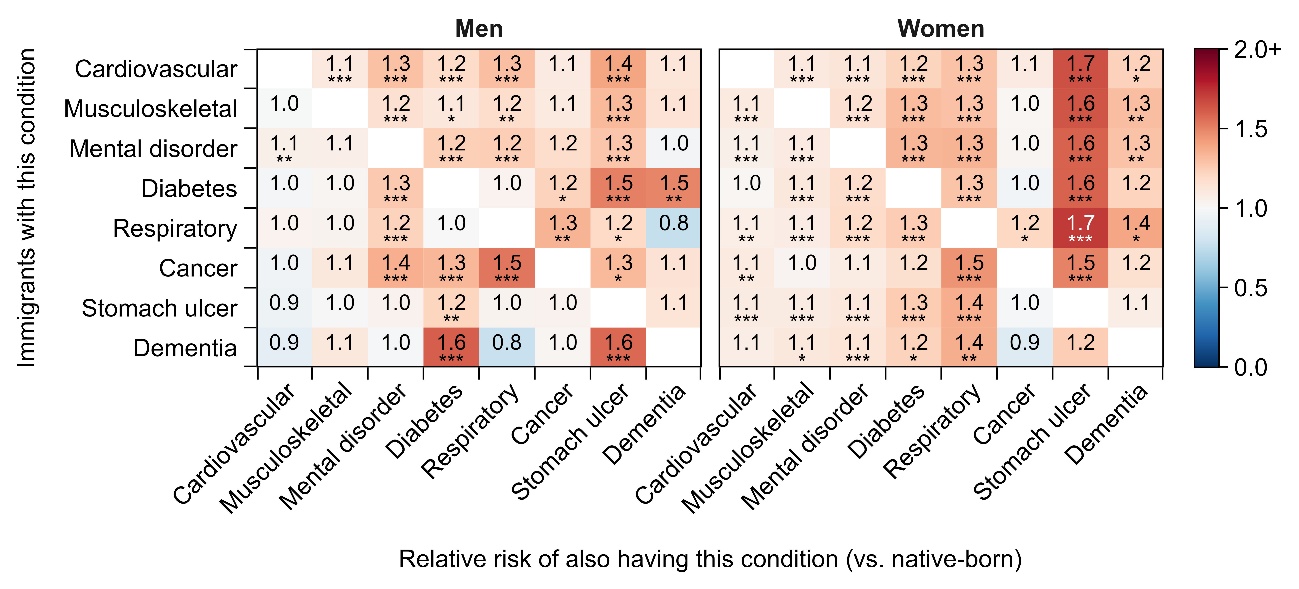


## **Fig. S5** Relative risks of multimorbidity among immigrants versus native-born individuals by gender in Waves 7 onward

* p < 0.05; ** p < 0.01; *** p < 0.001
